# HDAC6 Inhibition Releases HR23B to Activate Proteasomes, Expand the Tumor Immunopeptidome and Amplify T-cell Antimyeloma Activity

**DOI:** 10.1158/2767-9764.CRC-23-0528

**Published:** 2024-06-18

**Authors:** Priyanka S. Rana, James J. Ignatz-Hoover, Byung-Gyu Kim, Ehsan Malek, Yuriy Federov, Drew Adams, Timothy Chan, James J. Driscoll

**Affiliations:** 1 Case Comprehensive Cancer Center, School of Medicine, Case Western Reserve University, Cleveland, Ohio.; 2 Division of Hematology and Oncology, Department of Medicine, Case Western Reserve University, Cleveland, Ohio.; 3 Adult Hematologic Malignancies and Stem Cell Transplant Section, Seidman Cancer Center, University Hospitals Cleveland Medical Center, Cleveland, Ohio.; 4 Small Molecule Drug Discovery Core, Case Western Reserve University, Cleveland, Ohio.; 5 Cleveland Clinic, Lerner Research Institute, Cleveland, Ohio.

## Abstract

Proteasomes degrade intracellular proteins to generate antigenic peptides that are recognized by the adaptive immune system and promote anticancer immunity. However, tumors subvert the antigen presentation machinery to escape immunosurveillance. We hypothesized that proteasome activation could concomitantly increase antigen abundance and diversity in multiple myeloma cells. High-throughput screens revealed that histone deacetylase 6 (HDAC6) inhibitors activated proteasomes to unmask neoantigens and amplify the tumor-specific antigenic landscape. Treatment of patient CD138^+^ cells with HDAC6 inhibitors significantly promoted the antimyeloma activity of autologous CD8^+^ T cells. Pharmacologic blockade and genetic ablation of the HDAC6 ubiquitin-binding domain released HR23B, which shuttles ubiquitinylated cargo to proteasomes, while silencing *HDAC6* or *HR23B* in multiple myeloma cells abolished the effect of HDAC6 inhibitors on proteasomes, antigen presentation, and T-cell cytotoxicity. Taken together, our results demonstrate the paradigm-shifting translational impact of proteasome activators to expand the myeloma immunopeptidome and have revealed novel, actionable antigenic targets for T cell–directed immunotherapy.

**Significance:**

The elimination of therapy-resistant tumor cells remains a major challenge in the treatment of multiple myeloma. Our study identifies and functionally validates agents that amplify MHC class I–presented antigens and pave the way for the development of proteasome activators as immune adjuvants to enhance immunotherapeutic responses in patients with multiple myeloma.

## Introduction

Multiple myeloma is characterized by the clonal proliferation of malignant plasma cells in the bone marrow microenvironment, monoclonal protein in the blood and/or urine, and associated organ dysfunction (1). In the United States, multiple myeloma accounts for 1.8% of new cancer cases and 13% of hematologic cancers (2). Multistep genetic and microenvironmental changes lead to the transformation of these cells into a genetically heterogeneous malignant neoplasm. Deep sequencing has revealed significant genetic primary and secondary events in multiple myeloma, while intratumor genetic heterogeneity is common (3). Genetic heterogeneity precludes the identification of universally actionable events and limits the efficacy of targeted therapies. While options for targeted therapy are limited, multiple myeloma is sensitive to immunomodulators, especially those that reverse multiple myeloma–induced immunosuppression. The introduction of autologous stem cell transplantation, bortezomib, and immunomodulatory drugs have changed myeloma management and improved the 5-year survival to approximately 60% (3).

Proteasomes are structurally heterogeneous, dynamically regulated protein degrading machines that are responsible for the bulk of controlled protein degradation in eukaryotic cells (4). The high selectivity and tight control required for intracellular proteolysis is accomplished by ubiquitination of appropriate substrates for degradation and through the complex architecture of the 26S proteasome holoenzyme (5). The holoenzyme's proteolytic active sites reside within a chamber of the barrel-shaped 20S protein-hydrolyzing core particle (CP) and are accessible only through narrow axial pores (6). Gating of these pores is controlled by regulatory particles (RP), for example, PA28αβ (11S RP), PA28γ, PA200, and the 19S RP (PA700; ref. 7). 19S RPs associate with the 20S CP in an ATP-dependent manner to form 26S proteasomes and translocates substrates into the degradation chamber (8). Numerous accessory proteins, for example, HR23/Rad23, Dsk2, ECM29, have been identified that physically and functionally associate with 26S complexes (9–11).

The majority of defined antitumor T-cell responses require proteasomal processing of intracellular proteins to generate peptides that are bound to MHC class I (MHC-I) molecules and presented to antigen-specific T cells (12). The proteasome multicatalytic protein-hydrolyzing 20S CP consists of 28 subunits assembled as four stacked, heptameric rings. The two outer rings are identical and contain seven different α-subunits, whereas the two identical inner rings contain seven different β-subunits, three of which, β1, β2, and β5, exert catalytic activities (13). Subunit β1 cleaves predominantly after acidic residues (peptidylglutamyl-like activity), subunit β2 after basic residues (trypsin-like, T-like activity), and subunit β5 after hydrophobic residues (chymotrypsin-like, ChT-like activity). Each constitutive proteasome harbors six proteolytically active subunits (β1, β2, β5), while β5 was identified as rate-limiting and is a primary target of clinically available proteasome inhibitors, now the backbone of current antimyeloma therapy (14). Peptides generated by the proteasome are generally 8–10 amino acids in length and, in most cases, mirror the linear sequence of the parental protein.

The catalytic properties of proteasome subtypes are determined by their catalytic subunit composition. To process antigens more efficiently, constitutive proteasome catalytic subunits are replaced with the specialized catalytic subunits (β1i, β2i, and β5i) that are incorporated to form immunoproteasomes (ref. 15; Supplementary Fig. S1). Immunoproteasomes display greater ChT-like activity relative to constitutive proteasomes and the processing of antigenic peptides is favored in cells that harbor immunoproteasomes to shape the diverse repertoire of immunodominant epitopes (16). These findings suggest the presence of a highly sophisticated proteolytic machinery with the functional plasticity to generate finely tailored peptides. Immunoproteasome upregulation is associated with improved response to immune checkpoint inhibitors (17, 18).

Several methods have identified drugs and small molecules that modulate proteasome activity and proteasomes have been pharmacologically targeted to treat human diseases (19–22). Here, we discovered that histone deacetylase 6 (HDAC6)-selective inhibitors not only increased proteasome activity but enhanced antigen presentation and promoted CTL-mediated tumor lysis. Mechanistic studies revealed that upon HDAC6 inhibition or genetic ablation, the Ub-like (UBL) protein HR23B which interacts with the HDAC6 C-terminal ubiquitin-binding (BUZ) domain, is released and associates with proteasomes. HR23B-mediated proteasome activation increases MHC-I antigen presentation and dictates cytotoxic T-cell antitumor activity.

## Materials and Methods

### High-throughput Screen

A library of 3,400 FDA-approved agents and bioactive molecules was screened at the Case Comprehensive Cancer Center (Case CCC), Small Molecule Drug Development Core. RPMI-8226 cells (30,000/well) were incubated in 30 µL with each agent at a final concentration of 3 µmol/L in 384-well plates. Cells were incubated with agents for 21 hours under standard conditions. The proteasome substrate LLVY-R110 was then added and incubated with cells for 18 hours. Fluorescence was determined at excitation of 490 nmol/L and emission of 525 nmol/L. Assay of multiple myeloma culture supernatants indicated no hydrolysis of LLVY-R110 under the same conditions.

### Cell Lines

All indicated cell lines were authenticated and obtained from ATCC and cultured according to the resource center's guidelines. Cells used in all assays were cultured for no more than five passages. *HDAC6*, *HR23A*, and *HR23B*-knockout (KO) cells were generated by using electroporation with single-guide RNA (sgRNA)/Cas9 mixes (Synthego), according to the manufacturer's instructions. For each human gene, a pool of three verified sgRNAs was used (Synthego). Scrambled/nontargeting (NT) sgRNAs (Synthego) were used a negative control. Efficient and stable gene KO was verified by Western blot analysis. If KO efficiency was <80%, a second round of sgRNA delivery is performed. Generation of HeLa *HDAC6*-KO cells overexpressing pcDNA-HDAC6-FLAG, pcDNA-HDAC6.DC-FLAG, or pcDNA-HDAC6.ΔBuz-FLAG was achieved using lipofectamine 3000 (Thermo Fisher Scientific, catalog no. L3000001). After transfection, the cells were incubated for 48 hours. A Western blot analysis was performed to determine the level of expression. Generation of *RPMI-8226* wildtype (WT) and *HDAC6*-KO overexpressing Flag-HA-PSMD14 (RRID: Addgene #22557) were generated using a viral transduction method. Phoenix E cells were cotransfected with Flag-HA-PSMD14 (plasmid #22557) and helper plasmid pCL-Eco (RRID: Addgene_12371) using lipofectamine-3000 (Thermo Fisher Scientific, catalog no. L3000008). Cells were incubated for 48 hours for peak plasmid expression. Media containing virus was spun 10 minutes at 2,000 rpm, supernatant collected and filtered through a 45-µm pore filter to remove debris. Fresh retroviral suspension mixed with 8 µg/mL Polybrene and overlayed on RPMI-8226 cells. After 9 hours, media was replaced with fresh RPMI1640 and cells were incubated for 48 hours for peak plasmid expression after which they were selected with 2 µg/mL puromycin for 72 hours. FLAG-HA-PSMD14 expression was analyzed using a mouse monoclonal anti-FLAG M2 antibody (Millipore Sigma, catalog no. F1804-200UG). Cell lines were grown in complete medium supplemented with 10% FBS and 5% pen/strep. Transfected cell lines were selected in complete medium with selection antibiotic.

### Measurement of Proteasome ChT-like Activity

A homogeneous fluorescent assay was used that measures proteasome ChT-like activity in cultured cells. The proteasome ChT-like substrate LLVY-R110 was added for indicated times and concentrations (50,000 cells/well).

### Measurement of Cell Viability

Cell viability was determined using the XTT assay (23). Briefly, 50,000 cells were added to 96-well plates in a volume of 250 µL in RPMI media that lacked phenol red. Drugs were added at indicated concentrations and plates incubated under standard conditions for 72 hours. XTT sodium salt tetrazolium reagent (Gold Biotechnology, catalog no. X-200) was diluted in PBS (1 mg/mL) containing phenazine methosulfate (PMS, 7.5 µg/mL). Cells were incubated with the XTT+PMS reagent for 3 hours at 37°C and absorbance was measured at 450 nm using a Molecular Devices i3x Spectramax plate reader.

### Quantitation of SIINFEKL–MHC-I Complex

E.G7-Ova or EL.4 cells were incubated with drugs as indicated for up to 72 hours. Cells were washed thrice, pelleted, washed, and stained with mouse PE-conjugated mAb in staining buffer. PE-positive cells were quantitated using a Attune Nxt interfaced with FlowJo software (FlowJo, RRID:SCR_008520).

### B3Z T-cell Hybridoma Assay

E.G7-Ova or EL.4 cells were incubated with pharmacologics as indicated for 72 hours. E.G7-Ova cells were incubated with pharmacologics (3 µmol/L) for 72 hours, washed thrice, resuspended in fresh media and cocultured with B3Z cells for 18 hours at an effector:target (E:T) of 2:1. Cells were then stained with propidium iodide to detect dead cells and an APC-conjugated, anti-mouse CD8a antibody to detect CD8^+^ cells. Cells were washed twice with fresh buffer, resuspended with T-cell medium, and counted. Variable numbers of target cells were mixed with the effector B3Z cells in triplicate and cultured in 96-well round bottomed tissue culture plates. After 12 hours, cells were washed with FACS buffer twice and stained with annexin-V conjugated with FITC and anti-CD8 conjugated with APC (BD Biosciences, BDB553035). Stained cells were washed with FACS buffer twice and stained with 7-actinomycin-D (7-AAD), according to the manufacturer's instructions. CD8-negative cells were analyzed for annexin-V and 7-AAD by flow cytometry.

### Measurement of Proteasome Peptide-hydrolyzing Activity

RPMI-8226 cells were treated with pharmacologics at 3 µmol/L for 72 hours, pelleted, lysed using Cel-Lytic M and centrifuged at 100,000 × *g* for 15 minutes at 4°C. Supernatants were removed and used as cell lysates. Assays were performed in Tris-HCl (20 mmol/L, pH 7.6), 5 mmol/L MgCl_2_, 2 mmol/L ATP, 1 mmol/L DTT, and 10% glycerol. Substrates were added at final concentrations of 50 µmol/L, except Suc-LLVY-MCA added at 100 µmol/L. Lysates were incubated with substrates at 37°C for 60 minutes. Fluorescence was measured using a Molecular Devices Spectramax plate reader.

### Western Blotting

RPMI-8226 cells were treated with HDAC6 inhibitors, cells were harvested in 20 mmol/L Tris pH 7.6, 25 mmol/L KCl, 10 mmol/L NaCl, 3 mmol/L MgCl_2_, 0.1 mmol/L ethylenediaminetetraacetic acid (EDTA), 10 mmol/L ATP, and 10% glycerol and lysed by freeze/thaw cycles. After centrifugation (15 minutes, 20,000 × *g*), the supernatant protein concentration was determined the Pierce™ 660nm Protein Assay Reagent (24). Samples were loaded on 4%–12% precast 1 mm bis-tris gels (Bio-Rad) and electrophoresed for 1 hour at 180 V. Proteins were transferred to polyvinylidene difluoride (PVDF) membranes (Millipore) for 1 hour at 4°C and stained with primary antibodies diluted 1:1,000. Membranes were stained with LI-COR secondary antibodies at 1:5,000–1:10,000 (LI-COR Biosciences, catalog no. 926-68023, RRID:AB 10706167). Secondary antibodies were IR dye 680LT donkey anti-rabbit (red) 926-68023, IR dye 800 CW donkey anti-rabbit (green) 926-32213, and IR dye 800 CW goat anti-mouse (green; LI-COR Biosciences, catalog no. 926-32210 RRID:AB 621842). Images were obtained using an Odyssey CLx system for LI-COR applications.

### Effect of Nonspecific HDAC Inhibitors on Proteasome Activity

RPMI-8226, ARH-77, and U266 cells were treated with tubastatin-A, ACY-738, and ACY-1215 for indicated times at the indicated concentrations. Proteasome activity was measured at 21 hours using LLVY-R110. Cells were treated with the nonspecific or pan-HDAC inhibitors vorinostat, panobinostat, and romidepsin for 72 hours as indicated. The XTT assay was performed to evaluate cell viability after treatment.

### Immunoprecipitation

The anti-DYKDDDDK affinity resin (Thermo Fisher Scientific, catalog no. A36801) was used to immunoprecipitate proteins from *in vitro* purification system. Resin was washed twice with TBS, equilibrated to room temperature prior to use and 50 µL resin slurry added/sample. Samples were incubated for 2 hours at 4°C with end-over-end rotator. FLAG-tagged proteins were eluted using Pierce 3X DYKDDDDK peptide (150 ng/µL; Thermo Fisher Scientific, catalog no. A36805) in TBS with gentle shaking for 30 minutes. Samples were centrifuged, FLAG-immunoprecipitated proteins collected and assayed by Western blot analysis.

### Patient Samples

Patient samples were obtained following Institutional Review Board approval and written informed consent in accordance with recognized ethical guidelines under the Declaration of Helsinki. Peripheral blood samples were barcoded and stored at −80°C. BM samples were processed to isolate the mononuclear cell fraction by red blood cell lysis following by gradient centrifugation. CD138^+^ cells were isolated using the EasySep human CD138-positive selection kit II (StemCell Technology). CD8^+^ T cells were isolated using the EasySep direct human CD8^+^ T-cell isolation kit (StemCell Technology).

### Generation of CRISPR KO Cell Lines

Generation of *HDAC6, HR23A,* and *HR23B*-KO cells was achieved using electroporation of myeloma cells with sgRNA/Cas9 mixes (Synthego), according to the manufacturer's instructions. For each gene, a pool of three verified sgRNAs was used (Synthego). NT sgRNAs (Synthego) were used a negative control. Efficient and stable KO was verified by Western blot analysis. Guide RNA sequences for NT guide RNA, HDAC6-KO, HR23A-KO, and HR23B-KO are listed below:

Scrambled (control)#1 G*C*A*CUACCAGAGCUAACUCA + synthego modified Ez scaffold
HDAC6+sgRNA#1 C*A*A*CCAGGCAGCGAAGAAGU + synthego modified Ez Scaffold
HDAC6+sgRNA#2 G*G*G*CCUCACCGAAGUGACAC + synthego modified Ez Scaffold
HDAC6+sgRNA#3 C*A*G*AAGCGAAAUAUUAAAAA + synthego modified Ez Scaffold
HR23A+sgRNA#1 C*U*C*UGGCGGCAGGUGGGGGA + Synthego modified EZ Scaffold
HR23A+sgRNA#2 G*A*G*GACUCUGGGGCAGCUGU + Synthego modified EZ Scaffold
HR23+sgRNA#3 G*U* U*GCAGACCAAAGCCGGCC + Synthego modified EZ Scaffold
HR23B+sgRNA#1 U*U*C*CUCCACCACCACAACUG + synthego modified Ez Scaffold
HR23B+sgRNA#2 G*A*U*GGAUGCAGGUGUGGAAG – Synthego modified EZ Scaffold
HR23B+sgRNA#3 U*C*U*UGUUUAGCUGCACUAGC + Synthego modified E2 Scaffold.

### Immunopeptidome Analysis

E.G7-Ova cells (5 × 10^9^/assay) and RPMI-8226 cells (2 × 10^9^/assay) were treated with DMSO (0.05%), tubastatin-A, or ACY-738 (1 µmol/L) for 72 hours. RPMI-8226 cells were also treated with BTZ (10 mmol/L) for 18 hours. Cells were pelleted, washed, frozen, and pellets were shipped to MS Bioworks. Pellets were resuspended in lysis buffer (0.25% sodium deoxycholate, 200 mmol/L iodoacetamide, 1% N-octyl-b-D-thioglucoside, 1 mmol/L EDTA) containing protease inhibitor (1 mL lysis buffer/ 1 × 10^8^ cells). Lysates were centrifuged at 800 × *g* for 5 minutes and supernatant collected to which an additional 5 mL of lysis buffer was added and centrifuged at 20,000 × *g* for 60 minutes. Cell lysate was then mixed with 100 µL (2 mg Ab) of H-2 MHC-I M1/42 resin for murine immunopeptidome and MHC-I (W6/32) resin for human myeloma immunopeptidome and incubated overnight at 4°C with gentle rotation. The resin slurry was centrifuged at 800 × *g* for 5 minutes and washed once in a 2.5 mL lysis buffer then with wash buffer 2 (20 mmol/L Tris-HCl, 400 mmol/L NaCl, pH 8.0) followed by one wash with buffer 3 (20 mmol/L Tris-HCl, 150 mmol/L NaCl, pH 8.0) and one wash with wash buffer 4 (20 mmol/L Tris-HCl, pH 8.0). A total of 1 mL MHC-I elution buffer was added to the pelleted resin and incubated for 5 minutes at 37°C. Peptides were concentrated and desalted using solid-phase extraction with Waters µHLB C18 plate. Peptides were loaded directly and eluted using 50/50 acetonitrile/water (0.1% trifluoroacetic acid [TFA]). Eluted peptides were lyophilized and reconstituted in 0.1% TFA.

### Mass Spectrometry

Peptides were analyzed by nano-LC/MS-MS using a Waters NanoAcquity system interfaced to a Thermo Fisher Scientific Fusion Lumos mass spectrometer. Peptides were loaded on a trapping column and eluted over a 75 µm analytic column at 350 nL/minute; both columns were packed with Luna C18 resin (Phenomenex) and a 2-hour gradient was employed. The mass spectrometer was operated using a custom data-dependent method, with mass spectrometry (MS) performed in the Orbitrap at 60,000 FWHM resolution and sequential MS-MS performed using high-resolution CID and EThcD in the Orbitrap at 15,000 FWHM resolution. All MS data were acquired from m/z 300–1,600. A 3-second cycle time was employed for all steps.

### MS Data Processing

Raw files were searched using a local copy of PEAKS with the following parameters: enzyme-none, database-Swissprot mouse+OVAL chick for murine immunopeptidome and Swissprot human for multiple myeloma immunopeptidome, fixed modification: none, variable modifications: oxidation (M), acetyl (protein N-terminus), carbamidomethyl (C), mass values: monoisotopic, peptide mass tolerance: 10 ppm, fragment mass tolerance: 0.02 Da, max missed cleavages: N/A, PSM FDR: 1%, and chimeric peptide: true. Data were further processed using Skyline v22.2.0.255. Tumor-associated antigens (TAA) were identified using https://caped.icp.ucl.ac.be/Peptide/list.

### Cell Line Authentication

Authenticated multiple myeloma cell lines (MMCL) were provided by ATCC and obtained from 2019 to 2023. Cells were immediately thawed and passaged in the appropriate media as indicated in the ATCC datasheet. Cell lines were checked for mycoplasma contamination using the Hoechst DNA stain (indirect) method.

### Statistical Analysis

Bioassays were performed in triplicate. Sex and age were considered as biological variables and randomized for analysis. Correlations were determined using Graphpad Prism (GraphPad Prism RRID:SCR_002798).

### Data Availability

Data and materials are available from James.Driscoll@UHhospitals.org and yxf204@case.edu.

## Results

### High-throughput Screening to Identify Pharmacologics That Increase Proteasome Activity

**FIGURE 1:**
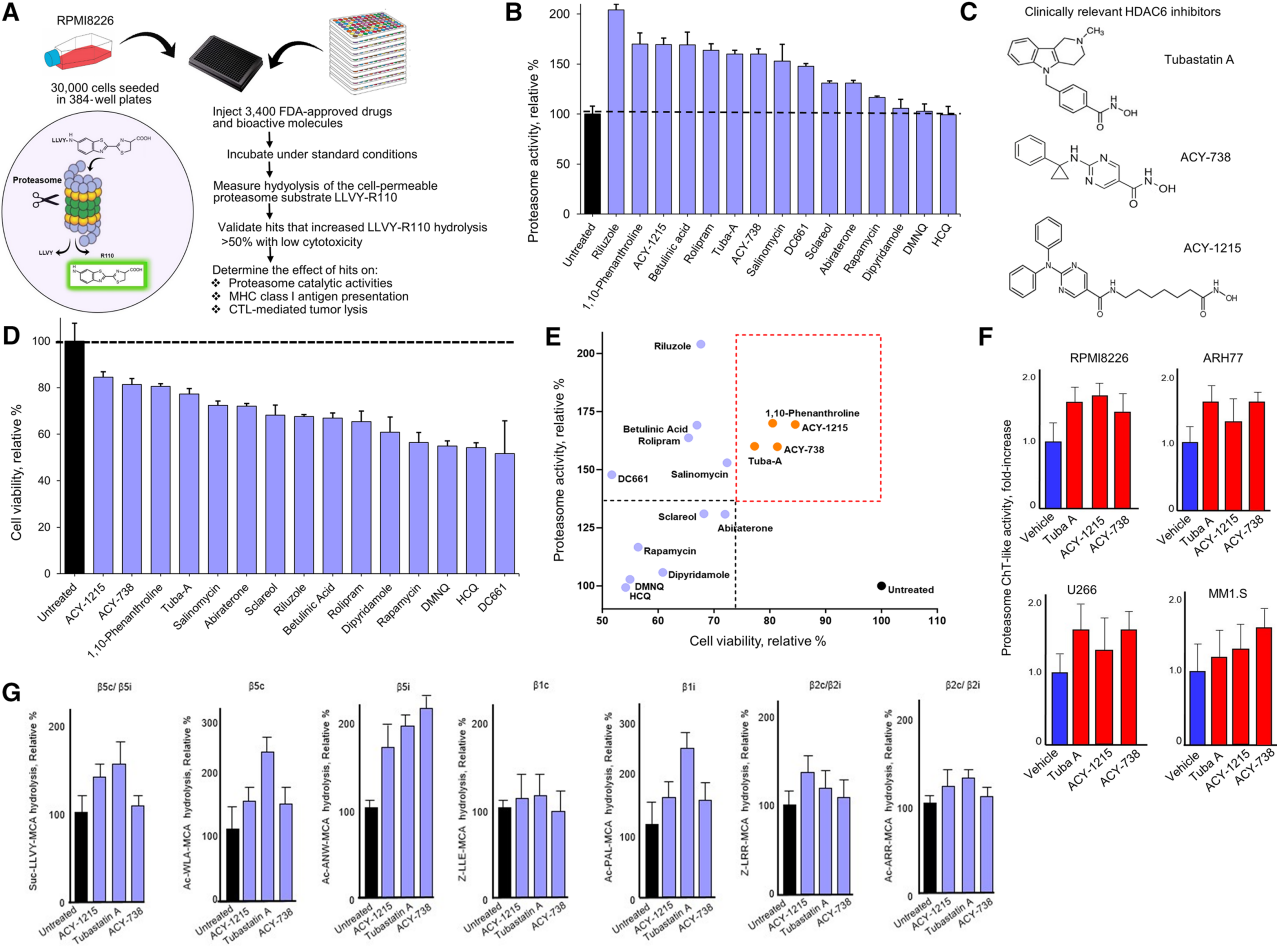
Cell-based HTS to identify pharmacologics that increased proteasome ChT-like activity. **A,** HTS scheme using the cell-permeable substrate LLVY-R110 to measure proteasome activity. LLVY-R110 is specifically cleaved by the proteasome to release the fluorogenic substrate R110 which fluoresces at an excitation of 490 nmol/L and emission 525 nmol/L. RPMI-8226 cells were incubated under standard culture conditions in 96-well plates in complete RPMI media that lacked phenol red. Pharmacologics (3,400) were then individually injected into each well at a final concentration of 3 µmol/L. Cells were incubated with pharmacologics for 21 hours. LLVY-R110 at a final concentration of 100 µmol/L was then added and incubated with cells for 18 hours under the same conditions. **B,** Rank-order effect of the top pharmacologics detected in the HTS that increased proteasome ChT-like activity. RPMI-8226 cells were incubated in 96-well plates with each pharmacologic at 1 µmol/L for 72 hours. Bioassays were performed in triplicate. Shown is the arithmetic average of triplicate measurements. Error bars indicate the SD of the mean. **C,** Effect of the top pharmacologics identified in the HTS on cell viability. Pharmacologics were added at 1 µmol/L for 16 hours and cell viability determined using the XTT assay (53). Shown is the arithmetic average of proteasome hydrolysis of LLVY-R110. **D,** Plot of the effect of pharmacologics on proteasome activity versus the effect on cell viability. Pharmacologics were prioritized based upon arbitrary cutoffs of >30% increase in proteasome activity and 75% cell viability. **E,** Chemical structure of clinically relevant HDAC6 inhibitors. **F,** Effect of HDAC6 inhibitors on proteasome ChT-like activity in MMCLs. Cells were incubated in 96-well plates with pharmacologics for 72 hours. Lysates were prepared in CelLyticM (Sigma) containing 20 mmol/L Tris-HCl (pH 7.6), 20 mmol/L NaCl, 10% glycerol, 3 mmol/L ATP, 5 mmol/L MgCl_2_, and 1 mmol/L DTT. Shown is the arithmetic average of triplicate measurements. Error bars indicate the SD of the mean. **G,** Effect of HDAC6 inhibitors on individual proteasome peptide-hydrolyzing activities. RPMI-8226 cells were incubated with HDAC6 inhibitors at 1 µmol/L for 72 hours, pelleted, washed thrice in cold PBS and lysed in CelLyticM containing 20 mmol/L Tris-HCl (pH 7.6), 20 mmol/L NaCl, 10% glycerol, 3 mmol/L ATP, 5 mmol/L MgCl_2_, and 1 mmol/L DTT. Fluorogenic peptide substrates were incubated with 5 µg of lysate for 1 hour at 37°C. All assays were performed in triplicate. Error bars indicate the relative SD.

We screened a library of 3,400 FDA-approved pharmacologics and bioactive molecules to identify agents that increased proteasome ChT-like activity (Fig. 1A). The cell-permeable, fluorogenic substrate LLVY-R110 is specifically cleaved by proteasomes to release the fluorescent moiety R110 and serves as a tool to directly measure the effects of drugs on proteasomes within intact cells. Pharmacologics that significantly increased ChT-like activity ≥20% comprised <1% of the total number of the agents screened (Supplementary Table S1). Our screen detected previously identified proteasome stimulators, for example, dipyridamole, rapamycin, and betulinic acid (19–22), as well as novel proteasome activators, for example, tubastatin-A, ACY-1215, ACY-738, abiraterone, DC661, salinomycin, sclareol, and riluzole. Generation of R110 fluorescence was directly proportional to cell number, incubation time, and substrate concentration, while at higher concentrations, DMSO reduced proteasome activity (Supplementary Fig. S2A–S2D). The rank-order effect of hits on proteasome ChT-like activity in RPMI-8226 cells is shown (Fig. 1B). The rank-order effect of hits that increased proteasome activity on cell viability indicated that most pharmacologics did not reduce cell viability ≥25% (Fig. 1C). Only four pharmacologics, including the HDAC6 inhibitors tubastatin-A, ACY-1215, ACY-738, and 1,10-phenanthroline, increased proteasome activity ≥30% and maintained cell viability ≥75% (Fig. 1D). Structures of the clinically-relevant HDAC6 inhibitors are shown (Fig. 1E). We focused our studies on the HDAC6 inhibitors because these agents were among the top hits detected in the high-throughput screen (HTS) and did not significantly reduce cell viability. HDAC6 inhibitors were shown to increase proteasome ChT-like activity in lysates prepared from a number of MMCLs (Fig. 1F). Structures of other hits that increased proteasome ChT-like activity are shown (Supplementary Fig. S3). The HDAC6 inhibitors also increased proteasome activity in ARH-77, U266, and MM1.S cells (Supplementary Fig. S4A). The HTS also detected known proteasome inhibitors, for example, bortezomib and carfilzomib, as well as agents that previously not been shown to inhibit proteasomes (Supplementary Table S2; Supplementary Fig. S4B). In contrast to the proteasome activators (Fig. 1A), proteasome inhibitors significantly reduced cell viability (Supplementary Fig. S5A and S5B). The specificity of the hits identified in the HTS for HDAC6, relative to other HDACs and SIRTs, was assessed using *in vitro*, dose-dependent, biochemical activity assays (Supplementary Fig. S6; Supplementary Table S3). At higher concentrations, tubastatin-A also inhibited HDAC10 biochemical activity, while ACY-1215 inhibited HDCA6 with 100-fold greater specificity relative to that measured against HDAC1, HDAC2, HDAC3, HDAC8, and HDAC10.

### Effect of HDAC6 Inhibitors on Proteasome Catalytic Activities

The effect of HDAC6 inhibitors on each proteasome catalytic activity in multiple myeloma cells was measured using peptide-based fluorescent reporters that are preferentially cleaved by the distinct catalytic sites (Fig. 1G; Supplementary Table S4). RPMI-S8226 cells were treated with the HDAC6 inhibitors, lysates prepared, incubated with each substrate and fluorescence measured. Treatment with the HDAC6 inhibitors increased hydrolysis of Suc-LLVY-MCA (cleaved by the β5c and β5i active sites) as well as hydrolysis of Anc-ANW-MCA that is preferentially cleaved by the β5i active site. The proteasome-related β1c, β2c, β1i, and β2i catalytic activities in lysates were not altered after treatment of cells with the HDAC6 inhibitors.

### Effect of Proteasome Activators on SIINFEKL–MHC-I Presentation

**FIGURE 2:**
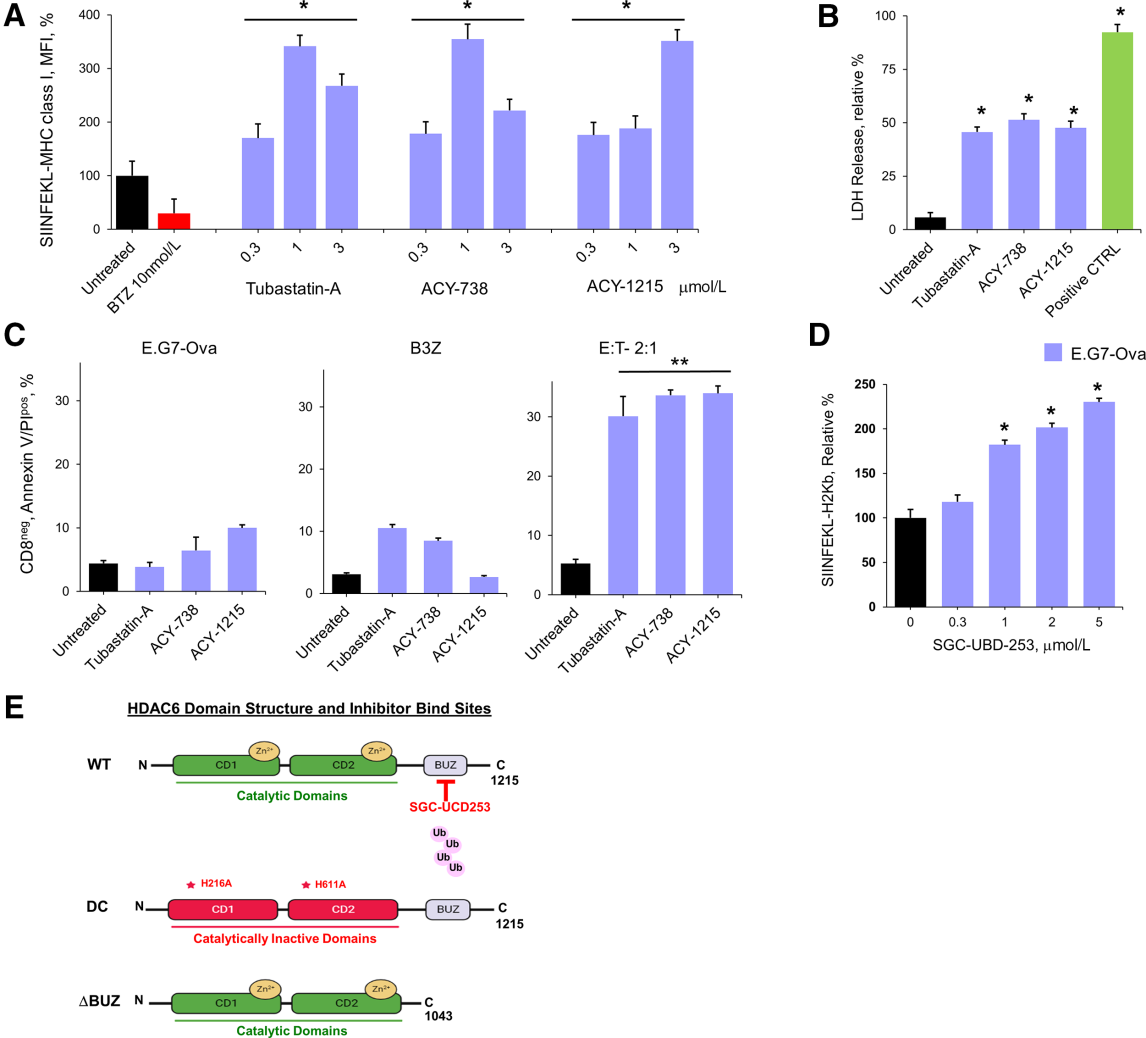
Effect of HDAC6 inhibitors on SIINFEKL presentation. **A,** Effect of HDAC6 inhibitors on SIINFEKL presentation. EL.4 lymphoma cells were transfected with the plasmid pAc-neo-Ova to generate the cell line E.G7-Ova. pAc-neo-Ova carries a complete copy of chicken *OVA* mRNA and the neomycin (G418) resistance gene. Proteasome degradation of Ova generates the peptide SIINFEKL that is then presented in a complex with the MHC-I molecule on E.G7-Ova cells. Shown is the relative effect of each HDAC6 inhibitor on the amount of SIINFEKL-H-2K^b^ molecule presented on the surface of treated cells normalized to untreated E.G7-Ova cells. Cells were also treated with bortezomib which reduced SIINFEKL-H-2K^b^ presentation and demonstrated that SIINFEKL presentation was proteasome dependent. **B,** Effect of HDAC6 inhibitors on LDH release. E.G7-Ova cells were treated with each agent at 3 µmol/L for 72 hours. Vehicle indicates E.G7-Ova cells treated with media alone. Positive control indicates E.G7-Ova cells treated with bortezomib at 10 nmol/L for 16 hours. After drug treatment, E.G7-Ova cells were incubated with B3Z cells at an E:T ratio of 2:1. Shown is the relative percent of LDH detected in culture media after 24 hours. **C,** Effect of HDAC6 inhibitors on CTL-mediated tumor lysis. E.G7-Ova cells were treated with each HDAC6 inhibitor at 1 µmol/L for 72 hours. Cells were also treated with bortezomib (10 nmol/L) for 16 hours. Untreated indicates E.G7-Ova cells treated with media alone. Cells were then washed, fresh media added and cells then cocultured with B3Z cells (E:T 2:1). CD8^neg^/annexin-V^+^/propidium iodide^+^ cells were then quantitated by flow cytometry. **D,** Effect of the HDAC6-BUZ domain specific inhibitor SGC-UBD253 on SIINFEKL-H-2K^b^ presentation on E.G7-Ova cells. Cells were treated with SGC-UBD253 at indicated concentrations and the level of SIINFEKL-H-2K^b^ quantitated by flow cytometry. All bioassays were performed in triplicate. Shown are averages of multiple experiments. Error bars represent the relative SD of the mean. **E,** Model of the HDAC6 domain structure and function and inhibitor binding sites. A *P*-value ≤ 0.05 is flagged with one star (*) and *P*-value ≤ 0.01 is flagged with two stars (**).

E.G7-Ova is a mouse lymphoma cell line derived from EL4 cells (C57BL/6, H-2K^b^, T lymphoma) and chicken ovalbumin (*OVA*) cDNA (25). E.G7-Ova cells constitutively synthesize Ova and are a validated model to study MHC-I–restricted CTL activity. Proteasomes degrade Ova to generate the peptide SIINFEKL (residues 257–264) that is presented by MHC-I molecules as a target for killing by Ova-specific hybridoma B3Z T cells (Supplementary Fig. S7; ref. 26). B3Z hybridoma T cells were engineered to express a T-cell receptor (TCR) specific for SIINFEKL that is presented by H-2K^b^ MHC-I alloantigen molecules on the surface of APCs and constitutes a unique T-cell hybridoma system to study tumor antigen-specific recognition. Preliminary experiments demonstrated that pharmacologics identified in the screen also increased proteasome activity in EL4 and E.G7-ova cells and increased SIINFEKL presentation on E.G7-Ova cells (Supplementary Fig. S8 and S9). E.G7-Ova cells were treated with the HDAC6 inhibitors and stained with an antibody specific for the SIINFEKL–MHC-I complex. Tubastatin-A, ACY-738, and ACY-1215 increased presentation of the class I molecule–SIINFEKL complex over 3-fold (Fig. 2A). In contrast, treatment of E.G7-Ova cells with bortezomib and other propidium iodides reduced SIINFEKL–MHC-I complex presentation (Fig. 2A; Supplementary Fig. S10).

### Effect of Proteasome Activators on CTL Activity

To determine whether the HDAC6 inhibitors that enhanced SIINFEKL presentation also promoted T cell–mediated tumor lysis, we employed the B3Z T-cell hybridoma system (27). The cytosolic enzyme lactate dehydrogenase (LDH) is released into culture medium upon plasma membrane damage and cell death. E.G7-Ova cells were pretreated with HDAC6 inhibitors, cocultured with B3Z cells at an E:T ratio of 2:1, and LDH in the culture media measured. Pretreatment of E.G7-Ova cells with HDAC6 inhibitors, followed by coculture with B3Z cells significantly increased LDH release (Fig. 2B). Killing of E.G7-Ova cells was also quantitating the annexin-V^+^/propidium iodide^+^ cells after coculture with B3Z cells by flow cytometry. Pretreatment of E.G7-Ova cells with tubastatin-A, ACY-738, and ACY-1215 significantly increased the number of CD8^neg^/annexin-V^+^/propidium iodide^+^ EG.7-Ova cells after coculture with B3Z cells (Fig. 2C). SGD-UBD253 is a chemical probe that potently and selectively inhibits the HDAC6-Ub binding domain (UBD; ref. 28). Treatment of E.G7-Ova cells with SGD-UBD253 increased the presentation of the SIINFEKL–MHC-I complex, consistent with the effect observed with the HDAC6 inhibitors (Fig. 2D and E). SGC-UBD253N is a structurally related, negative control for SGC-UBD253. Presentation of the SIINFEKL–MHC-I complex on E.G7-Ova cells was not changed after treatment with SGC-UBD253N (Supplementary Fig. S11A). Control experiments indicated that LDH released into the culture media of E.G7-Ova or B3Z cells incubated alone or E.G7-Ova cells incubated with proteasome activators in the absence of B3Z cells was low (Supplementary Fig. S12A and S12B). Pretreatment of B3Z cells with HDAC6 inhibitors followed by E.G7-Ova coculture also did not increase LDH levels to indicate that the proteasome activators acted on the E.G7-Ova cells and not B3Z cells. Control experiments also showed that treatment of E.G7-Ova and B3Z cells with the HDAC6 inhibitors did not induce apoptosis (Supplementary Fig. S12C and S12D). Treatment of E.G7-Ova cells with other HDAC6 inhibitors followed by coculture with B3Z cells also induced apoptosis (Supplementary Fig. S12E).

**FIGURE 3:**
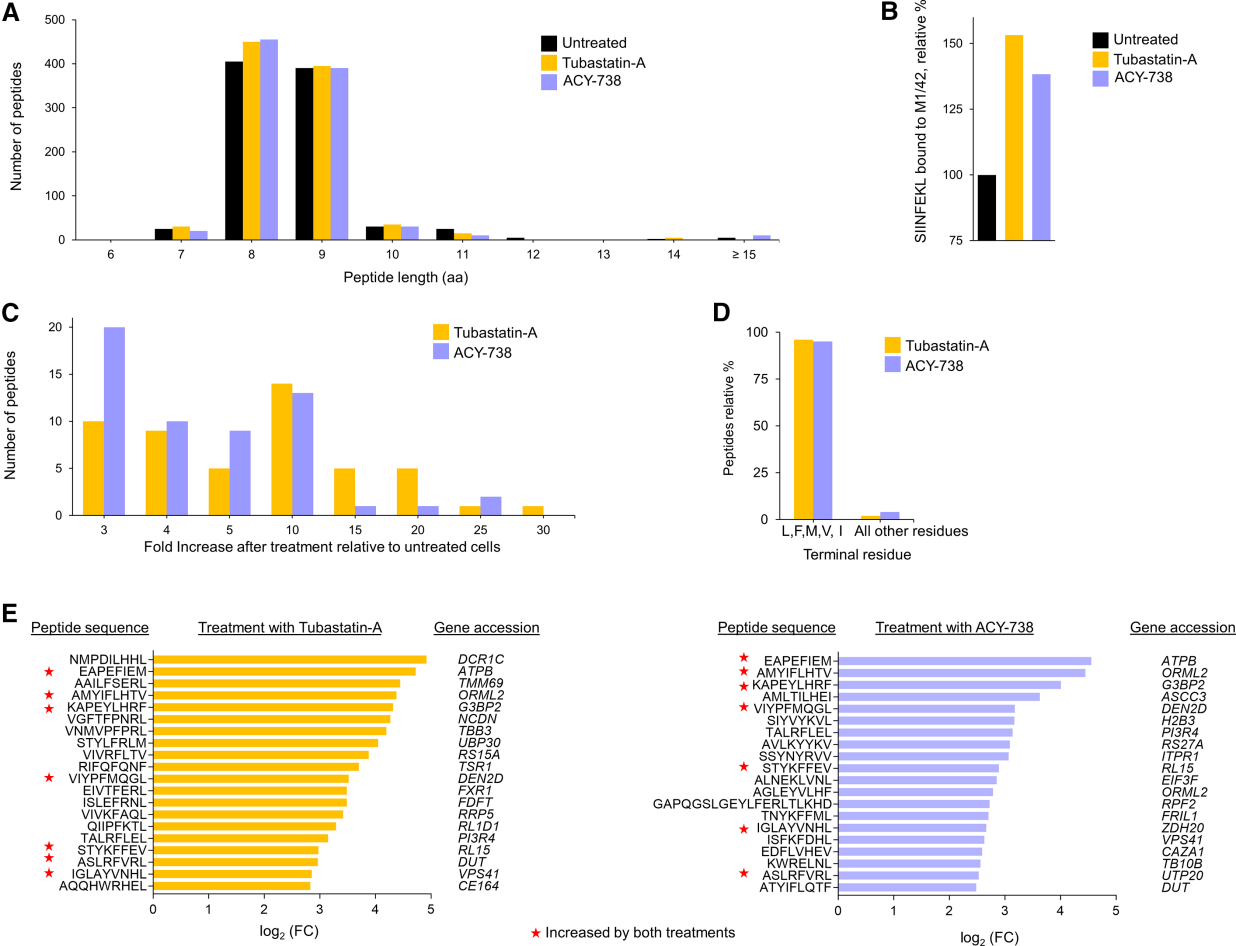
Effect of HDAC6 inhibitors on the murine MHC class I immunopeptidome. **A,** Number of peptides eluted from murine MHC class I molecule at each indicated peptide lengths (range = 6 to 15 amino acids). **B,** Effect of HDAC6 inhibitor treatment on SIINFEKL presentation. **C,** Number of peptides and their fold increase after treatment of E.G7-Ova cells with each HDAC6 inhibitor. **D,** The terminal amino acids of the top 50 peptides upregulated by tubastatin-A or ACY-738 treatment were analyzed. Shown is the relative percent for each terminal amino acid for the top 50 peptides upregulated by treatment with each HDAC6 inhibitor. **E,** Shown are gene accession numbers for the top 20 peptides most upregulated following treatment with either tubastatin-A or ACY-738. Red asterisks indicate genes and peptides that were upregulated following treatment with both of the HDAC6 inhibitors.

Effect of HDA6 inhibitors on the murine MHC-I immunopeptidome. Because treatment with HDAC6 inhibitors increased presentation of the SIINFEKL–MHC-I complex, we determined the more global effect of HDAC6 inhibitors on the murine class I immunopeptidome. E.G7-Ova cells were treated with tubastatin-A and ACY-738 at 1 µmol/L for 72 hours. Cell lysates were prepared and immunoprecipitated using an antibody (M1/42) to the murine pan-MHC-I molecule. Peptides were eluted from the class I molecules and sequenced by MS (29). A total of 1,771 peptides were detected at 1% PSM FDR (based on forward/decoy database searching). The length of eluted peptides was not altered by HDAC6 treatment (Fig. 3A; Supplementary Fig. S13). The peak area of SIINFEKL was 1.5-fold higher in samples treated with tubastatin-A or ACY-738 compared with untreated counterparts (Fig. 3B). Moreover, the peak area value for 107 peptides was increased ≥2-fold after treatment with HDAC6 inhibitors, while the peak area for some peptides was increased ≥25-fold (Fig. 3C). Treatment with the HDAC6 inhibitors also yielded an increase in peptides bearing a terminal aliphatic or aromatic residue, consistent with the experimentally observed increase in proteasome ChT-like activity (Fig. 3D). Analysis of the top 50 peptides most upregulated by HDAC6 inhibitor treatment indicated that the overwhelming majority (96%) terminated in either an aliphatic or aromatic residue (L, F, M, V, I). While immunoprecipitations were performed using a pan-MHC-I antibody, the eluted peptides were selectively increased for peptides that terminated in residues cleaved by the proteasome ChT-like active site. Genes were identified for the 20 peptides most upregulated by tubastatin-A or ACY-738 treatment (Fig. 3E). Notably, some peptides were increased 30-fold after treatment with the HDAC6 inhibitors. Seven of the top 20 most upregulated peptides were commonly upregulated by either tubastatin-A or ACY-738 treatment.

### Effect of HDAC6 Inhibitors on the Myeloma Immunopeptidome

**FIGURE 4:**
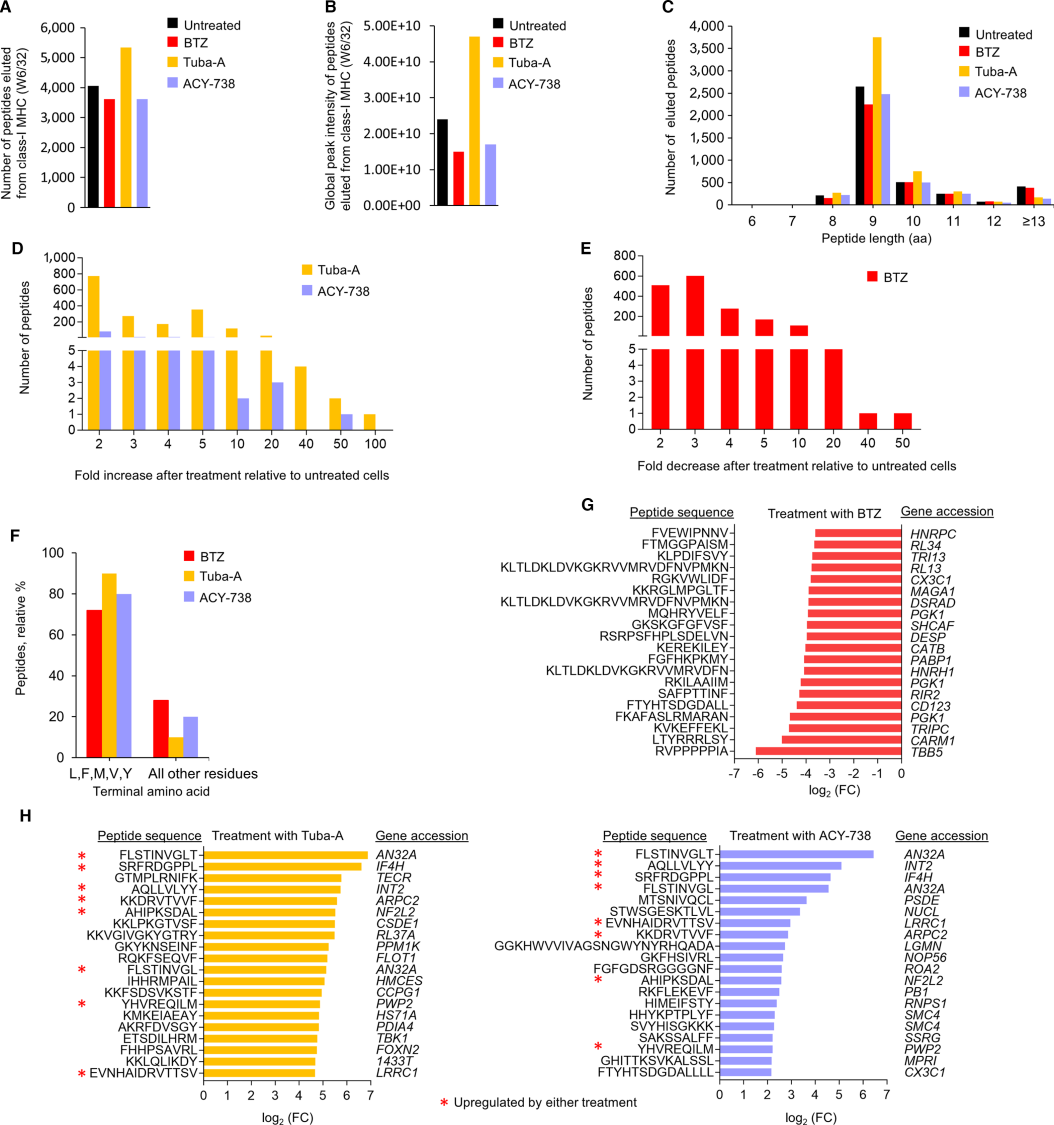
Effect of HDAC6 inhibitors on the myeloma immunopeptidome. **A,** Number of peptides eluted from human MHC-I molecules at each indicated peptide lengths (range = 6 to 15 amino acids). RPMI-8226 cells were treated with tubastatin-A, ACY-738 or bortezomib. **B,** Number of peptides and their fold change after treatment of RPMI-8226 cells with tubastatin-A, ACY-738, or bortezomib. **C,** The terminal amino acids of the top 50 peptides upregulated by tubastatin-A or ACY-738 treatment were analyzed. Shown is the relative percent of each terminal amino acid for the top 50 peptides upregulated by HDAC6 inhibitor treatment. **D,** Shown is the number of peptides reduced by treatment of E.G7-Ova cells with bortezomib and the fold decrease relative to untreated cells. **E,** Shown is the peptide sequence, fold decrease, and gene accession for the 20 peptides most downregulated by bortezomib treatment. Red asterisks indicate peptides upregulated by treatment with both HDAC6 inhibitors. **F,** The terminal amino acids of the top 50 peptides upregulated by tubastatin-A or ACY-738 treatment were analyzed. Shown is the relative percent of each terminal amino acid for the top 50 peptides upregulated following treatment with each HDAC6 inhibitor. **G,** Shown are gene accession numbers for the top 20 peptides most downregulated by bortezomib treatment. **H,** Shown are gene accession numbers for the top 20 peptides most upregulated by either tubastatin-A or ACY-738. Red asterisks indicate genes and peptides that were upregulated following treatment with both of the HDAC6 inhibitors.

Because treatment of E.G7-Ova cells with HDAC6 inhibitors modulated the murine MHC-I immunopeptidome, we next determined the effect of HDAC6 inhibitors on the myeloma class I immunopeptidome. The W6/32 mAb reacts with major HLA-ABC antigens that associate with β2-microglobulin and are expressed by human nucleated cells. RPMI-8226 cells were treated with either tubastatin-A or ACY-738, or bortezomib, lysates prepared, immunoprecipitated using the W6/32 antibody, bound peptides eluted and sequenced by MS. A total of 8,019 eluted peptides were detected at 1% PSM FDR (based on forward/decoy database searching; Fig. 4A and B). The global peak intensity of peptides was approximately 2-fold greater with tubastatin-A, while peak intensity was slightly decreased after bortezomib treatment (Fig. 4B). Average peptide length was not changed after treatment with bortezomib or the HDAC6 inhibitors (Fig. 4C). Tubastatin-A treatment elicited a ≥2-fold increase in 1,726 peptides and the relative intensity of 34 peptides was increased more than 20-fold. ACY-738 treatment elicited a ≥2-fold increase in 118 peptides and the intensity of four peptides was increased more than 20-fold. Bortezomib treatment resulted in a 2-fold or greater decrease in 1,707 peptides while seven peptides decreased by at least 20-fold (Fig. 4D). Treatment with the HDAC6 inhibitors also yielded an increase in peptides bearing a terminal aliphatic or aromatic residue, consistent with the experimentally observed increase in proteasome ChT-like activity (Fig. 4E). Analysis of the top 50 peptides most upregulated by the HDAC6 inhibitors indicated that the vast majority (85%) terminated in either an aliphatic or aromatic residue (L, F, M, V, Y). While immunoprecipitations were performed using a pan-MHC-I antibody (W6/32), the eluted peptides were selectively increased for those cleaved by proteasome ChT-like activity. Genes were identified for the 20 peptides most downregulated by bortezomib treatment or most upregulated by tubastatin-A or ACY-738 treatment (Fig. 4F and G). Interestingly, eight of the 20 most upregulated peptides were increased after treatment with either HDAC6 inhibitor.

Neoantigens are newly formed antigens generated by tumor cells because of genomic mutation, dysregulated RNA splicing, disordered posttranslational modification (PTM), and integrated viral open reading frames (30, 31). Neoantigens are recognized as non-self and trigger an immune response that is not subject to central and peripheral tolerance to represent an effective approach to induce, amplify and diversify antitumor T-cell responses. We identified 30 neoantigens on multiple myeloma cells, generated through either mutation or PTM, that were upregulated by treatment with tubastatin-A or ACY-738 (Supplementary Table S5A). Traditional TAAs are not unique to the tumor because they are also present in normal tissues but are highly expressed in proliferating tumor cells (31). We also identified eight TAAs upregulated by both tubastatin-A and ACY-738 treatment and 31 TAAs were upregulated by either agent (Supplementary Table S5B). Importantly, many TAAs that were upregulated by HDAC6 inhibitor treatment were downregulated by bortezomib treatment.

### HDAC6 Inhibition Releases HR23B to Increase Proteasome Activity

**FIGURE 5:**
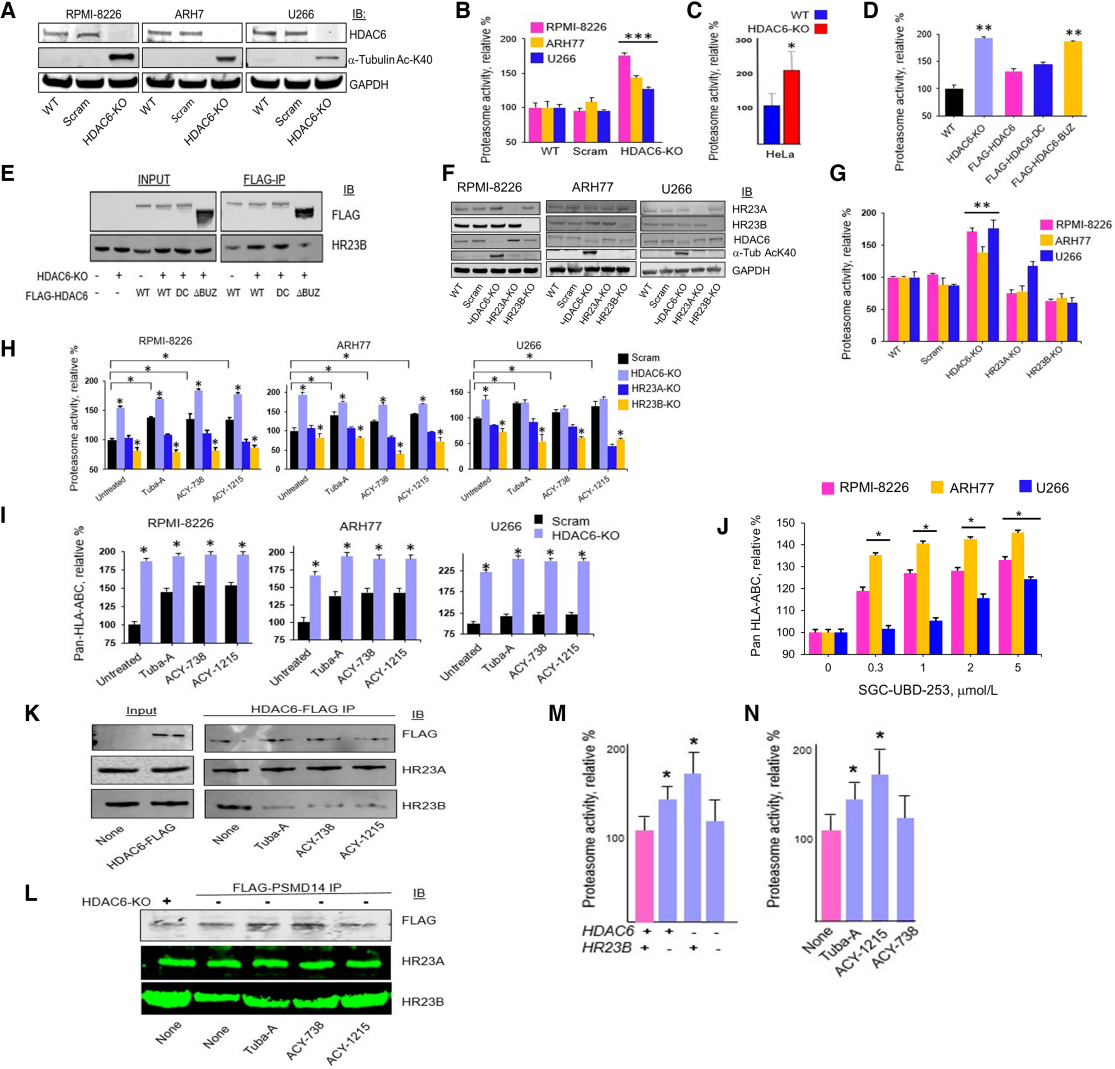
Effect of HDAC6 KO on proteasome activity and antigen presentation **A,** SDS-PAGE of lysates from HDAC6 KO in MMCLs RPMI-8226, U266, and ARH-77. **B,** Effect of sgRNA targeting *HDAC6* on proteasome ChT-like activity in MMCLs. **C,** Effect sgRNA targeting *HDAC6* WT and mutant forms on proteasome ChT-like activity in HeLa cells in which endogenous HDAC6 had been knocked out by sgRNA. **D,** Effect of HDAC6 WT, DC, and ΔBUZ mutants on proteasome ChT-like activity in HeLa cells. HeLa-HDAC6-KO cells were transfected with pcDNA-HDAC6-FLAG (RRID: Addgene_30482), pcDNA-HDAC6.DC-FLAG (RRID: Addgene_30483) and pcDNA-HDAC6.ΔBuz-FLAG (RRID: Addgene_30484), and 48 hours posttransfection, cells were incubated with the proteasome substrate LLVY-R110. Proteasome activity was determined by measuring R110 levels using a fluorometric plate reader. **E,** Effect of WT and mutant *HDAC6* on co-immunoprecipitation of HR23B. HeLa cells were transfected with sgRNA to target HDAC6 and then transfected with plasmids that expressed full-length, WT HDAC6 (pcDNA-HDAC6-FLAG), HDAC6 catalytic active site mutant (pcDNA-HDAC6.DC-FLAG) and HDAC6-BUZ domain mutant (pcDNA-HDAC6.ΔBuz-FLAG). Cells were harvested 48 hours after transfection, washed with PBS, lysates prepared under nondenaturing conditions and immunoprecipitated using the Pierce anti-DYKDDDDK affinity resin for 2 hours at 4°C followed with elution with the Pierce 3X DYKDDDDK peptide. Samples were separated by SDS-PAGE, transferred to PVDF and immunoblots probed with anti-FLAG or HR23B antibodies. Immunoblots of input FLAG and HR23B are shown. Deletion of the C-terminal BUZ domain eliminated co-immunoprecipitation of HR23B. Note that the enhanced levels of ΔBUZ compared with pcDNA-HDAC6-FLAG and pcDNA-HDAC6.DC-FLAG in immunoprecipitates reflects increased expression of input pcDNA-HDAC6.ΔBUZ-FLAG. **F,** Effect of sgRNA targeting *HR23A*, *HR23B*, and *HDAC6* on HR23B association with HDAC6 in RPMI-8226, U266, and ARH-77 cells. Multiple myeloma cells were transfected with scrambled (control) sgRNA or sgRNA to target *HDAC6*, *HR23A* or *HR23B*. Lysates were prepared and HDAC6 catalytic activity determined using α-tubulin Ac-Lys-40. HDAC6 deacetylates *α-tubulin* and pharmacologic or genetic inhibition of HDAC6 catalytic activity leads to an increase in α-tubulin acetylation. The effect of sgRNA targeting HDAC6 was confirmed by the increase in α-tubulin Ac-Lys-40. **G,** Effect of sgRNA targeting *HDAC6, HR23A,* and *HR23B* on proteasome ChT-like activity in MMCLs. Cells were electroporated with sgRNA as above. Proteasome ChT-like activity was determined using LLVY-R110. **H,** Effect of HDAC6 inhibitors on proteasome ChT-like activity in MMCLs. Cells were transfected with the indicated sgRNA, incubated for 72 hours and then treated with each HDAC6 inhibitor (1 µmol/L). Proteasome ChT-like activity was determined using LLVY-R110. **I,** Effect of HDAC6 inhibitors on pan-HLA-ABC presentation in MMCLs. Cells were transfected with the indicated sgRNA, incubated for 72 hours and then treated with each HDAC6 inhibitor (1 µmol/L). The effect of each sgRNA on pan-HLA-ABC expression on MMCLs was determined by flow cytometry. Shown is the relative change in pan-HLA-ABC expression. **J,** Effect of the HDAC6-BUZ domain inhibitor SG-UBD235 on pan-HLA ABC presentation in MMCLs. Cells were treated with SG-UBD253 at the indicated concentrations for 24 hours, washed, stained with a conjugated antibody to pan-HLA ABC and quantitated by flow cytometry. **K,** Effect of HDAC6 inhibitors on co-immunoprecipitation of HR23A and HR23B with FLAG-tagged HDAC6 in RPMI-8226 cells. Cells were transfected with a plasmid that expressed full-length, WT FLAG-tagged HDAC6, treated with HDA6 inhibitors (1 µmol/L) for 24 hours, lysates prepared and separated by SDS-PAGE. Membranes were then probed for the indicated proteins by immunoblot. **L,** Effect of HDAC6 inhibitors on co-immunoprecipitation of HR23A *and HR23B* with FLAG-tagged-PSMD14 in RPMI-8226 cells. All bioassays were performed in triplicate. Error bars represent the relative SEM. A *P*-value ≤ 0.05 is flagged with one star (*) and a *P*-value ≤ 0.01 is flagged with two stars (**). **M,** Effect of sgRNA targeting *HDAC6* and *HR23B* in RPMI8226 cells on proteasome ChT-like activity. Cells that had been transfected with sgRNA to HDAC6, HR23B or both were transfected with a plasmid that expressed FLAG-tagged PSMD14. Lysates were prepared under nondenaturing conditions, immunoprecipitated using FLAG-sepharose, eluted using 3X-FLAG peptide and proteasome activity assay buffer added (20 mmol/L Tris-HCl, pH 7.6, 20 mmol/L NaCl, 3 mmol/L ATP, 5 mmol/L MgCl_2_, 1 mmol/L DTT, 10% glycerol). Suc-LLVY-MCA was then added (100 µmol/L) and proteasome activity measured using a fluorometer at an excitation 360 nmol/L and emission 440 nmol/L. **N,** Effect of HDAC6 inhibitors on proteasome activity in RPMI-8226 cells. Cells were transfected with a plasmid that expressed FLAG-tagged PSMD14, lysates prepared, immunoprecipitated using FLAG-sepharose, eluted using FLAG peptide and proteasome assay buffer added (20 mmol/L Tris-HCl, pH 7.6, 20 mmol/L NaCl, 3 mmol/L ATP, 5 mmol/L MgCl_2_, 1 mmol/L DTT, 10% glycerol). Suc-LLVY-MCA was then added (100 µmol/L) and proteasome activity measured using a fluorometer at an excitation 360 nmol/L and emission 440 nmol/L. All assays were performed in triplicate. A *P*-value ≤ 0.05 is flagged with one star (*).

To investigate the mechanistic role of HDAC6 in modulating proteasome activity, *HDAC6* was targeted in three MMCLs by *CRISPR/Cas9* editing and target gene KO was confirmed by Western blot analysis (Fig. 5A). MMCLs were treated with HDAC6 inhibitors at the indicated concentrations and proteasome activity and cell viability determined (Fig. 5B and C; Supplementary Fig. S14A and S14B). Proteasome activity was increased nearly 2-fold in MMCLs after *HDAC6* KO relative to controls. Similar results were observed in HeLa cells following *HDAC6* KO (Fig. 5C). Results indicated that treatment with tubastatin-A, ACY-1215, and ACY-738 increased proteasome activity 50%–100%. At higher concentrations, ACY-1215 and ACY-738 reduced cell viability 15%–20%. The HDAC1/2 inhibitor romidepsin (FK228/depsipeptide), the pan-HDAC inhibitor vorinostat (suberoylanilide hydroxamic acid) and the HDAC1/3/6/8 inhibitor panobinostat (LBH589) did not significantly increase proteasome activity (Supplementary Fig. S14C). Dynamic acetylation equilibrium of cellular proteins is maintained primarily through the physical and functional interplay between HDAC activities and histone acetyltransferases (HAT) activators. HAT activators did not increase proteasome activity, suggesting alternative mechanisms of action (Supplementary Fig. S14D). In addition, treatment of multiple myeloma cells with bortezomib increased formation of both aggresomes and autophagosomes (Supplementary Fig. S15A and S15B). HDAC6 is required for the formation of aggresomes as well as the maturation of autophagosomes. However, co-treatment with the HDAC6 inhibitors suppressed the effects of bortezomib to suggest that HDAC6 inhibitors were not increasing antigen presentation through upregulated autophagy.

*HDAC6 KO* cells were then transfected with plasmids that expressed either FLAG-tagged WT HDAC6, a catalytic domain HDAC6 mutant (DC) or an HDCA6 mutant that lacked the C-terminal Ub-binding zinc (Zn^2+^; BUZ) domain (Fig. 5D). HR23B is a biomarker previously identified in a genome-wide loss-of-function screen which influenced sensitivity to HDAC inhibitors (32). HR23B belongs to the diverse protein family that contain an N-terminal UBL domain and prior studies have shown that certain UBL proteins bind and enhance proteasome activity (33–36). The UBL domain mediates HR23B binding to proteasomes, which in turn enhances the delivery of ubiquitinylated cargo and promotes protein degradation. HeLa lysates were immunoprecipitated using a FLAG antibody and probed for HR23B association with HDAC6. Results indicated that deletion of the *HDAC6-BUZ* domain reduced co-immunoprecipitation of HR23B with FLAG-HDAC6 (Fig. 5E). *HDAC6, HR23A,* and *HR23B* were knocked out in RPMI-8226, ARH77, and U266 cells using *CRISPR/CAS9* gene editing (Fig. 5F). *HDAC6 KO* in each MMCL led to increased proteasome ChT-like activity relative to cells transfected with control sgRNA (Fig. 5G). *CRISPR/CAS9* KO of *HR23A* or *HR23B* in MMCLs did not increase proteasome activity (Fig. 5H). The same multiple myeloma cells were then treated with tubastatin-A, ACY-1215 or ACY-738 and proteasome activity measured. Results indicated that while proteasome activity was increased in *HDAC6 KO* cells relative to control transfected cells, *HR23B* KO eliminated the effect of *HDAC6* KO and HDAC6 inhibitors on proteasome activity (Fig. 5H). Importantly, proteasome ChT-like activity in *HR23B* KO cells transfected with sgRNA to was more evident than the effect of *HR23A* inactivation.

We next determined the effect of HDAC6 on pan-HLA expression on multiple myeloma cells because we had shown that HDAC6 inhibitors and *HDAC6* KO increased proteasome activity. *HDAC6* KO in the MMCLs tested significantly increased the detection of pan HLA-A, B, C expression on multiple myeloma cells by flow cytometry. In addition, the effect of the HDAC6 inhibitors on pan-HLA expression was only slightly increased in HDAC6 KO cells (Fig. 5I). SGD-UBD253 is a SGD-UBD253 treatment of MMCLs also increased pan-MHC-I presentation in a dose-dependent manner (Fig. 5J). Treatment of multiple myeloma cells with the negative control SGD-UBD253N MMCLs did not increase pan-MHC-I presentation (Supplementary Fig. S11B).

Cells were then transfected to express FLAG-HDAC6 and treated with each HDAC6 inhibitor. Lysates were prepared under nondenaturing conditions, immunoprecipitated with a FLAG-specific antibody and probed with antibodies specific to HR23A and HR23B (Fig. 5K). The results indicated that the HDAC6 inhibitors reduced the association of HR23B, but not HR23A, with HDAC6. The results suggest that treatment with HDAC6 inhibitors preferentially releases HR23B from HDAC6 compared with HR23A. Similarly, RPMI-8226 cells were transfected to express FLAG-tagged PSMD14, treated with HDAC6 inhibitors, immunoprecipitated using a FLAG-specific antibody and the immunoprecipitates probed with antibodies specific to HR23A and HR23B. PSMD14 is an essential, non-ATPase subunit of the assembled 19S RP and component of 26S proteasomes. Treatment of RPMI-8226 cells with the HDAC6 inhibitors increased FLAG-PSMD14 association with HR23B but not HR23A (Fig. 5L).

To determine the direct effect of HDAC6 and HR23B on proteasome activity, RPMI-8226 cells were transfected with a plasmid that expressed FLAG-tagged PSMD14 which is an essential non-ATPase component of 26S proteasomes. FLAG-tagged PSMD14 was transfected into *HDAC6* and *HR23B* KO cells. Lysates were then immunoprecipitated using a FLAG-specific antibody, eluates resuspended in proteasome assay buffer and activity measured. Results showed that *HDAC6* KO increased proteasome activity but was dependent on *HR23B* expression (Fig. 5M). RPMI-8226 cells that expressed WT *HDCA6* and *HR23B* were also transfected with PSMD14 and treated with HDAC6 inhibitors. Proteasome activity was increased in the FLAG-PSMD14 immunoprecipitates that had been treated with HDAC6 inhibitors relative to controls (Fig. 5N).

### Effect of HDAC6 Inhibitors on Autologous T-cell Antimyeloma Activity

**FIGURE 6:**
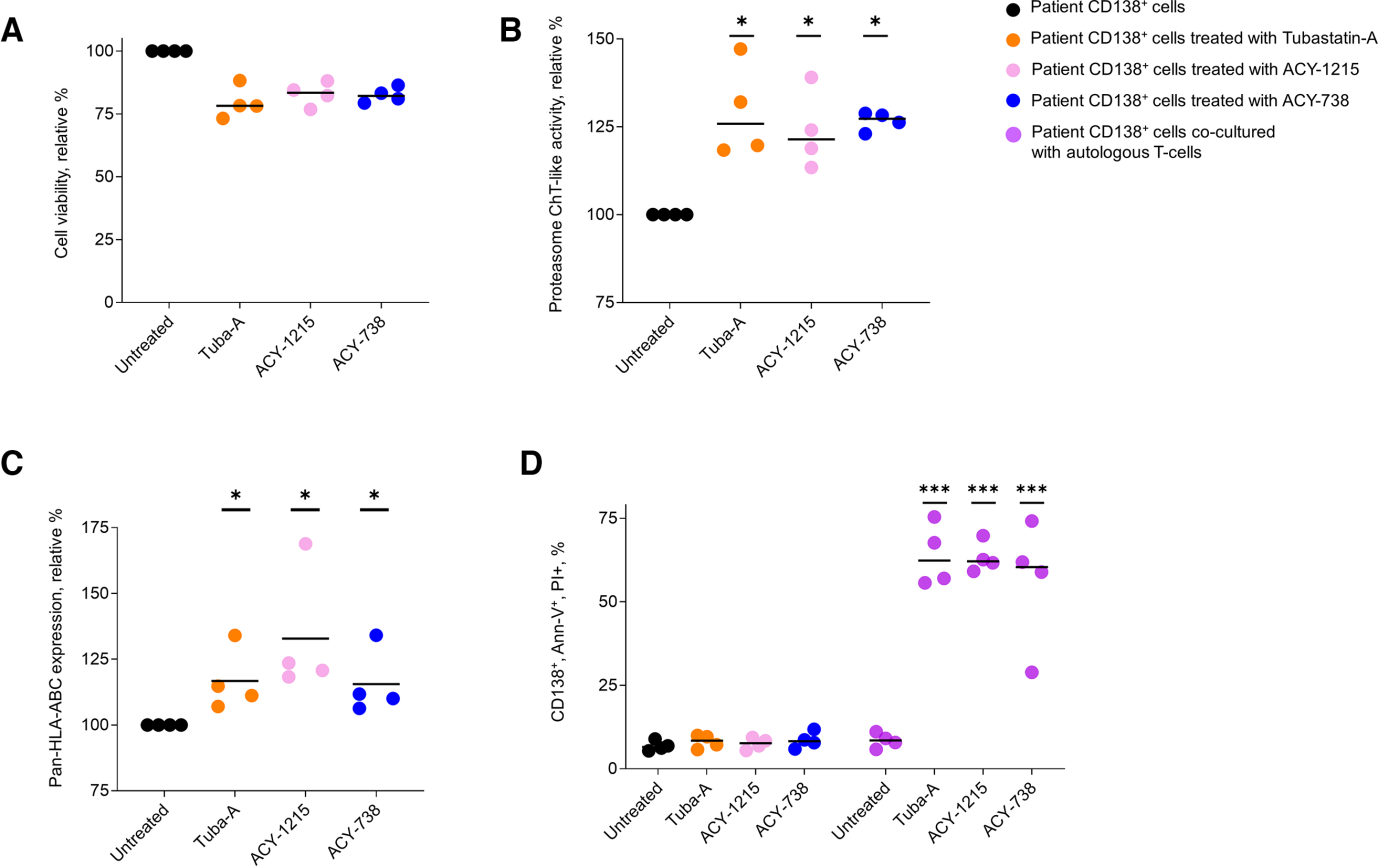
Effect of HDAC6 inhibitors on the antimyeloma effect of autologous T cells. **A,** Effect of HDAC6 inhibitors on the viability of myeloma patient CD138^+^ cells. Cells were treated with the pharmocologics at 1 µmol/L for 18 hours and cell viability measured using the XTT assay. Assays were performed in triplicate. **B,** Effect of HDAC6 inhibitors on ChT-like proteasome activity in CD138^+^ myeloma patient cells. Cells were treated with tubastatin-A, ACY-738, and ACY-1215 for 18 hours. Posttreatment cells were then incubated for 21 hours with LLVY-R110. Cleavage of LLVY-R110 was determined as above. **C,** Effect of HDAC6 inhibitors on pan MHC-I (HLA-ABC) presentation on CD138^+^ cells. Cells were treated with tubastatin-A, ACY-738 and ACY-1215 for 18 hours at 1 µmol/L, PBS washed, and stained with a mouse anti-human HLA-ABC (BD Pharmingen, 55555) and CD138 antibody per manufacturer's guidelines. Cells that stained positively with an anti-human HLA-ABC were analyzed on Attune Nxt and quantified using Flow Jo version 10.9.0. Error bars represent the median absolute deviation percentile (MADP) of the average. **D,** Effect of HDAC6 inhibitors on autologous T cell–mediated myeloma cytotoxicity. CD138^+^ cells were treated with tubastatin-A, ACY-738, and ACY-1215 for 18 hours and cocultured with T cells for 18 hours. After treatment, cells were then PBS washed and stained with CD138, FITC annexin-V in buffer containing propidium iodide for 20 minutes at room temperature. Cells were then analyzed on an Attune NxT and quantified using Flow Jo version 10.9.0. Error bars represent the MADP of the average. A *P*-value ≤ 0.05 is flagged with one star (*) and a *P*-value ≤ 0.001 is flagged with three stars (***).

The effect of HDAC6 inhibitors on cell viability, proteasome activity, and pan-MHC-I antigen presentation was determined using CD138^+^ cells isolated from the bone marrow of patients with multiple myeloma with treatment-refractory disease. After treatment with HDAC6 inhibitors, the viability of the CD138^+^ cells was reduced approximately 10%–20%, while proteasome activity was increased approximately 50% (Fig. 6A and B). Treatment of patient CD138^+^ cells with HDAC6 inhibitors increased MHC-I molecules by 20%–75% (Fig. 6C). Patient CD138^+^ cells were then treated with each HDAC6 inhibitor and cocultured with autologous patient CD8^+^ T-cells (E:T 2.5:1). The relative percentage of CD138^+^/annexin-V^+^/propidium iodide^+^ cells was significantly increased after pretreatment with the HDAC6 inhibitors followed by T-cell coculture (Fig. 6D). Treatment of patient CD138^+^ cells with HDAC6 inhibitors alone or T cells alone did not increase the percentage of annexin-V^+^/propidium iodide^+^ cells.

The results indicated that increased antigen presentation and CTL activity was demonstrated for the single ovalbumin epitope SIINFEKL that is recognized by SIINFEKL-restricted TCRs engineered in B3Z cells. To determine whether the effect was specific to SIINFEKL or more broadly applicable, multiple myeloma cells with an anti-HLA-ABC antibody and then cocultured with autologous T cells. Pretreatment of the multiple myeloma cells with the anti-HLA-ABC antibody significantly blocked the antimyeloma effect of autologous T cells (Supplementary Fig. S16).

A drawback of *in vitro* assays is the failure to capture the inherent complexity of organ systems. Patient-derived, three-dimensional (3-D) model systems may better reflect tumor cell complexity and heterogeneity compared with their counterparts that are cultured conventionally. Therefore, we addressed the effect of HDAC6 inhibitors on GFP-expressing multiple myeloma CD138^+^ cells that were seeded in a 3-D matrigel system to develop large spheroids. Multiple myeloma cells were seeded on matrigel, spheroids allowed to form and the spheroids were then treated with either tubastatin-A, ACY-1215, or ACY738. The spheroids were then treated with autologous T cells and GFP-expressing cells imaged. Cells were imaged using an IncuCyte SX5 instrument (Sartorius) with green fluorescence from myeloma spheroids quantitated at 8 hour intervals (Supplementary Fig. S17). Results showed that treatment with the HDAC6 inhibitors followed by T-cell addition reduced the level of fluorescence indicative of reduced spheroid expansion. Future studies will address the *in vivo* effect of HDAC6 inhibitors on myeloma cell antigen presentation and viability.

### Pan-cancer Effect of HDAC6 Inhibitors on Proteasome Activity and MHC-I Antigens

**FIGURE 7:**
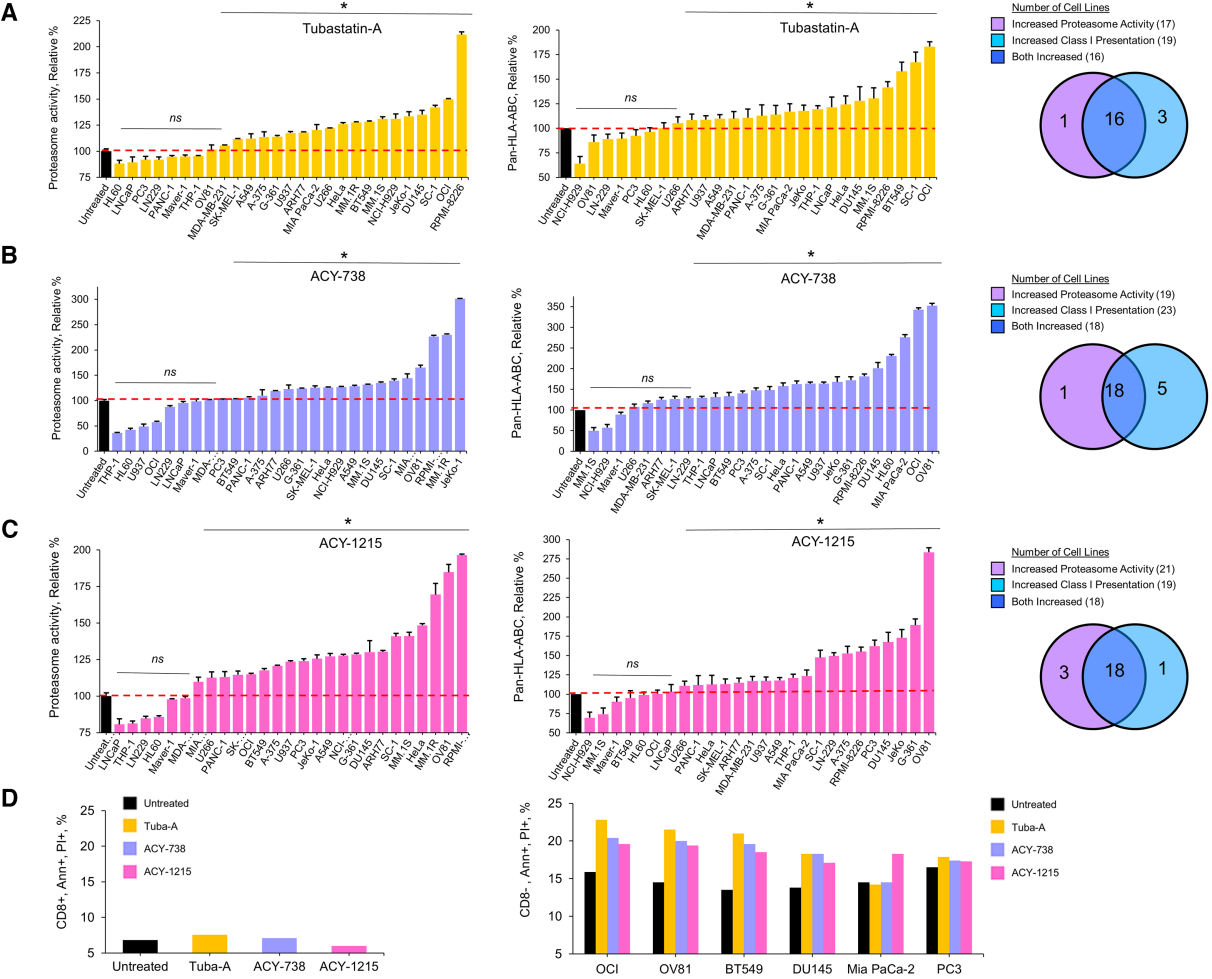
Pan-cancer cell effect of HDAC6 inhibitors on proteasomes and MHC class I antigen presentation. **A,** Effect of HDAC6 inhibitors on proteasome activity in cancer cell lines. Cells were treated with tubastatin-A, ACY-738 and ACY-1215 at 1 µmol/L concentration for 72 hours. Posttreatment cells were incubated for 21 hours with proteasome substrate LLVY-R110 and substrate cleavage was monitored as above. Cell lines were ranked on the basis of their effect on proteasome activity. **B,** Effect of HDAC6 inhibitors on pan MHC-I (HLA-ABC) in a panel of cancer cell lines. Cells were treated with tubastatin-A, ACY-738, and ACY-1215 for 72 hours at 1 µmol/L final concentration. Posttreatment the cells were washed with PBS and stained with mouse anti-human HLA-ABC (BD Pharmingen, 55555) as per manufacturer's guidelines. Cells that stained positively with an anti-human HLA-ABC were analyzed on Attune Nxt and quantified using Flow Jo version 10.9.0. The cells are rank ordered according to their MHC-I presentation. Error bars represent MADP of the average. **C,** Effect of HDAC6 inhibitors on healthy human T cells. CD8^+^ T cells were cultured for 18 hours with HDAC6 inhibitors, PBS washed, stained, and analyzed by flow cytometry to quantitate annexin-V^+^/propidium iodide^+^ cells. Shown is the average of triplicate measurements. **D,** Effect of HDAC6 inhibitors on CD8^+^ T-cell cytotoxicity of tumor cells. OCI-AML3 (leukemia), OV81 (ovarian), BT549 (breast), DU145 (prostate), MiaPaCa-2 (pancreatic), and PC3 (prostate) cells (1 × 10^6^ cells/treatment) were treated with tubastatin-A, ACY-738, or ACY-1215 for 48 hours (1 µmol/L). Cells were washed and cultured alone or with healthy human T cells (E:T 2.5:1) for 24 hours. Cells were PBS washed, stained, and analyzed by flow cytometry to quantitate CD8^neg^/annexin-V^+^/propidium iodide^+^ cells. Shown is the average of triplicate measurements. A *P*-value ≤ 0.05 is flagged with one star (*) and *ns* indicates a difference that was not significant.

We determined the effect of the HDAC6 inhibitors using 27 cancer cell lines representative of a diverse set of hematologic cancers and solid tumors. The HDAC6 inhibitors increased proteasome activity and pan-MHC-I expression in most cancer cell lines (Fig. 7A and B). The effect of each HDAC6 inhibitor on proteasomes also correlated with the effect on MHC class I expression (Supplementary Fig. S18A–S18C). Treatment of either CD8^+^ T cells or cancer cells with the HDAC6 inhibitors alone did not significantly increase the generation of annexin-V^+^/propidium iodide^+^ cells (Fig. 7C). In contrast, pretreatment of solid tumor cells with the HDAC6 inhibitors followed by T-cell coculture significantly increased the percentage of CD8^−^/annexin-V^+^/propidium iodide^+^ cells (Fig. 7D).

## Discussion

We have shown that HDAC6 inhibitors stimulate proteasomes to amplify and expand MHC-I antigen presentation on myeloma cells and promote cytotoxic T-cell responses. We hypothesized that proteasome activators represented a feasible approach to increase antigen presentation to enhance antitumor immunity. Using a MS-based immunopeptidomics approach, we characterized the effect of HDAC6 inhibitors on the antigenic landscape of multiple myeloma cells and identified canonical antigens and neoantigens upregulated by proteasome activation. Our results demonstrate that pharmacologic modulation of proteasomes alters the diversity and repertoire of antigen presentation, which lies at the gateway to adaptive immunity. Difficulties in identifying tumor-specific peptides that are presented by MHC-I molecules and the ability of tumor cells to impair antigen presentation as they evolve under T-cell selection limit the success immunotherapies. Our findings offer solutions to both problems because proteasome activation amplifies neoantigen and tumor-specific antigen presentation as actionable targets for endogenous and engineered T cells. Our results indicate that proteasome activation increases both private neoantigens unique to individual patients’ tumors as well as public neoantigens derived from recurrent mutations in cancer drivers. Increased presentation of a single antigenic peptide (SIINFEKL) promoted the cytotoxic activity of SIINFEKL TCR-restricted B3Z T cells. Together, our studies strongly suggest that proteasomes not only govern MHC-I antigen density but also dictate the potency of cytotoxic T-cell responses. Proteasome activation represents a keystone event that is essential for effective immune activation with potential therapeutic benefit for many types of cancer.

Prior studies identified two categories of small-molecule proteasome stimulators: gate-openers that expand substrate entry into the 20S CP inner chamber, for example, ursolic acid, chlorpromazine derivatives, MK-866, AM-404, vitamin E succinate; and allosteric stimulators which impact substrate hydrolysis by proteasomes, for example, betulinic acid, cytisine derivatives, and the imidazoline TCH165 (19–22). The phosphodiesterase type 4 inhibitor rolipram and other cAMP protein kinase activators raise proteasome activity, presumably through phosphorylation of Rpt6. Interestingly, we did not observe a direct effect of the HDAC6 inhibitors, abiraterone, DC661, riluzole, or salinomycin on the activity of proteasomes purified by conventional methods. Our results highlight the complexity of processes that regulate proteasome activity and the immense potential of therapeutics that exploit proteasome activity to treat human disease (Supplementary Fig. S19).

HDACs regulate the activity of tumor-suppressor genes and oncogenes that play crucial roles in tumorigenesis as well as in antigen presentation and therefore have been studied as therapeutic targets in solid tumors and hematologic malignancies, including multiple myeloma (37, 38). Nonselective pan-HDAC inhibitors, for example, and specific class I HDAC inhibitors, fro example, romidepsin, have shown significant anti-multiple myeloma activity in preclinical models (38). HDAC6 is a member of the class IIb family of HDAC enzymes that regulate PD-L1 expression in melanoma, osteosarcoma, and chronic lymphocytic leukemia (39–41). Genetic knockdown and pharmacologic inhibition by the HDAC6 inhibitors markedly reduced melanoma tumor growth and combination therapy further reduced tumor growth compared with the individual effect of either agent and led to a greater infiltration of activated cytotoxic T cells (42). ACY-1215 is the first isoform-selective, orally-available HDAC inhibitor in clinical trials with antimyeloma efficacy in combination with proteasome inhibitors (43). ACY-1215 exhibited an immunomodulatory effect by downregulating PD-L1 in non–small cell lung cancer and melanoma cells (44, 45). HDAC6 inhibitors have also been shown to synergize with immunomodulatory drugs as antimyeloma therapy (46).

The advancement of proteasome inhibitors from basic science studies to clinical trials demonstrates how a sophisticated appreciation of potential targets can expand the spectrum of novel agents available to treat difficult cancers and transform disease management (47). Pharmacologic blockade of proteasomes has revolutionized multiple myeloma treatment, a malignancy characterized by transformed plasma cells that serve as professional antibody-secreting factories and display exquisite sensitivity to disruptions in proteostasis (48). Importantly, proteasome inhibitors are administered to newly diagnosed, relapsed and refractory patients as upfront, salvage, and maintenance therapies. Because proteasome inhibitors reduce MHC-I antigen presentation, these agents may actually counteract the efficacy of endogenous T cells and rapidly emerging engineered, adoptive T-cell therapies.

UBLs are involved in an extraordinary number of biological processes through the modification of substrate proteins and deregulation of UBL modifications is associated with many diseases, specifically cancer (49). The reversible conjugation of UBLs to cellular proteins is among the most prevalent PTM, which modulates various cellular and physiologic processes by altering the activity, stability, localization, trafficking, or interaction networks of its target molecules (50). The fundamental role of the UPS and UBL conjugation pathways in normal cell function and in the disease state has prompted the search for small molecules that selectively disrupt or accentuate function. Despite a limited understanding of the molecular mechanisms of pathway targets, the regulation of UBL modifications is an attractive, and increasingly tractable, approach to targeting aberrant signaling pathways in multiple cancers and other diseases (51).

Our immunopeptidomic results reveal the massive pool of intracellular proteins that can be targeted for cancer therapies, for example, TCR-restricted T cells, vaccines, upon proteasome activation. In contrast, the chimeric antigen receptor (CAR) T-cell approach is generally limited to targeting membrane-bound proteins. As the quest for effective antimyeloma agents persists, the exploration of tumor-specific intracellular proteins as potential targets for MHC-I presentation represents an intriguing frontier. Only approximately 27% of cellular proteins are classified as membrane-associated, while the majority, approximately 73%, are intracellular. Underutilization of this abundant pool of intracellular proteins in the context of TCR–MHC-I interactions offers an untapped resource that holds the promise to generate potent antitumor responses. While CAR T-cells are based on T-cell activation downstream of TCRs and contain their own intracellular signaling domains, endogenous and TCR-engineered T-cells are MHC-restricted and require costimulatory molecules for activation and proliferation (52). This enables TCR-engineered T cells to recognize both surface and intracellular antigens through MHC-I antigen presentation as a significant advantage over CAR T-cells (53). Because MHC-I downregulation is an important mechanism of immune escape and acquired checkpoint inhibitor resistance, a novel gain-of-function strategy that upregulates the antigen presentation machinery and MHC-I expression may restore antitumor cellular immunity for clinical benefit in multiple myeloma and other cancers.

## Supplementary Material

Table S1 Table S1. Top activators of proteasomal ChT-Like activity. Shown are the top pharmacologics that increased proteasome ChT-like in the HTS.

Table S2 Table S2. Top inhibitors of proteasomal ChT-like activity. Shown are the top pharmacologics that decreased proteasome ChT-like in the HTS.

Table S3 Table S3. Target specificity of HDA6 inhibitors. Compounds were tested in 10-dose IC50 mode, in triplicate, with 3-fold serial dilution starting at 1 uM. The HDAC reference compound Trichostatin A (TSA) was tested in a 10-dose IC50 with 3-fold serial dilution starting at 10 uM. HDAC6 reference compound TMP269 was tested in a 10-dose IC50 with 3-fold serial dilution starting at 10 uM. HDAC10 reference compound Quinostat was tested in a 10-dose IC50 with 3-fold serial dilution starting at 10 uM. SIRT reference compound Nicotinamide was tested in a 10-dose IC50 with 3-fold serial dilution starting at 10 uM. SIRT reference compound Suramin was tested in a 10-dose IC50 with 3-fold serial dilution starting at 100 uM. Substrates used for HDAC 1, 2, 3, and 6 were fluorogenic peptide p53 (residues 379-382) RHKK(Ac)-AMC; HDAC 4, 5, 7, 9, 11 were fluorogenic HDAC classic 2a substrate trifluoroacetyl lysine; HDAC8 was fluorogenic peptide p53 residues 379-382 and NAD; HDAC10 was Ac- spermidine-AMC; SIRTs 1, 2, and 3- fluorogenic peptide p53 residues 379-382 and NAD; SIRT5- fluorogenic peptide Ac-Lys-succ and NAD. Curve fits were performed where enzyme activities at the highest compound concentration were <65%. No inhibition indicates that compound activity was negligible and could not be fit to an IC50 curve. IC50 value higher than 1.00E-06 M was estimated based on the best curve fitting available. Assays were determined in collaboration with Reaction Biology (Malvern, PA).

Table S4 Table S4. Proteasomal Fluorogenic Peptide Substrates. Shown are the fluorogenic substrates, catalytic site preferences and excitation and emission values used for detection of substrate hydrolysis.

Table S5 Table S5. NeoAgs regulated by proteasome activation or inhibition. Shown is the peptide sequence, gene accession number, mutation or PTM fold change in that peptide antigen following treatment with either tubastatin-A, ACY-738, or bortezomib. Green indicates upregulation and red indicates downregulation. Numbers indicate the fold-change relative to untreated RPMI-8226 cells. Arrows indicate the direction of change for peptide sequences that were not detected in untreated cells. ND (blue) indicates that the peptide was not detected under the indicated condition. Blue asterisks indicate that the antigen was not detected in untreated cells and unmasked after treatment with the HDAC6 inhibitor.

Table S6 Table S6. TAAgs regulated by proteasome activation or inhibition. Shown is the peptide sequence, gene accession number, mutation or PTM fold change in that peptide antigen following treatment with either tubastatin-A, ACY-738, or bortezomib. Green indicates upregulation and red indicates downregulation. Numbers indicate the fold-change relative to untreated RPMI-8226 cells. Arrows indicate direction of change for peptide sequences that were not detected in untreated cells. ND (blue) indicates that the peptide was not detected under the indicated condition. Blue asterisks indicate that the antigen was not detected in untreated cells and unmasked after treatment with the HDAC6 inhibitor.

Figure S1 Fig. S1. Conversion of the constitutive proteasome to immunoproteasome by IFN-γ-induced subunit substitution.

Figure S2 Fig. S2. a. Correlation of proteasome ChT-like activity with cell number. Proteasome ChT-like activity was determined with indicated number of RPMI-8226 cells at 24 hrs. b. Correlation of proteasome ChT-like activity with incubation time. Proteasome ChT-like activity was determined with 50,000 RPMI-8226 cells/well at indicated time points. c. Correlation of proteasome ChT-like activity with LLVY-R110 concentration. Proteasome ChT-like activity was determined with 50,000 RPMI-8226 cells/well after 24 h of incubation. d. Effect of DMSO (%) on proteasome activity in three MMCLs.

Figure S3 Fig. S3. Chemical structure of top pharmacologics identified in the HTS that increased proteasome ChT-like activity.

Figure S4 Fig. S4. a. Effect of hits from the HTS on proteasome ChT-like activity on ARH-77 and U266 MMCLs. Proteasome ChT-like activity was determined with 50,000 ARHG-77 or U266 cells/well after 24 h of incubation with 50 uM LLVY-R110. b. Effect of proteasome inhibitors on proteasome ChT-like activity in three different MMCLs. Bortezomib, carfilzomib and ixazomib were added to MM cells at indicated concentrations. Proteasome ChT-like activity was then determined after 24 h incubation.

Figure S5 Fig. S5. Effect of top pharmacologics on a. inhibition of proteasome ChT-like activity in MM cells and b. MM cell viability.

Figure S6 Fig. S6. Dose-dependent effect of ACY-1215 and tubastatin-A on the biochemical activity of the HDACs and SIRTs.

Figure S7 Fig. S7. E.G7-Ova system to quantitate the SIINFEKL-MHC class I complex.

Figure S8 Fig. S8. Effect of top pharmacologics from the HTS that inhibited proteasome ChT-like activity in E.G7-Ova and EL4 cells. Cells (50,000/well) were incubated with each pharmacologic for 72 hrs. Proteasome ChT-like activity was determined after 24 hrs incubation. E.G7-Ova and EL4 cells. Cells (50,000/well) were incubated with each pharmacologic for 72 h. Proteasome ChT-like activity was determined after 24 h incubation.

Figure S9 Fig. S9. Effect of the top pharmacologics that increased proteasome ChT-like activity on presentation of the SIINFEKL-MHC class I molecule complex. E.G7-Ova cells were treated with pharmacologics at 3 uM for 72 h. Cells were then stained with a monoclonal antibody to Ova 257-264 (SIINFEKL) peptide bound to H2Kb and quantitated using a BD-LSRII sorter interfaced with FlowJo software.

Figure S10 Fig. S10. Effect of proteasome inhibitors on presentation of the SIINFEKL-MHC class I molecule complex. E.G7-Ova cells were treated with pharmacologics at the indicated concentration for 16 h. Cells were then stained with a monoclonal antibody to Ova 257-264 (SIINFEKL) peptide bound to H2Kb and quantitated using a BD-LSRII sorter interfaced with FlowJo software as above.

Figure S11 Fig. S11. Effect of the SGC-UBD253N (catalog number SML-3542, Sigma-Aldrich/Millipore-Sigma, Burlington, MA) is a negative control) probe on SIINFEKL-H2Kb presentation on E.G7-Ova cells (a) and effect of SGC-UBD253N on pan HLA-ABC presentation on three different MM cells (b). SGC-UBD253N is a closely related negative control for SGC-UBD253, a chemical probe for the HDAC6 UBD. E.G7-Ova (a) and MM cells (b) were treated with SGC-UBD253N at indicated concentration for 72h and the relative cell surface of the SIINFEKL-H2Kb complex detected by flow cytometry. Cells were then stained with a monoclonal antibody to SIINFEKL-H2Kb and quantitated using a BD-LSRII sorter interfaced with FlowJo software as above. MMCLs were treated with SGC-UBD253N at the indicated concentrations for 72 h. Cells were then stained with an anti- HLA-ABC (W6/32) antibody and quantitated using a BD-LSRII sorter interfaced with FlowJo software as above. Values represent the average of triplicate measurements. Error bars represent the SD.

Figure S12 Fig. S12. a. Effect of the top pharmacologics alone on E.G7-Ova cell viability as measured by LDH release into the culture medium (green). Also shown is the effect of pre-treating the B3Z cells with the pharmacologics then co-culturing with E.G7-Ova cells (red). b. Effect of pre- treatment of B3Z cells with pharmacologics followed by co-culture with E.G7-Ova cells. Cell viability was measured by annexin-V+ staining as above. c. Effect of HDAC6 inhibitors on E.G7-Ova viability. d. Effect of HDAC6 inhibitors on B3Z viability. e. Effect of HDAC6 inhibitors on E.G7-Ova viability after co-culture with B3Z cells at E:T 2:1.

Figure 13 Fig. S13. MS workflow used to identify MHC class I peptides increased by treatment with HDAC6 inhibitors or bortezomib.

Figure S14 Fig. S14. Effect of HDAC6 inhibitors at the indicated concentrations and times on a. proteasome activity and b. cell viability. Panel c. shows the effect of non-specific HDAC inhibitors on proteasome activity and cell viability. In panels a, b, and c, MM cells (50,000/well) were incubated with each pharmacologic at 1 uM for 72 h. Cell viability was determined using the XTT assay. d. Effect of HAT activators on proteasome activity and cell viability. MM cells (50,000/well) were incubated with each pharmacologic at 1 uM for 72 h. Cell viability was determined using the XTT assay.

Figure S15 Fig. S15. Effect of HDAC6 inhibitors on a. aggresome formation and b. autophagosome formation. RPMI8226 cells were treated with bortezomib and HDAC6 at the indicated concentrations for 16 h. Aggresomes were quantitated by flow cytometry using the cell-based Proteostat® aggresome detection kit (Enzo Life Sciences, Farmingdale, NY). The kit utilizes a molecular rotor dye which while in solution is prevented from fluorescing by free intramolecular rotation along a single central bond. Specifically intercalation of the dye into the cross-β spine of quaternary protein structures typically found in misfolded and aggregated proteins, inhibits the dye’s rotation and leads to a strong fluorescence. b. Autophagosome formation. RPMI8226 cells were treated with bortezomib and HDAC6 at the indicated concentrations for 16 h. Autophagosomes were quantitated by flow cytometry using the Cyto-ID autophagosome detection kit which measures autophagic vacuoles and monitors autophagic flux in lysosomally inhibited live cells using a novel dye that selectively labels accumulated autophagic vacuoles (Enzo Life Sciences). The 488nm-excitable green dye allows for minimal staining of lysosomes while exhibiting bright fluorescence upon incorporation into pre-autophagosomes, autophagosomes, and autolysosomes (autophagolysosomes). All assays were performed in triplicate. Error bars represent the SD of the mean.

Figure S16 Fig. S16. Effect of anti-HLA antibody on the generation of apoptotic MM cells after treatment with HDCA6 inhibitors and co-culture with autologous T-cells. MM patient CD138+ cells (20,000/ sample) were treated with each HDAC6 inhibitor (1 uM) for 24 h followed by incubated with 0.5 ug/mL mouse anti-HLA ABC antibody W6/32 (catalog number 14-9983-82, Fisher Scientific, Pittsburgh, PA) followed by co-culture with T-cells (E:T 2:1). The percent of apoptotic cells was quantitated by flow cytometry. Values represent the average of triplicate measurements. Error bars represent the SD of the mean.

Figure S17 Fig. S17. Effect of HDAC6 inhibitors on the expansion of MM spheroids cultured alone or with T-cells. MM patient CD138+ cells (50,000/ sample) were cultured in 50 μL of Matrigel (Corning) in 96-well plates according to the manufacturer’s protocol. Spheroids were allowed to form and maintained at 37 °C. Cells were then treated with HDAC6 inhibitors (1 uM) for 24 h followed by co-culture with T-cells (E:T 2;1) for another 24 h. Values represent the average of triplicate measurements.

Figure S18 Fig. S18. Correlation of the effect of HDAC6 inhibitors on proteasome activity with the effect on MHC-I antigen presentation. a.) Effect of tubastatin-A on proteasome activity correlated with the effect on pan HLA-ABC antigen presentation. b.) Effect of ACY-738 on proteasome activity correlation with the effect on pan HLA-ABC antigen presentation c.) Effect of ACY-1215 on proteasome activity correlated with the effect on pan HLA-ABC antigen presentation.

Figure S19 Fig. S19. Graphical representation to depict the effect of HDAC6 inhibitors on proteasome activity. HDAC6 inhibitors release HR23B which is bound to the HDAC6 BUZ domain. Free HR23B binds and shuttles ubiquitinated cargo proteins to the proteasome. Rad23 binds the proteasome through a UbL (ubiquitin-like) domain and contains UBA (ubiquitin-associated) motifs that bind multi-ubiquitin chains. These domains allow Rad23 to function as a substrate shuttle-factor. Shown is the association of HR23B with the 26S proteasome through interaction with the non-ATPase regulatory subunit 14, also known as Rpn11. The ability of HDAC6 to downregulate HR23B occurs independently of its deacetylase activity.

Figure S20 Fig. S20. SDS-PAGE of HDAC6 KO cells. MM cells were either untreated or treated with sgRNA scrambled control or sgRNA specific to HDAC6. Following selection, cell lysates were prepared and electrophoresed on SDS gels, transferred to PVDF. Membranes were probed for HDAC6 using a knockout verified antibody (catalog number ab133493, Abcam, Waltham, MA) a dilution of 1:10,000.
