# Supplementary material for: HDAC6 Inhibition Releases HR23B to Activate Proteasomes, Expand the Tumor Immunopeptidome and Amplify T-cell Antimyeloma Activity
Source: Cancer Res Commun. 2024 Jun 18;4(6):1517–32. doi: 10.1158/2767-9764.CRC-23-0528 (PMC11188874; doi:10.1158/2767-9764.CRC-23-0528)
Supplement: Table S1 — Top activators of proteasomal ChT-Like activity. Shown are the top pharmacologics that increased proteasome ChT-like in the HTS. [file crc-23-0528-s01.docx]

**Table S1. Activators of proteasomal activity detected in the cell-based screen**

**Compound** **Fold-Increase Class Description**

ACY-1215 (Ricolinostat) 2.2 HDAC6 inhibitor Induces α-tubulin hyperacetylation

Dipyridamole 2.0 PDE3 inhibitor Vasodilator

Salinomycin 2.0 Monovalent ionophore Polyether antibacterial, coccidiostat

Rapamycin 1.8 Macrolide Immunosuppressant, mTOR inhibitor

Abiraterone acetate 1.7 Cytochrome P450 inhibitor Steroidal cytochrome CYP17 inhibitor

Tubastatin A 1.7 HDAC6 inhibitor Induces α-tubulin hyperacetylation

DC-661 1.6 Dimeric CQ Palmitoyl-protein thioesterase 1 inhibitor

ACY-738 1.6 HDAC6 inhibitor Induces α-tubulin hyperacetylation

Sclareol 1.4 Bicyclic labdane diterpene Cytotoxic to tumor cells

1,10-phenanthroline monohydrate 1.3 Fe^++^/Zn^++^ chelator Metalloproteinase inhibitor

Riluzole 1.3 Stabilizes voltage-dependent Benzothiazole, glutamatergic antagonist

sodium channels First approved treatment for ALS

Betulinic acid 1.3 Pentacyclic lupane-type Natural product

Triterpene

Rolipram 1.3 PDE-4 inhibitor Antidepressant

Hydroxychloroquine 1.2 Aminoquinoline Anti-malarial, treat SLE, RA

2,3-Dimethoxy-1,4-naphthoquinone 1.2 Cell stress DNMQ, redox cycling agent, ROS

**Table S1.** Shown are the top pharmacologics that increased proteasome ChT-like in the HTS.
