# Supplementary material for: HDAC6 Inhibition Releases HR23B to Activate Proteasomes, Expand the Tumor Immunopeptidome and Amplify T-cell Antimyeloma Activity
Source: Cancer Res Commun. 2024 Jun 18;4(6):1517–32. doi: 10.1158/2767-9764.CRC-23-0528 (PMC11188874; doi:10.1158/2767-9764.CRC-23-0528)
Supplement: Table S2 — Top inhibitors of proteasomal ChT-like activity. Shown are the top pharmacologics that decreased proteasome ChT-like in the HTS. [file crc-23-0528-s02.docx]

**Table S2. Inhibitors of proteasomal activity detected in the cell-based screen**

**Compound** **Fold-Decrease Class Description**

Carfilzomib 54 Proteasome inhibitor PR-171, irreversible tetrapeptide epoxyketone PI.

Potency against the ChT-L activity in the β5 subunit.

BGT226 25 PI3K/mTOR inhibitor Novel class I PI3K/mTOR inhibitor for

PI3Kα/β/γ.

MG-132 16 Proteasome inhibitor Potent, reversible, cell-permeable PI.

YM155 11 Survivin suppressor Sepantronium bromide, potent antitumor activity.

Pacritinib 5 JAK2/Flt3 inhibitor Potent and selective inhibitor for MPD patients.

Calcimycin 5 Ca^++^ ionophore A23187, mobile ion-carrier that forms stable

complexes with divalent cations.

Bortezomib 4 Proteasome inhibitor Velcade, reversible, potent inhibitor of the activity

in the proteasome β5 subunit.

ONX-0914 4 Proteasome inhibitor PR-957, potent and selective inhibitor of

immunoproteasome with minimal cross-reactivity for the constitutive proteasome.

ONX-0912 4 Proteasome inhibitor Orally bioavailable inhibitor for ChT-L activity of

20S proteasome β5/LMP7.

Zolmitriptan 4 Selective serotonin receptor Zomig, 5HT_1_-receptor agonist.

Antagonist

Epirubicin 4 L-arabino doxorubicin Anthracycline drug used for chemotherapy.

Derivative

CEP-18770 (Delanzomib) 4 Proteasome inhibitor Reversible, P2 threonine boronic acid inhibitor of

the ChT-L activity of the proteasome.

**Table S2.** Shown are the top pharmacologics that decreased proteasome ChT-like in the HTS.
