# Supplementary material for: HDAC6 Inhibition Releases HR23B to Activate Proteasomes, Expand the Tumor Immunopeptidome and Amplify T-cell Antimyeloma Activity
Source: Cancer Res Commun. 2024 Jun 18;4(6):1517–32. doi: 10.1158/2767-9764.CRC-23-0528 (PMC11188874; doi:10.1158/2767-9764.CRC-23-0528)
Supplement: Table S3 — Target specificity of HDA6 inhibitors. Compounds were tested in 10-dose IC50 mode, in triplicate, with 3-fold serial dilution starting at 1 uM. The HDAC reference compound Trichostatin A (TSA) was tested in a 10-dose IC50 with 3-fold serial dilution starting at 10 uM. HDAC6 reference compound TMP269 was tested in a 10-dose IC50 with 3-fold serial dilution starting at 10 uM. HDAC10 reference compound Quinostat was tested in a 10-dose IC50 with 3-fold serial dilution starting at 10 uM. SIRT reference compound Nicotinamide was tested in a 10-dose IC50 with 3-fold serial dilution starting at 10 uM. SIRT reference compound Suramin was tested in a 10-dose IC50 with 3-fold serial dilution starting at 100 uM. Substrates used for HDAC 1, 2, 3, and 6 were fluorogenic peptide p53 (residues 379-382) RHKK(Ac)-AMC; HDAC 4, 5, 7, 9, 11 were fluorogenic HDAC classic 2a substrate trifluoroacetyl lysine; HDAC8 was fluorogenic peptide p53 residues 379-382 and NAD; HDAC10 was Ac- spermidine-AMC; SIRTs 1, 2, and 3- fluorogenic peptide p53 residues 379-382 and NAD; SIRT5- fluorogenic peptide Ac-Lys-succ and NAD. Curve fits were performed where enzyme activities at the highest compound concentration were <65%. No inhibition indicates that compound activity was negligible and could not be fit to an IC50 curve. IC50 value higher than 1.00E-06 M was estimated based on the best curve fitting available. Assays were determined in collaboration with Reaction Biology (Malvern, PA). [file crc-23-0528-s03.docx]

| **Table S3** |  |  |  |  |  |  |  |  |  |  |  |  |  |  |  |  |
| --- | --- | --- | --- | --- | --- | --- | --- | --- | --- | --- | --- | --- | --- | --- | --- | --- |
|  |  |  |  |  |  |  |  |  |  |  |  |  |  |  |  |  |
|  |  |  |  |  |  |  |  |  |  |  |  |  |  |  |  |  |
|  |  |  |  |  |  |  |  |  |  |  |  |  |  |  |  |  |
| **Compound ID** | **Target:** | **HDAC1** | **HDAC2** | **HDAC3** | **HDAC4** | **HDAC5** | **HDAC6** | **HDAC7** | **HDAC8** | **HDAC9** | **HDAC10** | **HDAC11** | **SIRT1** | **SIRT2** | **SIRT3** | **SIRT5** |
|  |  |  |  |  |  |  |  |  |  |  |  |  |  |  |  |  |
| **Tubastatin-A** | **Data 1** |  |  | > 1.00E-06 | > 1.00E-06 | > 1.00E-06 | 8.37E-10 | 6.53E-07 | > 1.00E-06 | 9.84E-07 | 6.51E-09 |  |  |  |  |  |
|  | **Data 2** |  |  | > 1.00E-06 | > 1.00E-06 | > 1.00E-06 | 1.08E-09 | 5.33E-07 | > 1.00E-06 | > 1.00E-06 | 7.40E-09 |  |  |  |  |  |
|  | **Data 3** |  |  | > 1.00E-06 | > 1.00E-06 | > 1.00E-06 | 9.88E-10 | 5.45E-07 | > 1.00E-06 | > 1.00E-06 | 7.42E-09 |  |  |  |  |  |
| **ACY-1215** | **Data 1** | 3.29E-07 | 6.86E-07 | 2.30E-07 |  |  | 2.64E-09 |  | 7.32E-07 |  | 3.33E-07 |  |  |  |  |  |
|  | **Data 2** | 2.89E-07 | 5.34E-07 | 2.64E-07 |  |  | 3.16E-09 |  | 6.19E-07 |  | 3.56E-07 |  |  |  |  |  |
|  | **Data 3** | 3.29E-07 | 5.53E-07 | 2.80E-07 |  |  | 2.05E-09 |  | 4.74E-07 |  | 3.68E-07 |  |  |  |  |  |
| **Trichostatin A** |  | 1.51E-09 | 3.45E-09 | 1.65E-09 | ND | ND | 6.52E-10 | ND | 2.38E-07 | ND | ND | 5.13E-06 | ND | ND | ND | ND |
| **TMP 269** |  | ND | ND | ND | 1.05E-07 | 1.10E-07 | ND | 3.81E-08 | ND | 1.20E-08 | ND | ND | ND | ND | ND | ND |
| **Suramin** |  | ND | ND | ND | ND | ND | ND | ND | ND | ND | ND | ND | 2.68E-06 | 2.43E-05 | ND | ND |
| **Nicotinamide** |  | ND | ND | ND | ND | ND | ND | ND | ND | ND | ND | ND | ND | ND | 1.93E-05 | 3.12E-05 |
| **Quisinostat** |  | ND | ND | ND | ND | ND | ND | ND | ND | ND | 4.89E-09 | ND | ND | ND | ND | ND |
|  |  |  |  |  |  |  |  |  |  |  |  |  |  |  |  |  |
|  |  |  |  |  |  |  |  |  |  |  |  |  |  |  |  |  |
|  |  |  |  |  | **Values represent the IC50 (M)**  *** Empty cells indicate that no inhibition was observed, or that compound activity could not be fit to an IC50 curve** | | | | | | | | |  |  |  |
|  |  |  |  |  | **** IC50 value higher than 1.00E-06 M was estimated based on the best curve fitting available.** | | | | | | |  |  |  |  |  |
|  |  |  |  |  | **ND** | **Indicates compound not tested against enzyme** | | | |  |  |  |  |  |  |  |
|  |  |  |  |  | **Results were obtained in collaboration with Reaction Biology, Malvern, PA.** | | | | | |  |  |  |  |  |  |

**Table S3. Target specificity of HDA6 inhibitors.** Compounds were tested in 10-dose IC50 mode, in triplicate, with 3-fold serial dilution starting at 1 uM. The HDAC reference compound Trichostatin A (TSA) was tested in a 10-dose IC50 with 3-fold serial dilution starting at 10 uM. HDAC6 reference compound TMP269 was tested in a 10-dose IC50 with 3-fold serial dilution starting at 10 uM. HDAC10 reference compound Quinostat was tested in a 10-dose IC50 with 3-fold serial dilution starting at 10 uM. SIRT reference compound Nicotinamide was tested in a 10-dose IC50 with 3-fold serial dilution starting at 10 uM. SIRT reference compound Suramin was tested in a 10-dose IC50 with 3-fold serial dilution starting at 100 uM. Substrates used for HDAC 1, 2, 3, and 6 were fluorogenic peptide p53 (residues 379-382) RHKK(Ac)-AMC; HDAC 4, 5, 7, 9, 11 were fluorogenic HDAC classic 2a substrate trifluoroacetyl lysine; HDAC8 was fluorogenic peptide p53 residues 379-382 and NAD; HDAC10 was Ac- spermidine-AMC; SIRTs 1, 2, and 3- fluorogenic peptide p53 residues 379-382 and NAD; SIRT5- fluorogenic peptide Ac-Lys-succ and NAD. Curve fits were performed where enzyme activities at the highest compound concentration were <65%. No inhibition indicates that compound activity was negligible and could not be fit to an IC50 curve. IC50 value higher than 1.00E-06 M was estimated based on the best curve fitting available. Assays were determined in collaboration with Reaction Biology (Malvern, PA).
