# Supplementary material for: HDAC6 Inhibition Releases HR23B to Activate Proteasomes, Expand the Tumor Immunopeptidome and Amplify T-cell Antimyeloma Activity
Source: Cancer Res Commun. 2024 Jun 18;4(6):1517–32. doi: 10.1158/2767-9764.CRC-23-0528 (PMC11188874; doi:10.1158/2767-9764.CRC-23-0528)
Supplement: Table S4 — Proteasomal Fluorogenic Peptide Substrates. Shown are the fluorogenic substrates, catalytic site preferences and excitation and emission values used for detection of substrate hydrolysis. [file crc-23-0528-s04.docx]

**Table S4. Proteasomal Fluorogenic Peptide Substrates**

**Substrate Preferentially cleaved by**

LLVY-R110 β5c, β5i

Suc-LLVY-MCA β5c, β5i

Ac-WLA-MCA β5c

ANC-ANW-MCA β5i

Boc-LRR-MCA β2c, β2i

Z-ARR-MCA β2c, β2i

Z-LLE-MCA β1c

ANC-PAL-MCA β1i

**Table S4.** Shown are the fluorogenic substrates, catalytic site preferences and excitation and emission values used for detection of substrate hydrolysis.
