# Supplementary material for: HDAC6 Inhibition Releases HR23B to Activate Proteasomes, Expand the Tumor Immunopeptidome and Amplify T-cell Antimyeloma Activity
Source: Cancer Res Commun. 2024 Jun 18;4(6):1517–32. doi: 10.1158/2767-9764.CRC-23-0528 (PMC11188874; doi:10.1158/2767-9764.CRC-23-0528)
Supplement: Table S5 — NeoAgs regulated by proteasome activation or inhibition. Shown is the peptide sequence, gene accession number, mutation or PTM fold change in that peptide antigen following treatment with either tubastatin-A, ACY-738, or bortezomib. Green indicates upregulation and red indicates downregulation. Numbers indicate the fold-change relative to untreated RPMI-8226 cells. Arrows indicate the direction of change for peptide sequences that were not detected in untreated cells. ND (blue) indicates that the peptide was not detected under the indicated condition. Blue asterisks indicate that the antigen was not detected in untreated cells and unmasked after treatment with the HDAC6 inhibitor. [file crc-23-0528-s05.docx]

**Table S5. Neoantigens regulated by proteasome activation or inhibition**

| **No.** | **Peptide sequence** | **Accession** | **Mutation/ PTM** | **Tuba-A** | **ACY-738** | **BTZ** |
| --- | --- | --- | --- | --- | --- | --- |
| 1 | KKFWPNPCSTY(F)C | *S41A3* | Mutation | ↑ | - | - |
| 2 | GQKPWSQHYHQGYY | *HNRPU* | G1: Acetylation (N-term) | ↑ | - | - |
| 3 | Q(G)LIWVVGP | *PTPRD* | Mutation | ↑ | - | - |
| 4 | VW(A)HILQTLSAPTKNLEQQVN | *PAXI1* | Mutation | ↑ | - | - |
| 5 | PSH(A)LDSADLPPPSAL | *NF2L1* | Mutation | ↑ | - | - |
| 6 | MHGQPSPSL | *T126B* | M1: Oxidation | ↑ | - | - |
| 7 | MHGQPSPSL | *T126B* | M1: Oxidation | ↑ | - | - |
| 8 | S(Y)HNPKIALL | *TCPH* | Mutation | ↑ | - | - |
| 9 | E(Q)IFHPEQLITGKEDAAN | *TBA4B* | Mutation | ↑ | - | - |
| 10 | E(Q)LFHPEQLITGKEDAAN | *TBA1B, TBA1C, TBA1A, TBA3D, TBA3C, TBA4A, TBA8* | Mutation | ↑ | - | - |
| 11 | VH(T)HVNALTV | *B3A3* | Mutation | ↑ | - | - |
| 12 | VHVD(N)TSAVL | *POMT1* | Mutation | ↑ | - | - |
| 13 | VALPTPALSPSL | *AP3B1* | V1: Acetylation (N-term) | ↑ | - | - |
| 14 | YKLD(N)KTHSAY | *CR2* | Mutation | ↑ | - | - |
| 15 | A(L)KNFNPTVNY | *SSRG* | Mutation | ↑ | - | - |
| 16 | E(Q)IPIISAEHLT | *MBNL1* | Mutation | ↑ | - | - |
| 17 | C(I)MHDCVVKLL | *IF4G3* | M2: Oxidation | ↑ | - | - |
| 18 | MHN(S)GMNATTI | *GNPTA* | Mutation | ↑ | - | - |
| 19 | YVY(R)PGLPVTF | *TPRN* | Mutation | ↑ | - | - |
| 20 | N(F)VNDIFERI | *H2B1, H2B2, H2B3* | Mutation | - | ↑ | - |
| 21 | FVD(N)TSAPRQL | *ICAM3* | Mutation | - | ↑ | - |
| 22 | SWE(L)PRPIPK | *CBL* | Mutation | - | ↑ | - |
| 23 | SQLDE(S)CVHP | *KANL1* | Mutation | - | ↑ | - |
| 24 | HKSDPYR(S)TG | *BRD4* | Mutation | - | ↑ | - |
| 25 | YAAMFGPKGFGRGGAET(S)HTF | *CRIP1* | Mutation | - | ↑ | - |
| 26 | GA(C)RGSFIPARY | *SKOR2* | Mutation | - | ↑ | - |
| 27 | ETFSGVYKL(K) | *\|RS7* | Mutation | ↑ | ↑ | - |
| 28 | GRSKGFGFM(V)CFS | *PABP1, PABP3, PABP4* | Mutation | ↑ | ↑ | - |
| 29 | E(Q)AAPAFGFGSSQAAT | *NUP42* | Mutation | ↑ | ↑ | ↑ |
| 30 | E(Q)VANPNSAIFGGARPREEVVQKEQE | *IF4H* | Mutation | ↑ | - | ↑ |
| 31 | NASSLLDIYSFWLR(K)SAKVPERKLQAN | *NOLC1* | Mutation | - | ↓ | ↓ |
| 32 | GKFL(T)EGNITY | *ZCCHV* | Mutation | ↑ | ↑ | ↓ |

**Table S5. NeoAgs regulated by proteasome activation or inhibition.** Shown is the peptide sequence, gene accession number, mutation or PTM fold change in that peptide antigen following treatment with either tubastatin-A, ACY-738, or bortezomib. Green indicates upregulation and red indicates downregulation. Numbers indicate the fold-change relative to untreated RPMI-8226 cells. Arrows indicate the direction of change for peptide sequences that were not detected in untreated cells. ND (blue) indicates that the peptide was not detected under the indicated condition. Blue asterisks indicate that the antigen was not detected in untreated cells and unmasked after treatment with the HDAC6 inhibitor.
