# Supplementary material for: HDAC6 Inhibition Releases HR23B to Activate Proteasomes, Expand the Tumor Immunopeptidome and Amplify T-cell Antimyeloma Activity
Source: Cancer Res Commun. 2024 Jun 18;4(6):1517–32. doi: 10.1158/2767-9764.CRC-23-0528 (PMC11188874; doi:10.1158/2767-9764.CRC-23-0528)
Supplement: Figure S1 — Fig. S1. Conversion of the constitutive proteasome to immunoproteasome by IFN-γ-induced subunit substitution. [file crc-23-0528-s07.pptx]

## Slide 1
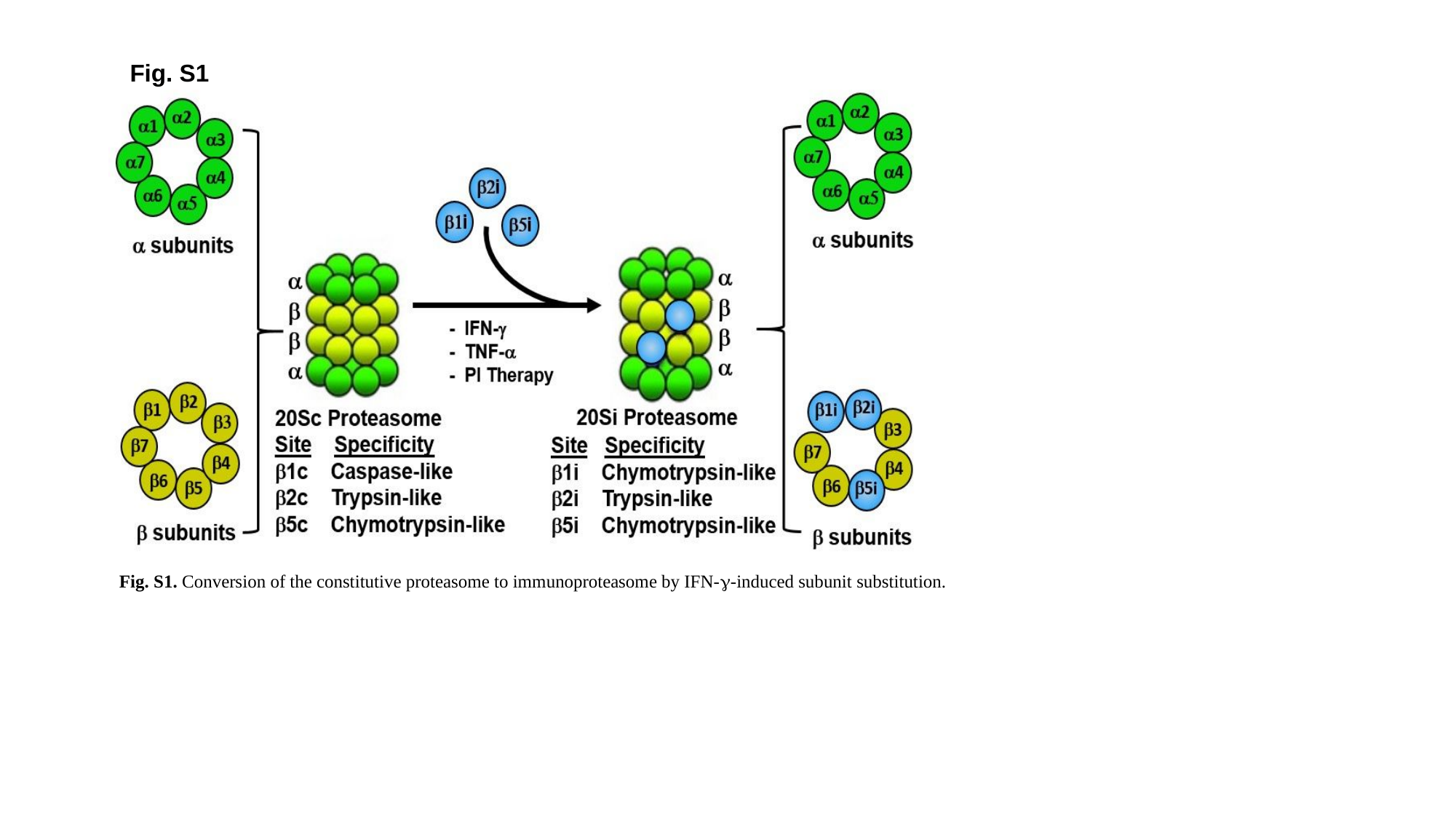

Fig. S1
Fig. S1. Conversion of the constitutive proteasome to immunoproteasome by IFN-g-induced subunit substitution.
