# Supplementary material for: HDAC6 Inhibition Releases HR23B to Activate Proteasomes, Expand the Tumor Immunopeptidome and Amplify T-cell Antimyeloma Activity
Source: Cancer Res Commun. 2024 Jun 18;4(6):1517–32. doi: 10.1158/2767-9764.CRC-23-0528 (PMC11188874; doi:10.1158/2767-9764.CRC-23-0528)
Supplement: Figure S2 — Fig. S2. a. Correlation of proteasome ChT-like activity with cell number. Proteasome ChT-like activity was determined with indicated number of RPMI-8226 cells at 24 hrs. b. Correlation of proteasome ChT-like activity with incubation time. Proteasome ChT-like activity was determined with 50,000 RPMI-8226 cells/well at indicated time points. c. Correlation of proteasome ChT-like activity with LLVY-R110 concentration. Proteasome ChT-like activity was determined with 50,000 RPMI-8226 cells/well after 24 h of incubation. d. Effect of DMSO (%) on proteasome activity in three MMCLs. [file crc-23-0528-s08.pptx]

## Slide 1
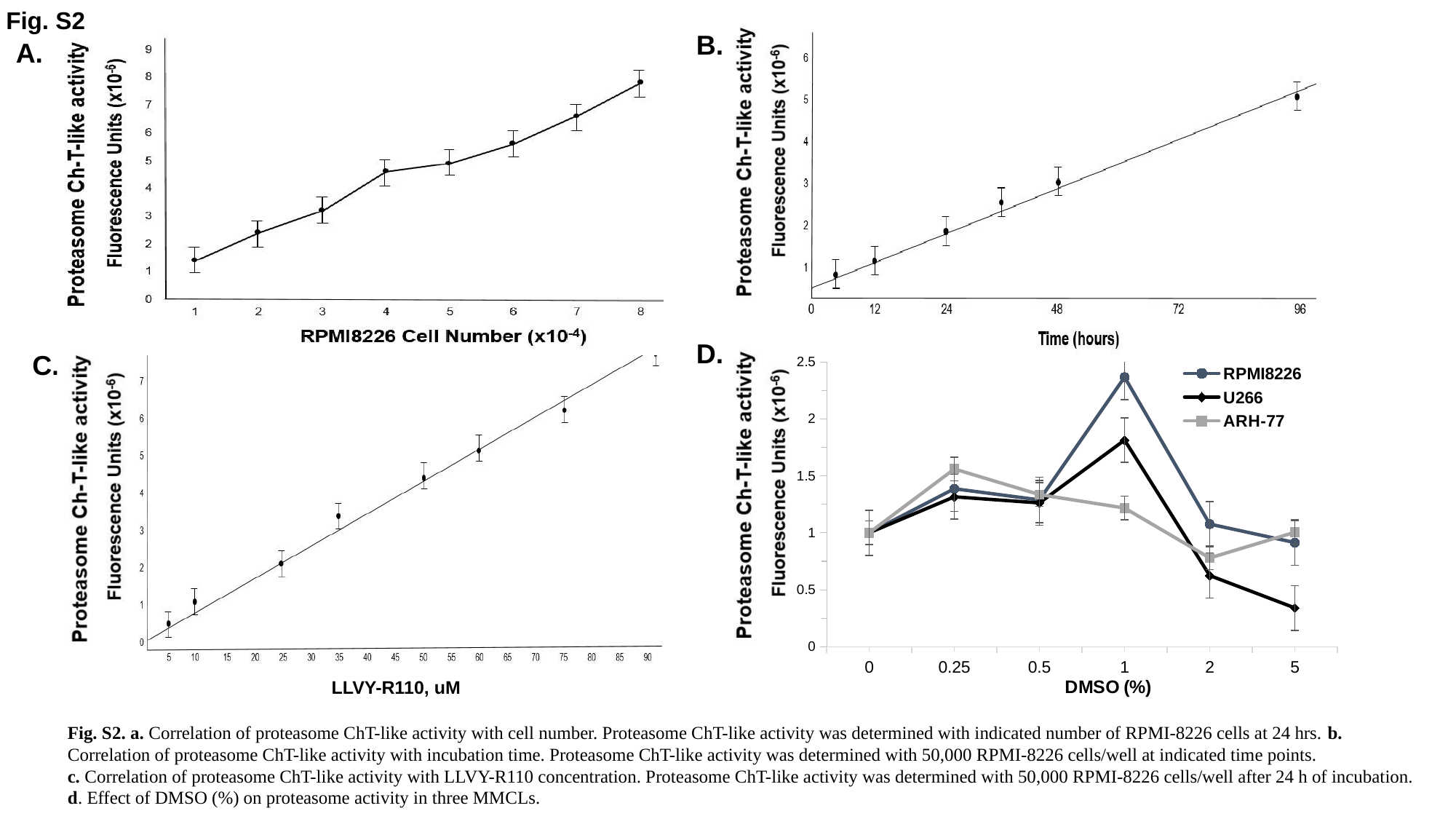

Fig. S2
B.
A.
D.
### Chart
| Category | RPMI8226 | U266 | ARH-77 |
|---|---|---|---|
| 0 | 1.0 | 1.0 | 1.0 |
| 0.25 | 1.3872846929697633 | 1.3162925695010175 | 1.5603785710112754 |
| 0.5 | 1.28892676077473 | 1.2627099563200794 | 1.3357383497887088 |
| 1 | 2.366474806263867 | 1.8138056386806785 | 1.217736752935678 |
| 2 | 1.0768245495567919 | 0.6250895499780988 | 0.77948822807735 |
| 5 | 0.9142523339442853 | 0.34093262976047695 | 1.0045568732838699 |C.
LLVY-R110, uM
Fig. S2. a. Correlation of proteasome ChT-like activity with cell number. Proteasome ChT-like activity was determined with indicated number of RPMI-8226 cells at 24 hrs. b. Correlation of proteasome ChT-like activity with incubation time. Proteasome ChT-like activity was determined with 50,000 RPMI-8226 cells/well at indicated time points.
c. Correlation of proteasome ChT-like activity with LLVY-R110 concentration. Proteasome ChT-like activity was determined with 50,000 RPMI-8226 cells/well after 24 h of incubation. d. Effect of DMSO (%) on proteasome activity in three MMCLs.
