# Supplementary material for: HDAC6 Inhibition Releases HR23B to Activate Proteasomes, Expand the Tumor Immunopeptidome and Amplify T-cell Antimyeloma Activity
Source: Cancer Res Commun. 2024 Jun 18;4(6):1517–32. doi: 10.1158/2767-9764.CRC-23-0528 (PMC11188874; doi:10.1158/2767-9764.CRC-23-0528)
Supplement: Figure S3 — Fig. S3. Chemical structure of top pharmacologics identified in the HTS that increased proteasome ChT-like activity. [file crc-23-0528-s09.pptx]

## Slide 1
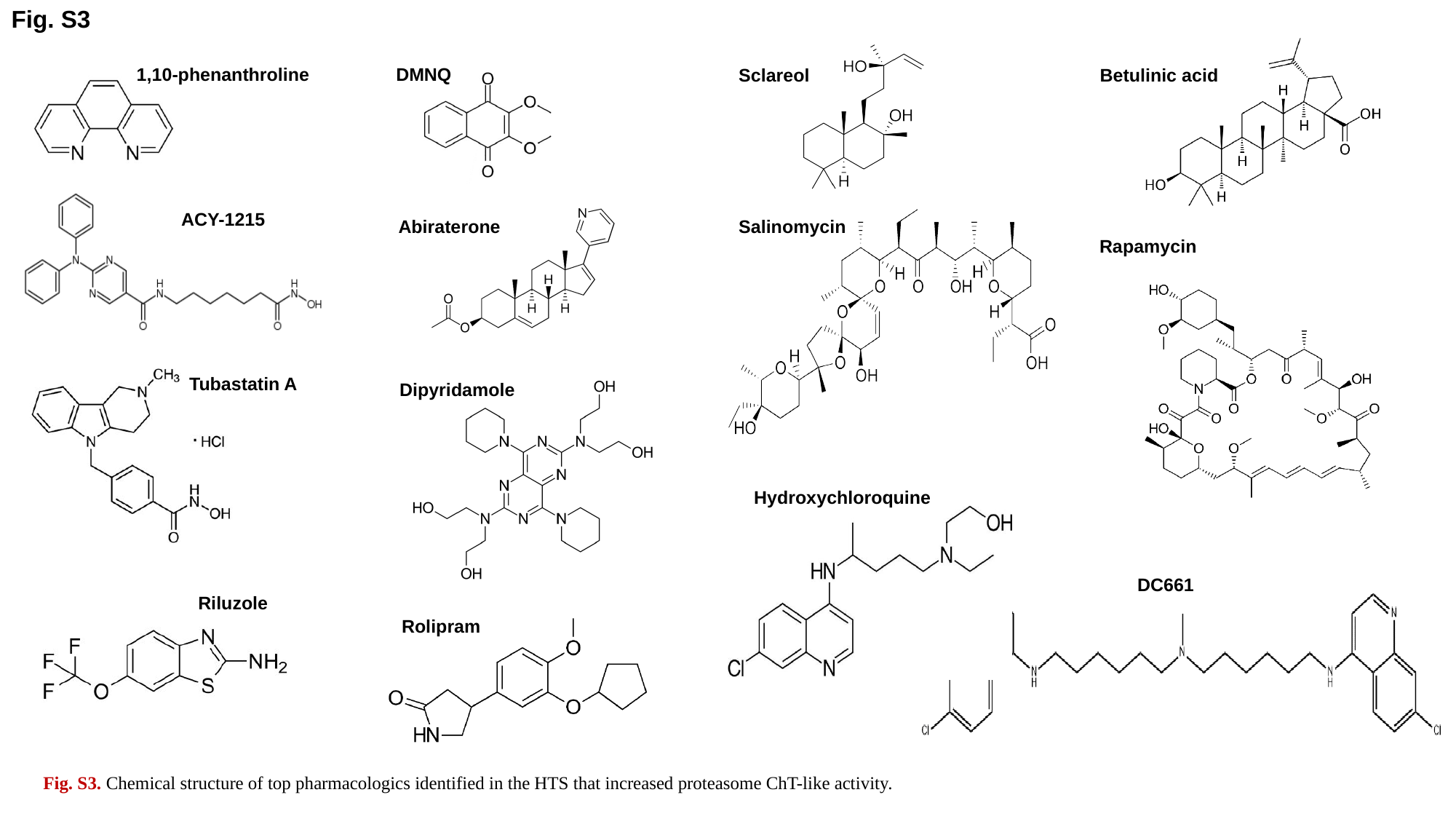

Fig. S3
Sclareol
Betulinic acid
1,10-phenanthroline
DMNQ
Salinomycin
ACY-1215
Abiraterone
Rapamycin
Tubastatin A
Dipyridamole
Hydroxychloroquine
DC661
Riluzole
Rolipram
Fig. S3. Chemical structure of top pharmacologics identified in the HTS that increased proteasome ChT-like activity.
