# Supplementary material for: HDAC6 Inhibition Releases HR23B to Activate Proteasomes, Expand the Tumor Immunopeptidome and Amplify T-cell Antimyeloma Activity
Source: Cancer Res Commun. 2024 Jun 18;4(6):1517–32. doi: 10.1158/2767-9764.CRC-23-0528 (PMC11188874; doi:10.1158/2767-9764.CRC-23-0528)
Supplement: Figure S4 — Fig. S4. a. Effect of hits from the HTS on proteasome ChT-like activity on ARH-77 and U266 MMCLs. Proteasome ChT-like activity was determined with 50,000 ARHG-77 or U266 cells/well after 24 h of incubation with 50 uM LLVY-R110. b. Effect of proteasome inhibitors on proteasome ChT-like activity in three different MMCLs. Bortezomib, carfilzomib and ixazomib were added to MM cells at indicated concentrations. Proteasome ChT-like activity was then determined after 24 h incubation. [file crc-23-0528-s10.pptx]

## Slide 1
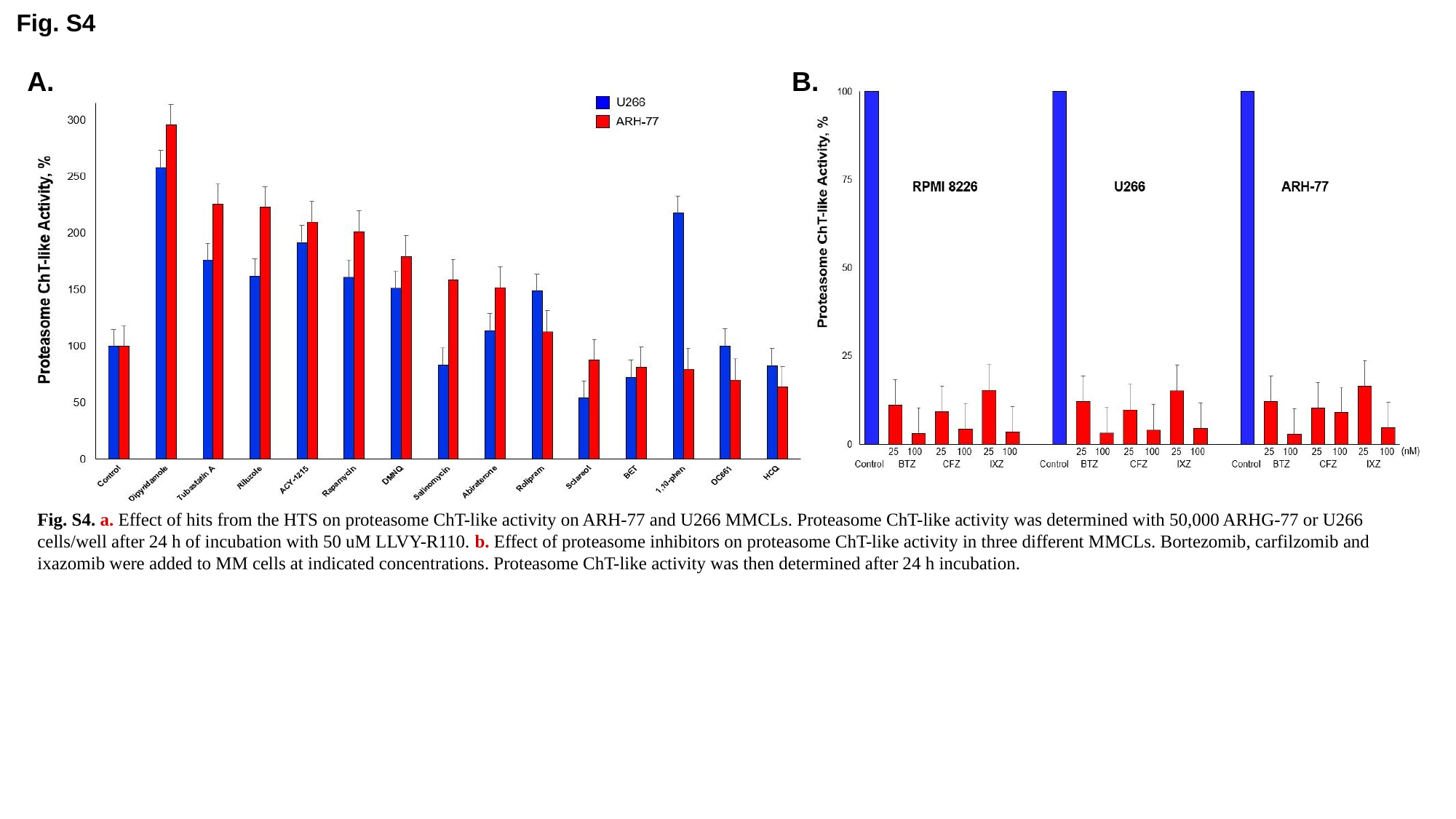

Fig. S4
A.
B.
Fig. S4. a. Effect of hits from the HTS on proteasome ChT-like activity on ARH-77 and U266 MMCLs. Proteasome ChT-like activity was determined with 50,000 ARHG-77 or U266 cells/well after 24 h of incubation with 50 uM LLVY-R110. b. Effect of proteasome inhibitors on proteasome ChT-like activity in three different MMCLs. Bortezomib, carfilzomib and ixazomib were added to MM cells at indicated concentrations. Proteasome ChT-like activity was then determined after 24 h incubation.
