# Supplementary material for: HDAC6 Inhibition Releases HR23B to Activate Proteasomes, Expand the Tumor Immunopeptidome and Amplify T-cell Antimyeloma Activity
Source: Cancer Res Commun. 2024 Jun 18;4(6):1517–32. doi: 10.1158/2767-9764.CRC-23-0528 (PMC11188874; doi:10.1158/2767-9764.CRC-23-0528)
Supplement: Figure S5 — Fig. S5. Effect of top pharmacologics on a. inhibition of proteasome ChT-like activity in MM cells and b. MM cell viability. [file crc-23-0528-s11.pptx]

## Slide 1
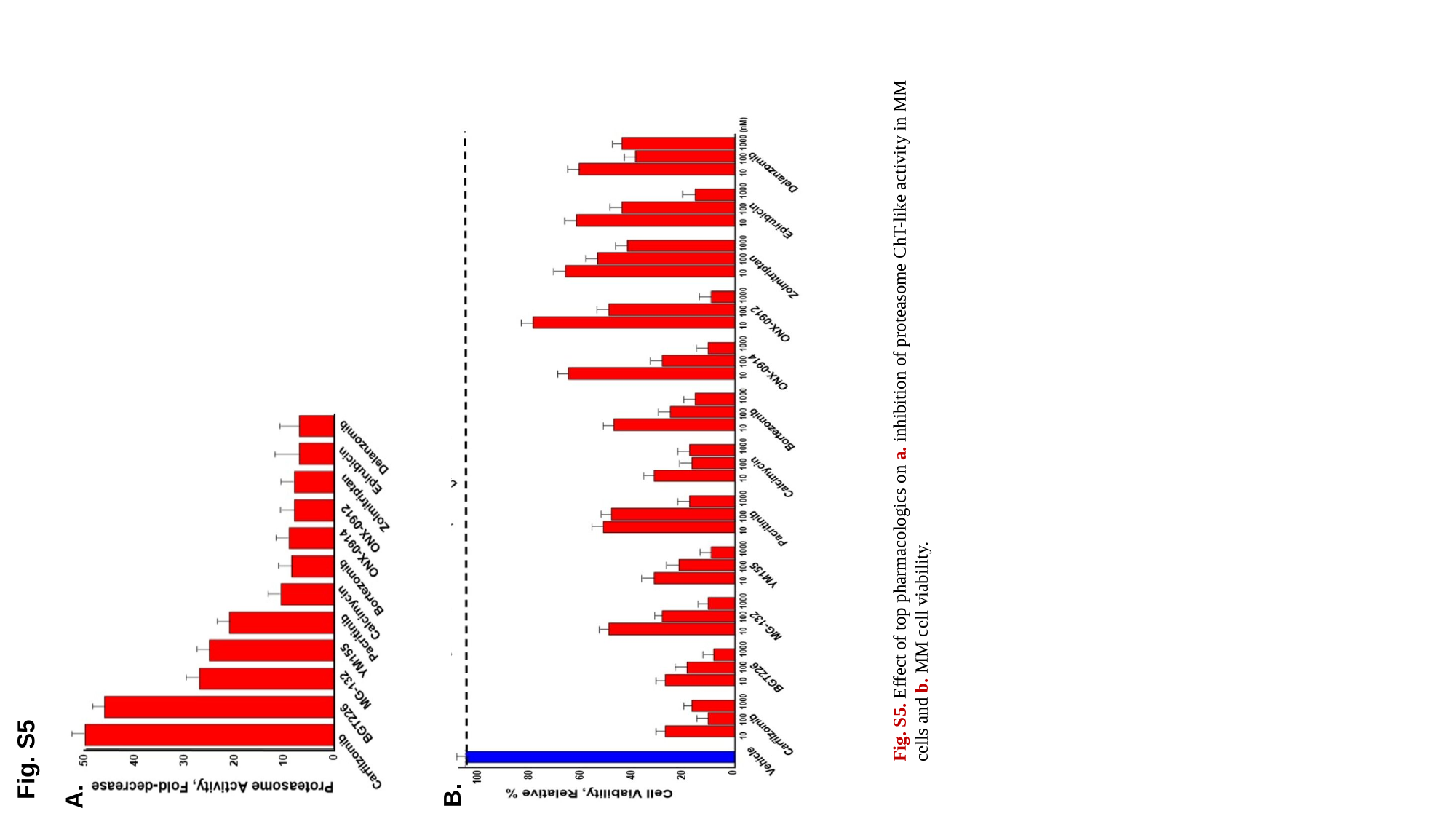

Fig. S5. Effect of top pharmacologics on a. inhibition of proteasome ChT-like activity in MM cells and b. MM cell viability.
Fig. S5
B.
A.
