# Supplementary material for: HDAC6 Inhibition Releases HR23B to Activate Proteasomes, Expand the Tumor Immunopeptidome and Amplify T-cell Antimyeloma Activity
Source: Cancer Res Commun. 2024 Jun 18;4(6):1517–32. doi: 10.1158/2767-9764.CRC-23-0528 (PMC11188874; doi:10.1158/2767-9764.CRC-23-0528)
Supplement: Figure S6 — Fig. S6. Dose-dependent effect of ACY-1215 and tubastatin-A on the biochemical activity of the HDACs and SIRTs. [file crc-23-0528-s12.pptx]

## Slide 1
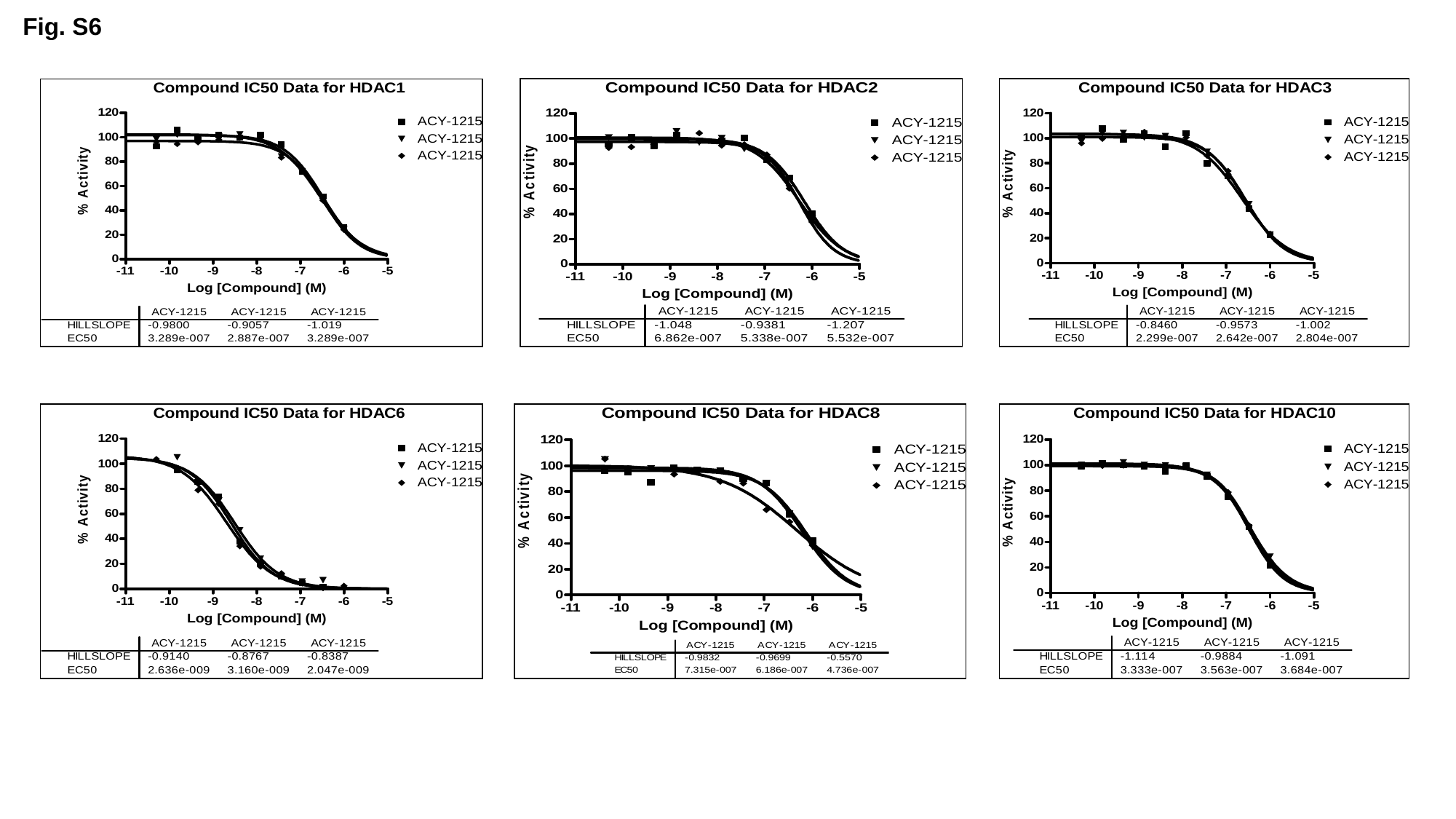

Fig. S6

## Slide 2
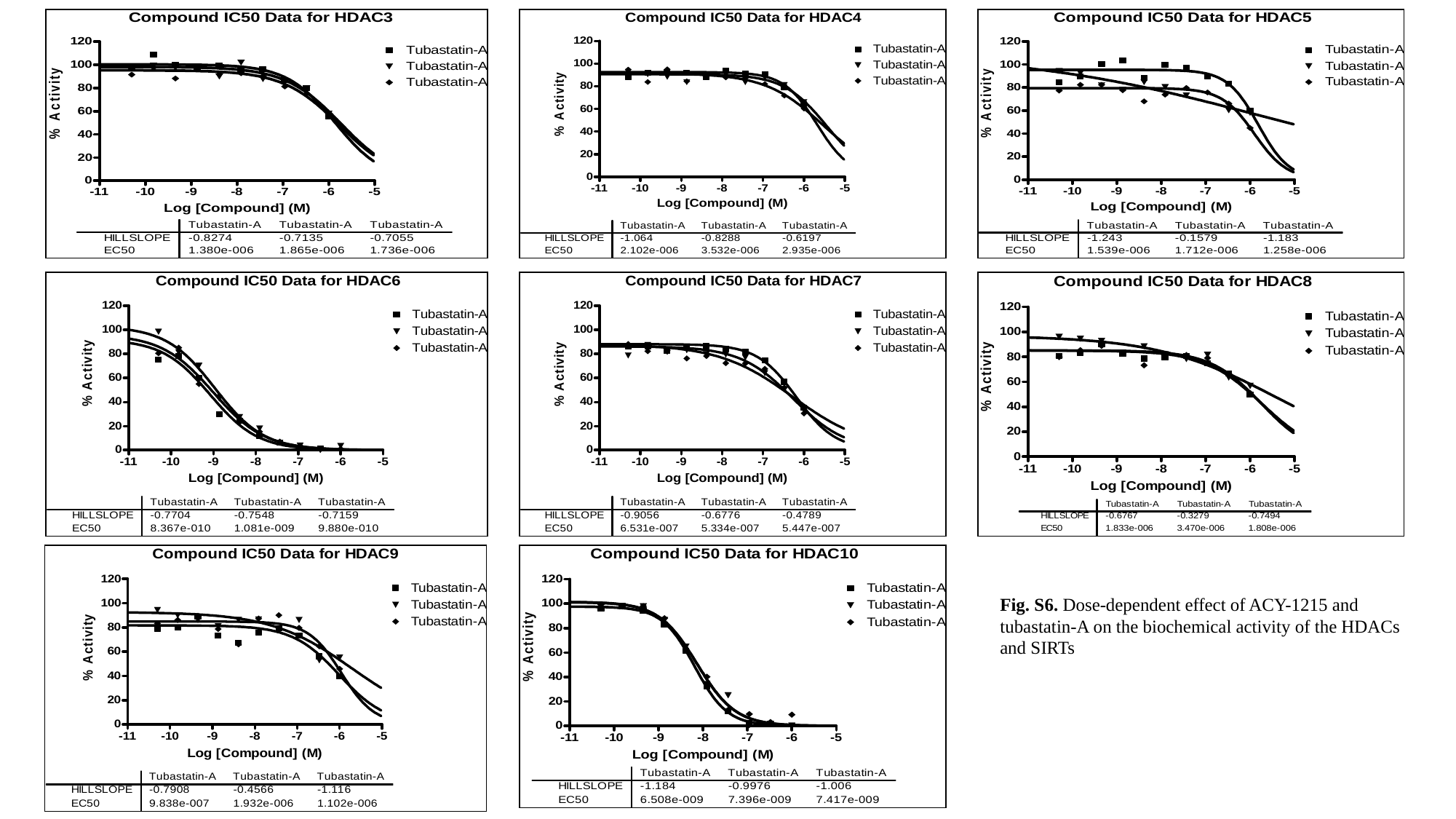

Fig. S6. Dose-dependent effect of ACY-1215 and tubastatin-A on the biochemical activity of the HDACs and SIRTs
