# Supplementary figures and images for: HDAC6 Inhibition Releases HR23B to Activate Proteasomes, Expand the Tumor Immunopeptidome and Amplify T-cell Antimyeloma Activity
Source: Cancer Res Commun. 2024 Jun 18;4(6):1517–32. doi: 10.1158/2767-9764.CRC-23-0528 (PMC11188874; doi:10.1158/2767-9764.CRC-23-0528)

## Slide 1
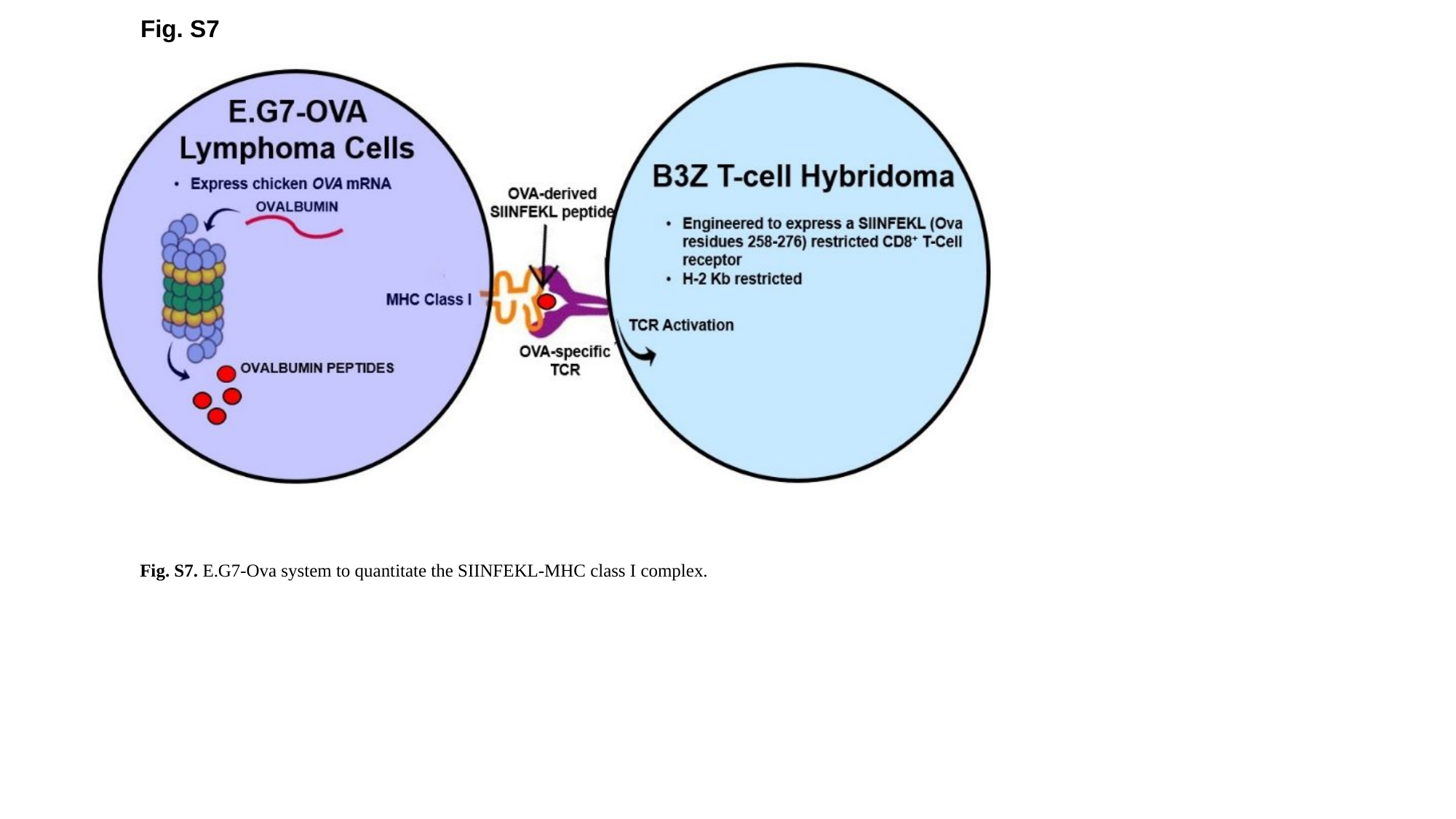

Fig. S7
Fig. S7. E.G7-Ova system to quantitate the SIINFEKL-MHC class I complex.

Supplement: Figure S7 — Fig. S7. E.G7-Ova system to quantitate the SIINFEKL-MHC class I complex. [file crc-23-0528-s13.pptx]
