# Supplementary material for: HDAC6 Inhibition Releases HR23B to Activate Proteasomes, Expand the Tumor Immunopeptidome and Amplify T-cell Antimyeloma Activity
Source: Cancer Res Commun. 2024 Jun 18;4(6):1517–32. doi: 10.1158/2767-9764.CRC-23-0528 (PMC11188874; doi:10.1158/2767-9764.CRC-23-0528)
Supplement: Figure S8 — Fig. S8. Effect of top pharmacologics from the HTS that inhibited proteasome ChT-like activity in E.G7-Ova and EL4 cells. Cells (50,000/well) were incubated with each pharmacologic for 72 hrs. Proteasome ChT-like activity was determined after 24 hrs incubation. E.G7-Ova and EL4 cells. Cells (50,000/well) were incubated with each pharmacologic for 72 h. Proteasome ChT-like activity was determined after 24 h incubation. [file crc-23-0528-s14.pptx]

## Slide 1
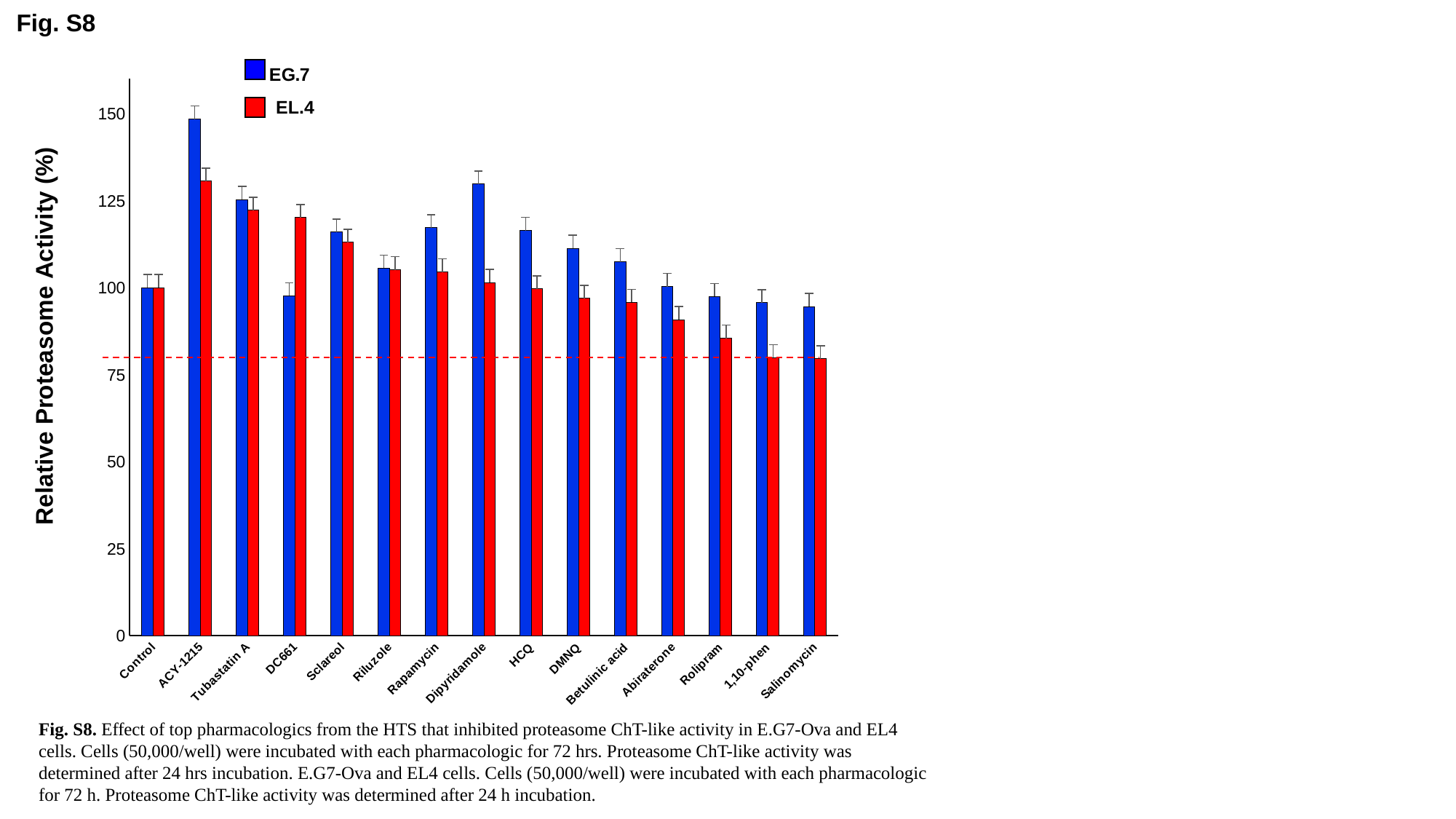

Fig. S8
### Chart
| Category | | |
|---|---|---|
| Control | 100.0 | 100.0 |
| ACY-1215 | 148.4765460098832 | 130.596149 |
| Tubastatin A | 125.34599498390048 | 122.232406 |
| DC661 | 97.66784481545555 | 120.159096 |
| Sclareol | 115.94908576205809 | 113.063699 |
| Riluzole | 105.60317355207722 | 105.135623 |
| Rapamycin | 117.25277499565436 | 104.501815 |
| Dipyridamole | 129.79095446606686 | 101.464299 |
| HCQ | 116.41510292936903 | 99.6192773 |
| DMNQ | 111.27422171822103 | 96.9004894 |
| Betulinic acid | 107.40867967320857 | 95.7522434 |
| Abiraterone | 100.33026793917772 | 90.8215685 |
| Rolipram | 97.44456216735232 | 85.47346 |
| 1,10-phen | 95.68292622360546 | 79.8968207 |
| Salinomycin | 94.56237428710962 | 79.5586437 |
EL.4
Fig. S8. Effect of top pharmacologics from the HTS that inhibited proteasome ChT-like activity in E.G7-Ova and EL4 cells. Cells (50,000/well) were incubated with each pharmacologic for 72 hrs. Proteasome ChT-like activity was determined after 24 hrs incubation. E.G7-Ova and EL4 cells. Cells (50,000/well) were incubated with each pharmacologic for 72 h. Proteasome ChT-like activity was determined after 24 h incubation.
