# Supplementary material for: HDAC6 Inhibition Releases HR23B to Activate Proteasomes, Expand the Tumor Immunopeptidome and Amplify T-cell Antimyeloma Activity
Source: Cancer Res Commun. 2024 Jun 18;4(6):1517–32. doi: 10.1158/2767-9764.CRC-23-0528 (PMC11188874; doi:10.1158/2767-9764.CRC-23-0528)
Supplement: Figure S9 — Fig. S9. Effect of the top pharmacologics that increased proteasome ChT-like activity on presentation of the SIINFEKL-MHC class I molecule complex. E.G7-Ova cells were treated with pharmacologics at 3 uM for 72 h. Cells were then stained with a monoclonal antibody to Ova 257-264 (SIINFEKL) peptide bound to H2Kb and quantitated using a BD-LSRII sorter interfaced with FlowJo software. [file crc-23-0528-s15.pptx]

## Slide 1
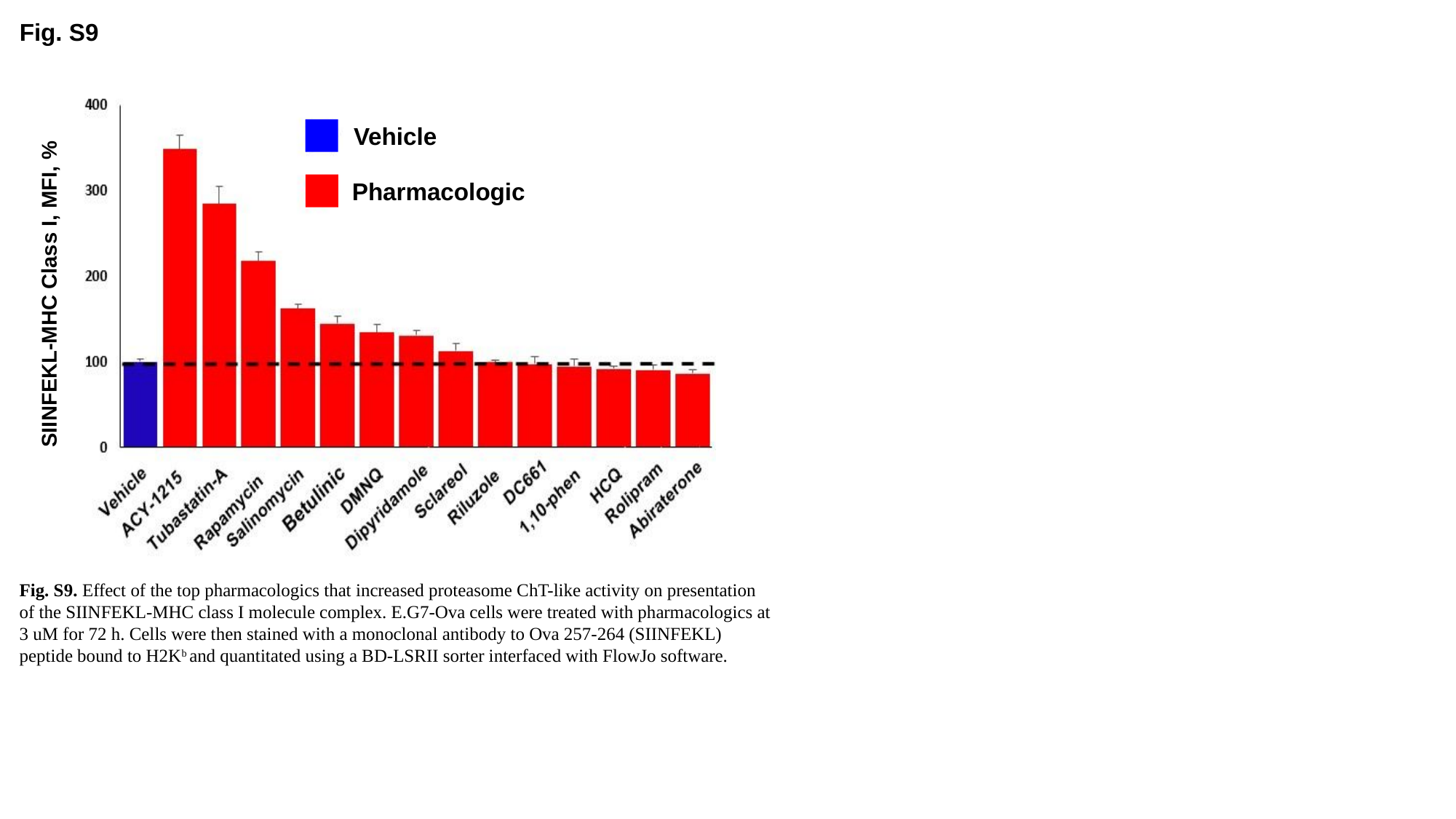

Fig. S9
Vehicle
Pharmacologic
SIINFEKL-MHC Class I, MFI, %
Fig. S9. Effect of the top pharmacologics that increased proteasome ChT-like activity on presentation of the SIINFEKL-MHC class I molecule complex. E.G7-Ova cells were treated with pharmacologics at 3 uM for 72 h. Cells were then stained with a monoclonal antibody to Ova 257-264 (SIINFEKL) peptide bound to H2Kb and quantitated using a BD-LSRII sorter interfaced with FlowJo software.
