# Supplementary material for: HDAC6 Inhibition Releases HR23B to Activate Proteasomes, Expand the Tumor Immunopeptidome and Amplify T-cell Antimyeloma Activity
Source: Cancer Res Commun. 2024 Jun 18;4(6):1517–32. doi: 10.1158/2767-9764.CRC-23-0528 (PMC11188874; doi:10.1158/2767-9764.CRC-23-0528)
Supplement: Figure S10 — Fig. S10. Effect of proteasome inhibitors on presentation of the SIINFEKL-MHC class I molecule complex. E.G7-Ova cells were treated with pharmacologics at the indicated concentration for 16 h. Cells were then stained with a monoclonal antibody to Ova 257-264 (SIINFEKL) peptide bound to H2Kb and quantitated using a BD-LSRII sorter interfaced with FlowJo software as above. [file crc-23-0528-s16.pptx]

## Slide 1
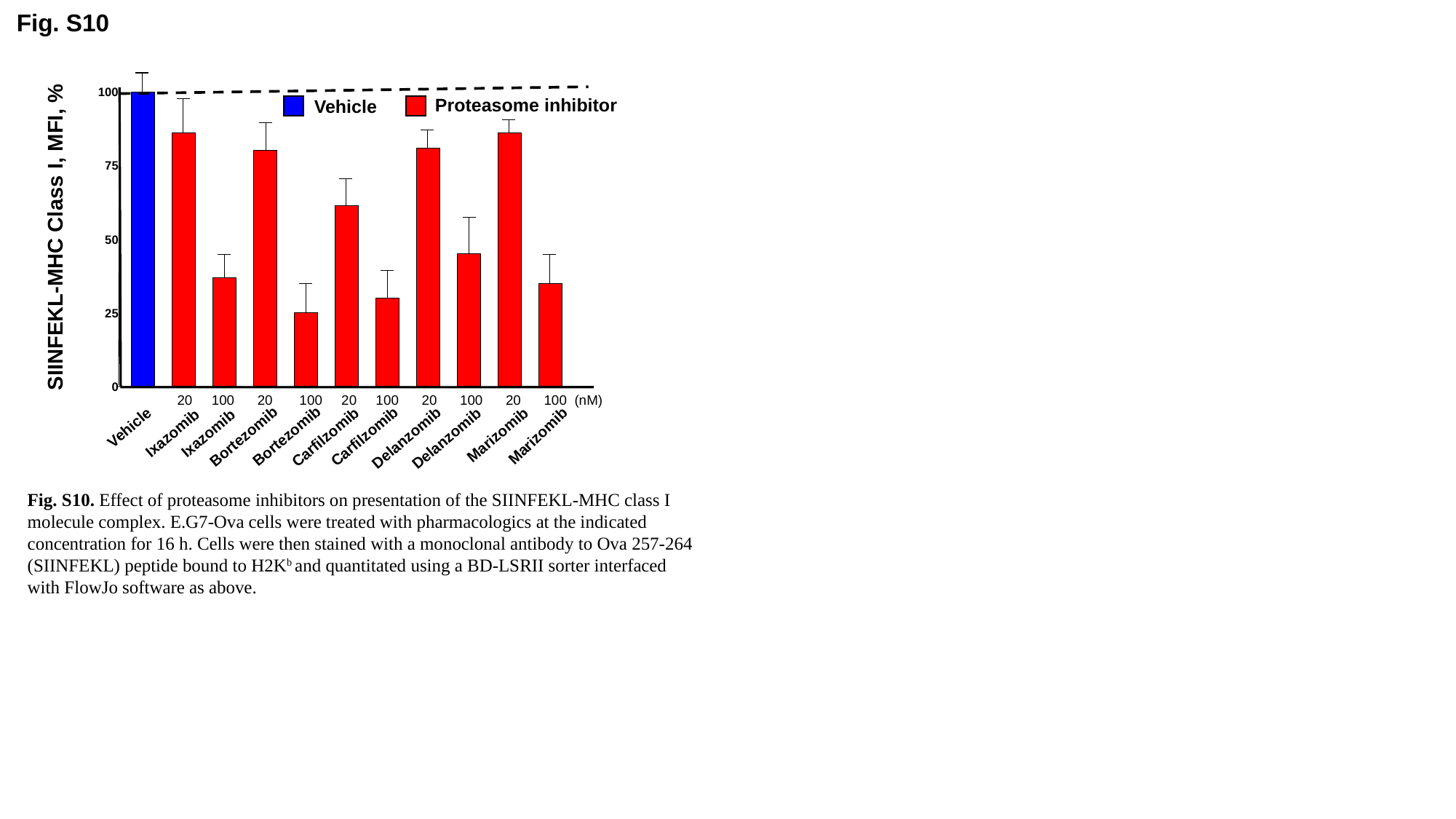

Fig. S10
### Chart
| Category | |
|---|---|Proteasome inhibitor
Vehicle
SIINFEKL-MHC Class I, MFI, %
 20 100 20 100 20 100 20 100 20 100 (nM)
Vehicle
Ixazomib
Ixazomib
Marizomib
Marizomib
Bortezomib
Carfilzomib
Bortezomib
Carfilzomib
Delanzomib
Delanzomib
Fig. S10. Effect of proteasome inhibitors on presentation of the SIINFEKL-MHC class I molecule complex. E.G7-Ova cells were treated with pharmacologics at the indicated concentration for 16 h. Cells were then stained with a monoclonal antibody to Ova 257-264 (SIINFEKL) peptide bound to H2Kb and quantitated using a BD-LSRII sorter interfaced with FlowJo software as above.
