# Supplementary material for: HDAC6 Inhibition Releases HR23B to Activate Proteasomes, Expand the Tumor Immunopeptidome and Amplify T-cell Antimyeloma Activity
Source: Cancer Res Commun. 2024 Jun 18;4(6):1517–32. doi: 10.1158/2767-9764.CRC-23-0528 (PMC11188874; doi:10.1158/2767-9764.CRC-23-0528)
Supplement: Figure S11 — Fig. S11. Effect of the SGC-UBD253N (catalog number SML-3542, Sigma-Aldrich/Millipore-Sigma, Burlington, MA) is a negative control) probe on SIINFEKL-H2Kb presentation on E.G7-Ova cells (a) and effect of SGC-UBD253N on pan HLA-ABC presentation on three different MM cells (b). SGC-UBD253N is a closely related negative control for SGC-UBD253, a chemical probe for the HDAC6 UBD. E.G7-Ova (a) and MM cells (b) were treated with SGC-UBD253N at indicated concentration for 72h and the relative cell surface of the SIINFEKL-H2Kb complex detected by flow cytometry. Cells were then stained with a monoclonal antibody to SIINFEKL-H2Kb and quantitated using a BD-LSRII sorter interfaced with FlowJo software as above. MMCLs were treated with SGC-UBD253N at the indicated concentrations for 72 h. Cells were then stained with an anti- HLA-ABC (W6/32) antibody and quantitated using a BD-LSRII sorter interfaced with FlowJo software as above. Values represent the average of triplicate measurements. Error bars represent the SD. [file crc-23-0528-s17.pptx]

## Slide 1
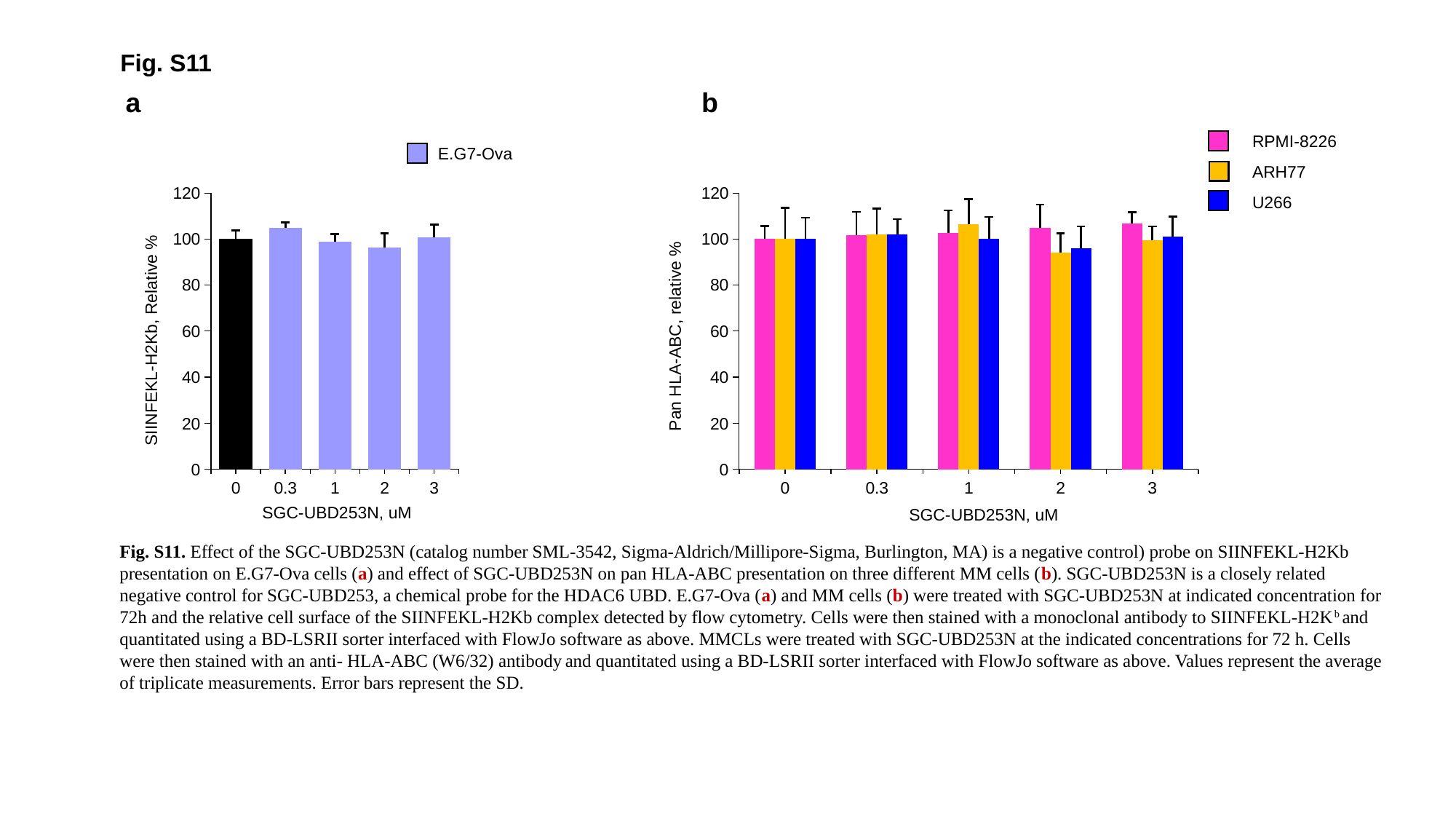

Fig. S11
a
b
RPMI-8226
E.G7-Ova
ARH77
### Chart
| Category | | | |
|---|---|---|---|
| 0 | 100.0 | 100.0 | 100.0 |
| 0.3 | 101.73732479667763 | 101.90936148705565 | 101.98513592802661 |
| 1 | 102.63021283959164 | 106.54237097770537 | 100.22491687854489 |
| 2 | 104.68593182211457 | 93.92935362497893 | 96.02972814394681 |
| 3 | 106.86970064024919 | 99.6068961644297 | 101.00723645609231 |
### Chart
| Category | |
|---|---|
| 0 | 100.0 |
| 0.3 | 104.68331616889806 |
| 1 | 98.83367662203914 |
| 2 | 96.14572605561277 |
| 3 | 100.65911431513904 |U266
Pan HLA-ABC, relative %
SIINFEKL-H2Kb, Relative %
SGC-UBD253N, uM
SGC-UBD253N, uM
Fig. S11. Effect of the SGC-UBD253N (catalog number SML-3542, Sigma-Aldrich/Millipore-Sigma, Burlington, MA) is a negative control) probe on SIINFEKL-H2Kb presentation on E.G7-Ova cells (a) and effect of SGC-UBD253N on pan HLA-ABC presentation on three different MM cells (b). SGC-UBD253N is a closely related negative control for SGC-UBD253, a chemical probe for the HDAC6 UBD. E.G7-Ova (a) and MM cells (b) were treated with SGC-UBD253N at indicated concentration for 72h and the relative cell surface of the SIINFEKL-H2Kb complex detected by flow cytometry. Cells were then stained with a monoclonal antibody to SIINFEKL-H2Kb and quantitated using a BD-LSRII sorter interfaced with FlowJo software as above. MMCLs were treated with SGC-UBD253N at the indicated concentrations for 72 h. Cells were then stained with an anti- HLA-ABC (W6/32) antibody and quantitated using a BD-LSRII sorter interfaced with FlowJo software as above. Values represent the average of triplicate measurements. Error bars represent the SD.
