# Supplementary material for: HDAC6 Inhibition Releases HR23B to Activate Proteasomes, Expand the Tumor Immunopeptidome and Amplify T-cell Antimyeloma Activity
Source: Cancer Res Commun. 2024 Jun 18;4(6):1517–32. doi: 10.1158/2767-9764.CRC-23-0528 (PMC11188874; doi:10.1158/2767-9764.CRC-23-0528)
Supplement: Figure S12 — Fig. S12. a. Effect of the top pharmacologics alone on E.G7-Ova cell viability as measured by LDH release into the culture medium (green). Also shown is the effect of pre-treating the B3Z cells with the pharmacologics then co-culturing with E.G7-Ova cells (red). b. Effect of pre- treatment of B3Z cells with pharmacologics followed by co-culture with E.G7-Ova cells. Cell viability was measured by annexin-V+ staining as above. c. Effect of HDAC6 inhibitors on E.G7-Ova viability. d. Effect of HDAC6 inhibitors on B3Z viability. e. Effect of HDAC6 inhibitors on E.G7-Ova viability after co-culture with B3Z cells at E:T 2:1. [file crc-23-0528-s18.pptx]

## Slide 1
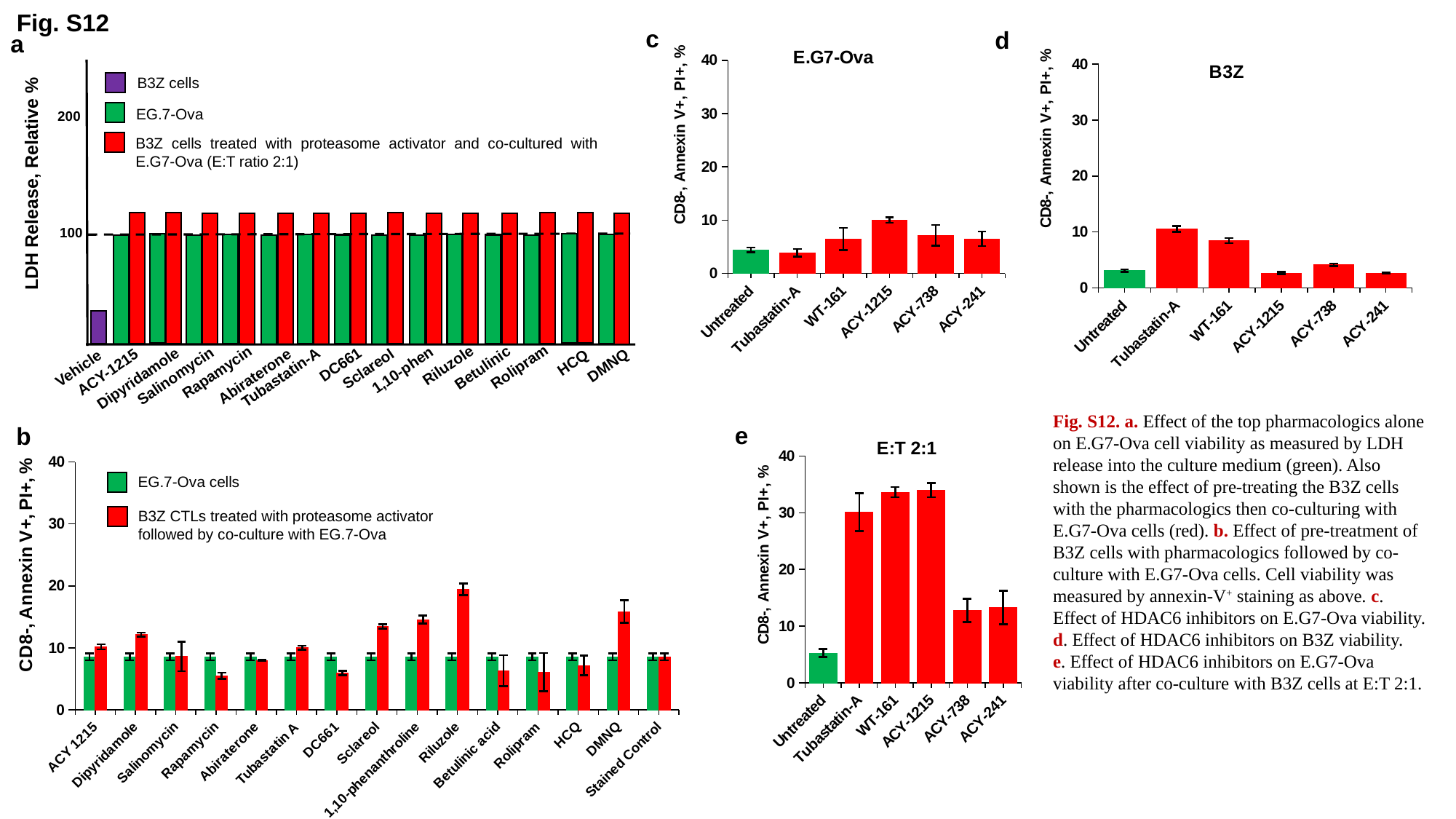

### Chart: E.G7-Ova
| Category | CD8-, Annexin V+, PI+, % |
|---|---|
| Untreated | 4.3933333333333335 |
| Tubastatin-A | 3.8533333333333335 |
| WT-161 | 6.449999999999999 |
| ACY-1215 | 10.01 |
| ACY-738 | 7.156666666666666 |
| ACY-241 | 6.489999999999999 |
Fig. S12
### Chart: B3Z
| Category | CD8-, Annexin V+, PI+, % |
|---|---|
| Untreated | 3.1033333333333335 |
| Tubastatin-A | 10.536666666666667 |
| WT-161 | 8.47 |
| ACY-1215 | 2.6633333333333336 |
| ACY-738 | 4.156666666666666 |
| ACY-241 | 2.67 |c
d
a
B3Z cells
EG.7-Ova
200
B3Z cells treated with proteasome activator and co-cultured with E.G7-Ova (E:T ratio 2:1)
LDH Release, Relative %
100
HCQ
DC661
Riluzole
DMNQ
Betulinic
Rolipram
Vehicle
Sclareol
1,10-phen
ACY-1215
Rapamycin
Salinomycin
Abiraterone
Tubastatin-A
Dipyridamole
### Chart: E:T 2:1
| Category | CD8-, Annexin V+, PI+, % |
|---|---|
| Untreated | 5.256666666666667 |
| Tubastatin-A | 30.100000000000005 |
| WT-161 | 33.63333333333333 |
| ACY-1215 | 33.96666666666666 |
| ACY-738 | 12.783333333333333 |
| ACY-241 | 13.276666666666666 |Fig. S12. a. Effect of the top pharmacologics alone on E.G7-Ova cell viability as measured by LDH release into the culture medium (green). Also shown is the effect of pre-treating the B3Z cells with the pharmacologics then co-culturing with E.G7-Ova cells (red). b. Effect of pre-treatment of B3Z cells with pharmacologics followed by co-culture with E.G7-Ova cells. Cell viability was measured by annexin-V+ staining as above. c. Effect of HDAC6 inhibitors on E.G7-Ova viability. d. Effect of HDAC6 inhibitors on B3Z viability.
e. Effect of HDAC6 inhibitors on E.G7-Ova viability after co-culture with B3Z cells at E:T 2:1.
e
b
### Chart
| Category | | |
|---|---|---|
| ACY 1215 | 8.5675 | 10.1725 |
| Dipyridamole | 8.5675 | 12.125 |
| Salinomycin | 8.5675 | 8.6025 |
| Rapamycin | 8.5675 | 5.5 |
| Abiraterone | 8.5675 | 7.98 |
| Tubastatin A | 8.5675 | 10.0275 |
| DC661 | 8.5675 | 5.933333333333334 |
| Sclareol | 8.5675 | 13.475 |
| 1,10-phenanthroline | 8.5675 | 14.566666666666668 |
| Riluzole | 8.5675 | 19.425 |
| Betulinic acid | 8.5675 | 6.3225 |
| Rolipram | 8.5675 | 6.0875 |
| HCQ | 8.5675 | 7.1499999999999995 |
| DMNQ | 8.5675 | 15.850000000000001 |
| Stained Control | 8.5675 | 8.5675 |EG.7-Ova cells
B3Z CTLs treated with proteasome activator
followed by co-culture with EG.7-Ova
