# Supplementary material for: HDAC6 Inhibition Releases HR23B to Activate Proteasomes, Expand the Tumor Immunopeptidome and Amplify T-cell Antimyeloma Activity
Source: Cancer Res Commun. 2024 Jun 18;4(6):1517–32. doi: 10.1158/2767-9764.CRC-23-0528 (PMC11188874; doi:10.1158/2767-9764.CRC-23-0528)
Supplement: Figure 13 — Fig. S13. MS workflow used to identify MHC class I peptides increased by treatment with HDAC6 inhibitors or bortezomib. [file crc-23-0528-s19.pptx]

## Slide 1
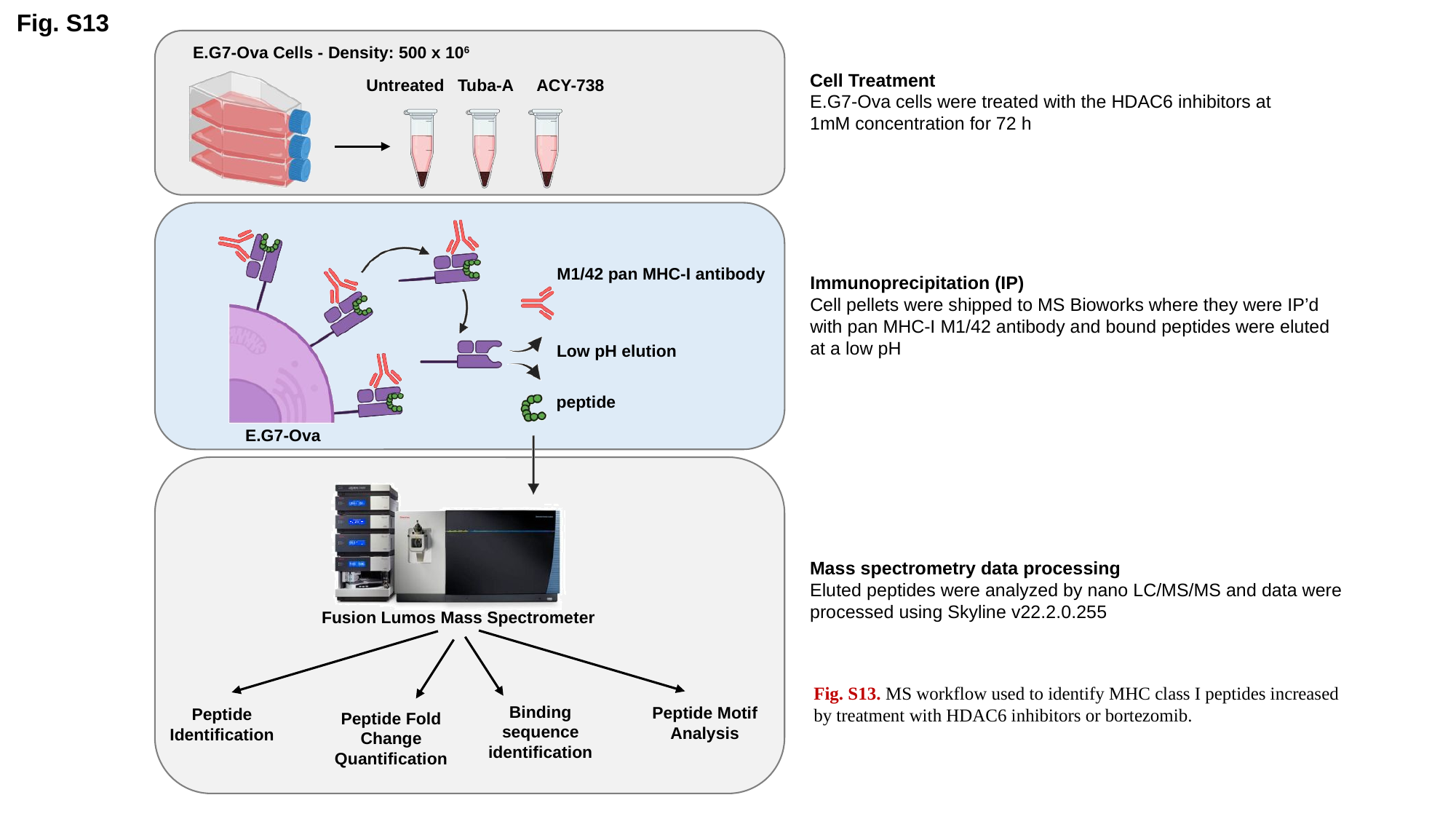

Fig. S13
E.G7-Ova Cells - Density: 500 x 106
Cell Treatment
E.G7-Ova cells were treated with the HDAC6 inhibitors at 1mM concentration for 72 h
Untreated
Tuba-A
ACY-738
M1/42 pan MHC-I antibody
Immunoprecipitation (IP)
Cell pellets were shipped to MS Bioworks where they were IP’d with pan MHC-I M1/42 antibody and bound peptides were eluted at a low pH
Low pH elution
peptide
E.G7-Ova
Mass spectrometry data processing
Eluted peptides were analyzed by nano LC/MS/MS and data were processed using Skyline v22.2.0.255
Fusion Lumos Mass Spectrometer
Fig. S13. MS workflow used to identify MHC class I peptides increased
by treatment with HDAC6 inhibitors or bortezomib.
Binding sequence identification
Peptide Motif Analysis
Peptide Identification
Peptide Fold Change Quantification
