# Supplementary material for: HDAC6 Inhibition Releases HR23B to Activate Proteasomes, Expand the Tumor Immunopeptidome and Amplify T-cell Antimyeloma Activity
Source: Cancer Res Commun. 2024 Jun 18;4(6):1517–32. doi: 10.1158/2767-9764.CRC-23-0528 (PMC11188874; doi:10.1158/2767-9764.CRC-23-0528)
Supplement: Figure S14 — Fig. S14. Effect of HDAC6 inhibitors at the indicated concentrations and times on a. proteasome activity and b. cell viability. Panel c. shows the effect of non-specific HDAC inhibitors on proteasome activity and cell viability. In panels a, b, and c, MM cells (50,000/well) were incubated with each pharmacologic at 1 uM for 72 h. Cell viability was determined using the XTT assay. d. Effect of HAT activators on proteasome activity and cell viability. MM cells (50,000/well) were incubated with each pharmacologic at 1 uM for 72 h. Cell viability was determined using the XTT assay. [file crc-23-0528-s20.pptx]

## Slide 1
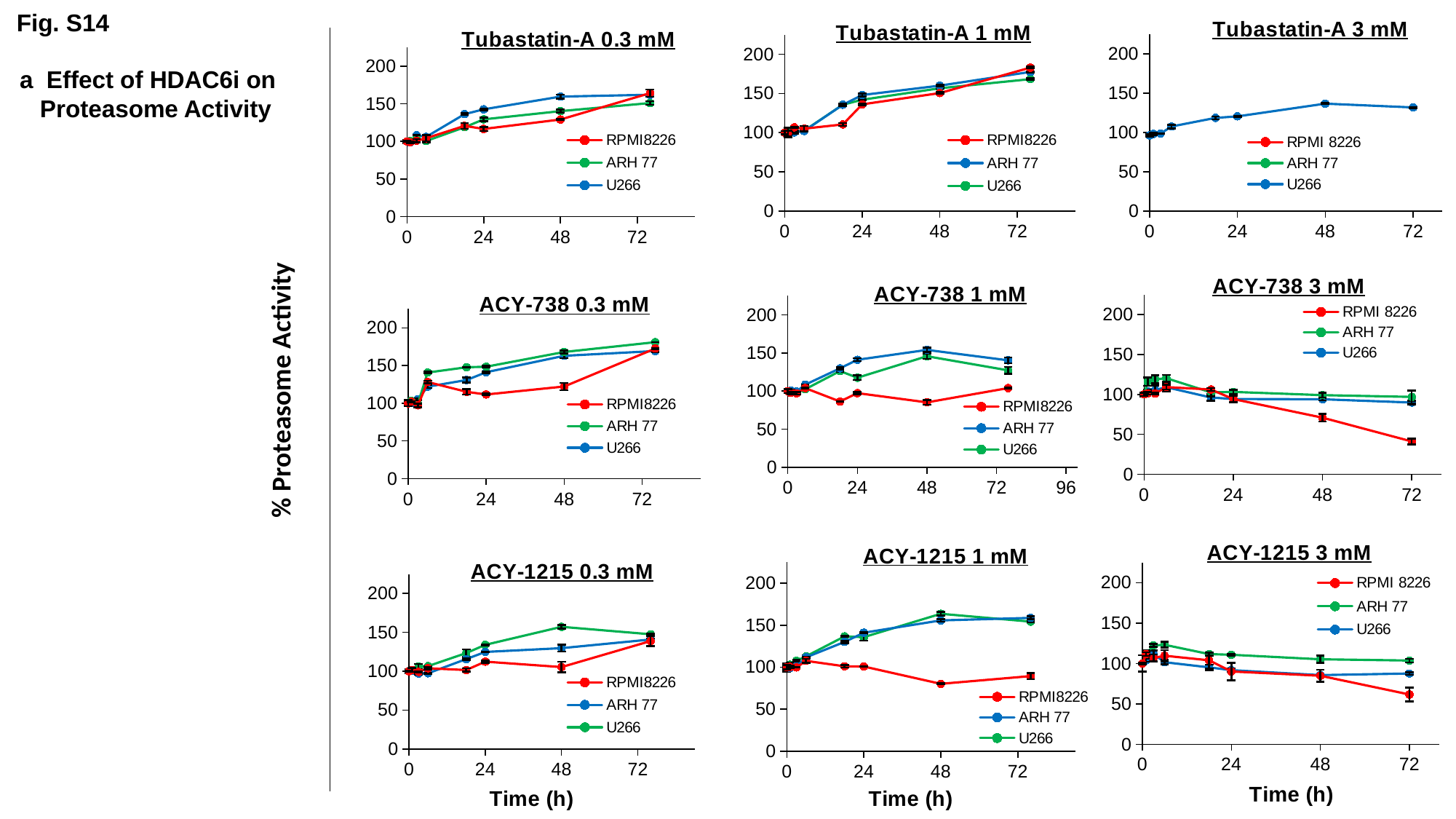

### Chart: Tubastatin-A 3 mM
| Category | | | |
|---|---|---|---|
### Chart: Tubastatin-A 1 mM
| Category | | | |
|---|---|---|---|Fig. S14
### Chart: Tubastatin-A 0.3 mM
| Category | | | |
|---|---|---|---|a Effect of HDAC6i on
 Proteasome Activity
### Chart: ACY-738 3 mM
| Category | | | |
|---|---|---|---|
### Chart: ACY-738 1 mM
| Category | | | |
|---|---|---|---|
### Chart: ACY-738 0.3 mM
| Category | | | |
|---|---|---|---|% Proteasome Activity
### Chart: ACY-1215 3 mM
| Category | | | |
|---|---|---|---|
### Chart: ACY-1215 1 mM
| Category | | | |
|---|---|---|---|
### Chart: ACY-1215 0.3 mM
| Category | | | |
|---|---|---|---|

## Slide 2
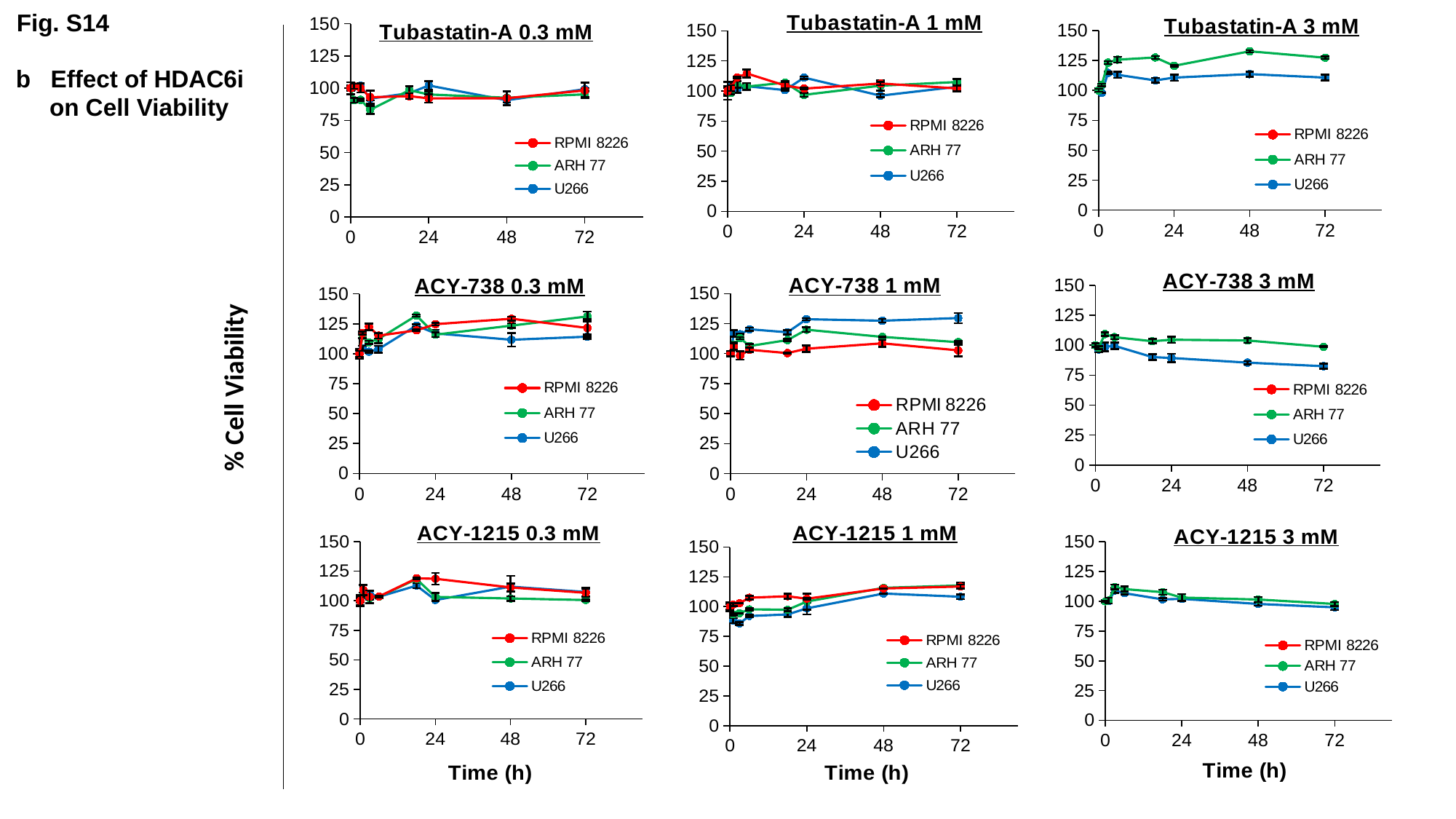

### Chart: Tubastatin-A 0.3 mM
| Category | | | |
|---|---|---|---|
### Chart: Tubastatin-A 1 mM
| Category | | | |
|---|---|---|---|
### Chart: Tubastatin-A 3 mM
| Category | | | |
|---|---|---|---|Fig. S14
b Effect of HDAC6i
 on Cell Viability
### Chart: ACY-738 3 mM
| Category | | | |
|---|---|---|---|
### Chart: ACY-738 1 mM
| Category | | | |
|---|---|---|---|
### Chart: ACY-738 0.3 mM
| Category | | | |
|---|---|---|---|% Cell Viability
### Chart: ACY-1215 3 mM
| Category | | | |
|---|---|---|---|
### Chart: ACY-1215 0.3 mM
| Category | | | |
|---|---|---|---|
### Chart: ACY-1215 1 mM
| Category | | | |
|---|---|---|---|

## Slide 3
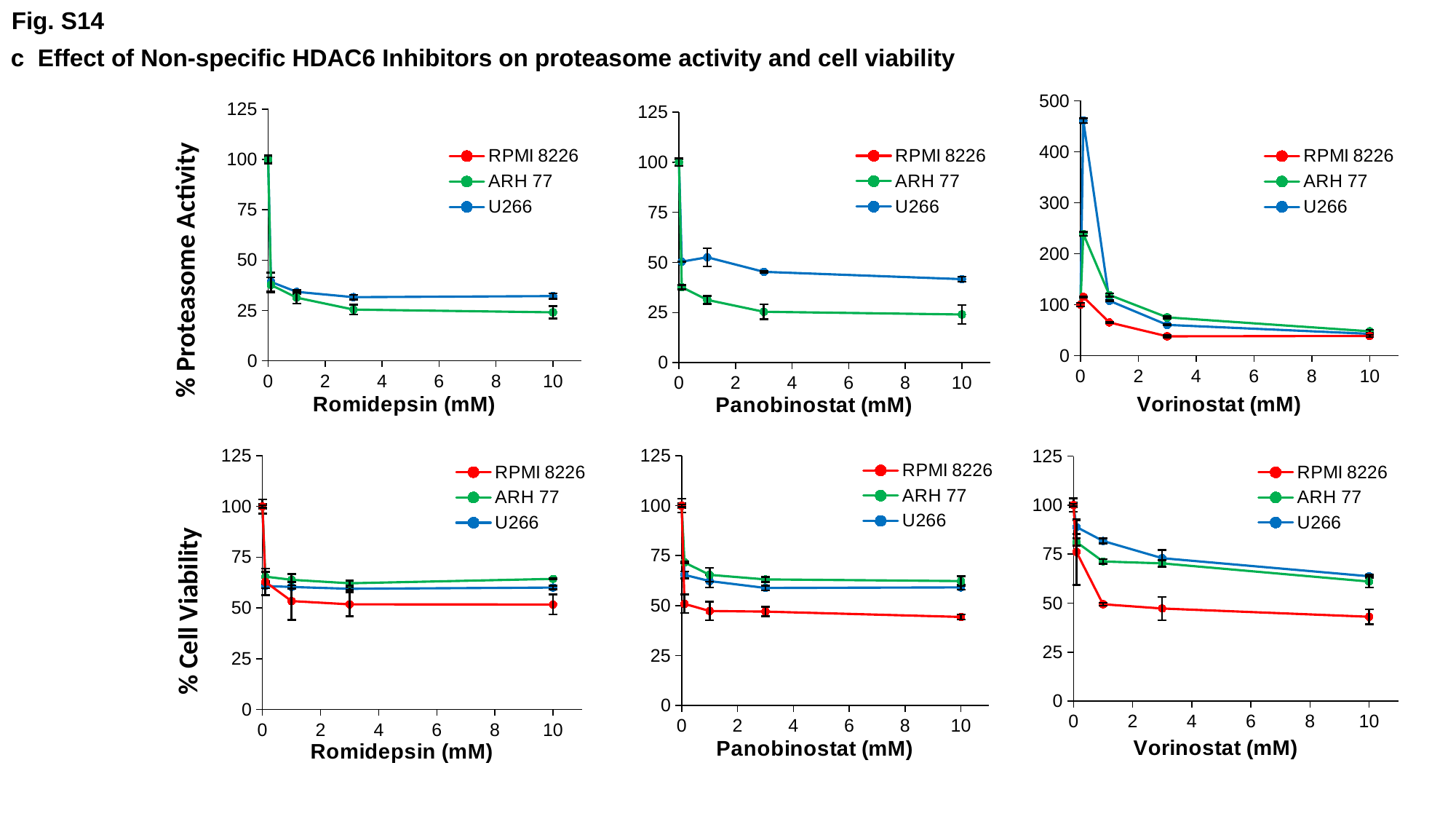

Fig. S14
c Effect of Non-specific HDAC6 Inhibitors on proteasome activity and cell viability
### Chart
| Category | | | |
|---|---|---|---|
### Chart
| Category | | | |
|---|---|---|---|
### Chart
| Category | | | |
|---|---|---|---|% Proteasome Activity
### Chart
| Category | | | |
|---|---|---|---|
### Chart
| Category | | | |
|---|---|---|---|
### Chart
| Category | | | |
|---|---|---|---|% Cell Viability

## Slide 4
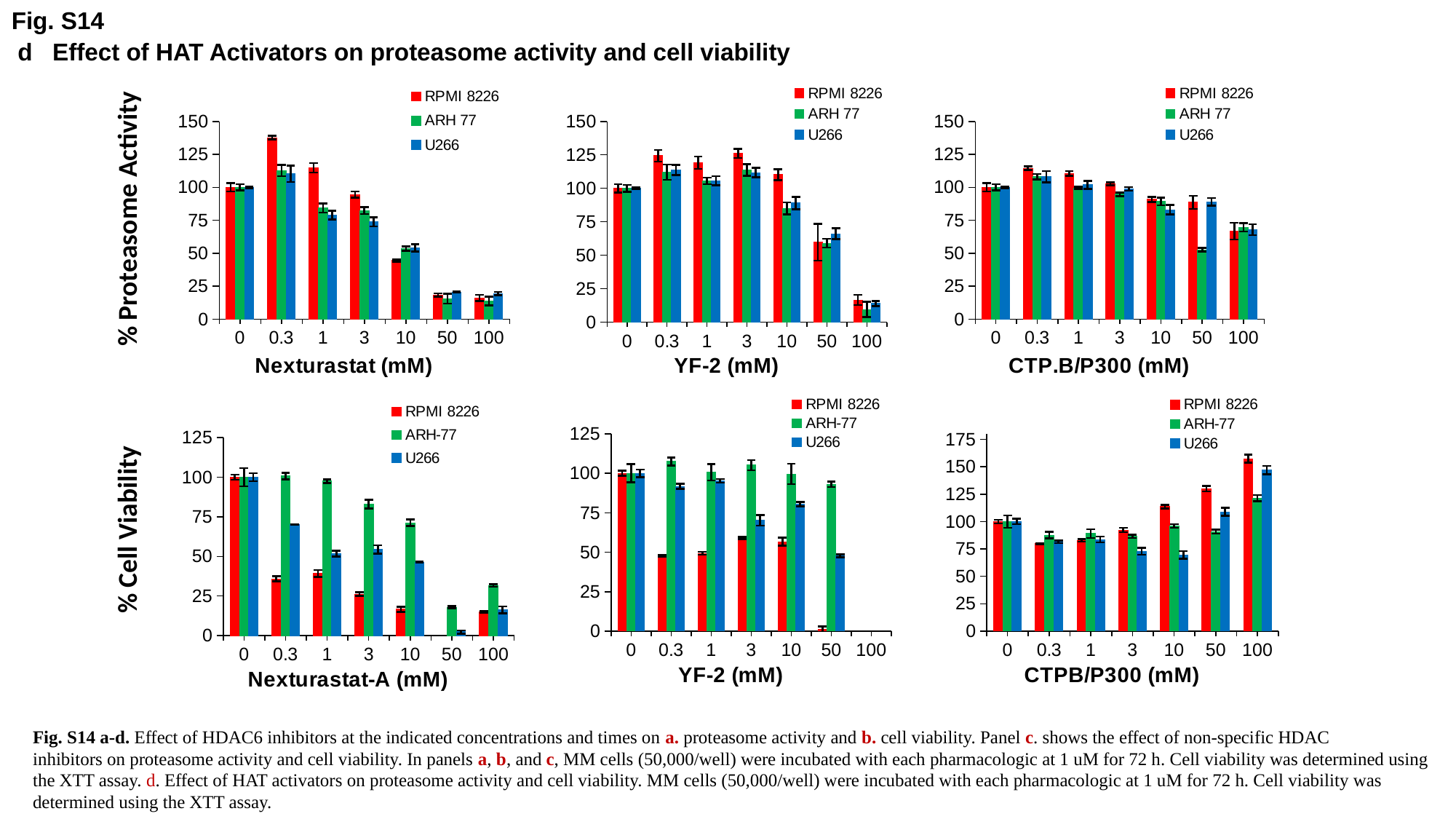

Fig. S14
d Effect of HAT Activators on proteasome activity and cell viability
### Chart
| Category | | | |
|---|---|---|---|
| | 99.9999999999997 | 99.99999999999984 | 99.9999999999997 |
| | 137.65789643016183 | 112.61316277191949 | 110.31333372178165 |
| | 114.76288750603598 | 84.33491857112416 | 78.83415360259673 |
| | 94.4941235708745 | 82.37013818907762 | 73.8845094941083 |
| | 44.47842435608529 | 53.47111285600289 | 54.139432188426774 |
| | 18.26203195193148 | 15.616149398065266 | 20.668702472920618 |
| | 16.15982088026798 | 13.740543410189796 | 19.471034741869435 |
### Chart
| Category | | | |
|---|---|---|---|
| | 99.9999999999997 | 99.99999999999984 | 99.9999999999997 |
| | 124.3391086619305 | 112.07222580737437 | 113.69285260772826 |
| | 119.14961232997425 | 105.52436190470118 | 105.62778917444453 |
| | 126.08115613190797 | 113.73915738677347 | 111.62643713717357 |
| | 110.17823887670303 | 85.02319681850392 | 89.04746438065948 |
| | 59.83923766662046 | 59.05615620102416 | 66.04519537068924 |
| | 16.653215402847547 | 9.530678483893821 | 14.103756583126048 |
### Chart
| Category | | | |
|---|---|---|---|
| | 99.9999999999997 | 99.99999999999984 | 99.9999999999997 |
| | 114.4648491894566 | 108.10371333475308 | 108.10730334499277 |
| | 110.54841986434796 | 99.50459199730409 | 101.8659598184106 |
| | 102.65865234141917 | 94.74666925221479 | 98.7846534053353 |
| | 90.82013872533345 | 89.1846049674315 | 83.04971045001484 |
| | 88.62411969136305 | 52.61699182055439 | 89.00484264274739 |
| | 66.80609064562923 | 69.72344859156485 | 67.89675754740779 |% Proteasome Activity
### Chart
| Category | | | |
|---|---|---|---|
| | 100.00000000000004 | 100.00000000000004 | 99.99999999999999 |
| | 47.67427896290861 | 107.53673735424375 | 91.6946991571593 |
| | 49.3300518367276 | 100.77117479048133 | 95.30316067725688 |
| | 59.00960718847751 | 105.20089756197517 | 70.18646575912074 |
| | 56.66387903030346 | 99.5808750692828 | 80.36771661069504 |
| | 1.0910501646244033 | 93.07794607975636 | 47.69674905257496 |
| | -43.45321213886475 | -0.40447809976280324 | -34.83380530751145 |
### Chart
| Category | | | |
|---|---|---|---|
| | 100.00000000000004 | 100.00000000000004 | 99.99999999999999 |
| | 79.95200427952386 | 87.63360507055994 | 81.68145640686232 |
| | 82.81656867455268 | 89.022820231094 | 83.58883922722754 |
| | 92.28424928788408 | 86.47418644198717 | 72.90039459358151 |
| | 113.6383405646747 | 95.85189418197885 | 69.62674395673304 |
| | 129.8869122308672 | 90.79716118220728 | 108.90611573598446 |
| | 157.45792683266762 | 121.32844115484365 | 146.92670290279395 |
### Chart
| Category | | | |
|---|---|---|---|
| | 100.00000000000004 | 100.00000000000004 | 99.99999999999999 |
| | 35.82850747279662 | 100.66611418866864 | 70.02227363755355 |
| | 39.230349722708695 | 97.56198914750671 | 51.671627221782714 |
| | 26.22239060670554 | 83.0222014700225 | 54.3872966026287 |
| | 16.590220986749028 | 71.2072719280542 | 46.284468597753104 |
| | -22.39973778537373 | 17.96158414261343 | 2.31034811700878 |
| | 14.804526171680827 | 31.605312685711496 | 16.214851753342096 |% Cell Viability
Fig. S14 a-d. Effect of HDAC6 inhibitors at the indicated concentrations and times on a. proteasome activity and b. cell viability. Panel c. shows the effect of non-specific HDAC
inhibitors on proteasome activity and cell viability. In panels a, b, and c, MM cells (50,000/well) were incubated with each pharmacologic at 1 uM for 72 h. Cell viability was determined using the XTT assay. d. Effect of HAT activators on proteasome activity and cell viability. MM cells (50,000/well) were incubated with each pharmacologic at 1 uM for 72 h. Cell viability was determined using the XTT assay.
