# Supplementary material for: HDAC6 Inhibition Releases HR23B to Activate Proteasomes, Expand the Tumor Immunopeptidome and Amplify T-cell Antimyeloma Activity
Source: Cancer Res Commun. 2024 Jun 18;4(6):1517–32. doi: 10.1158/2767-9764.CRC-23-0528 (PMC11188874; doi:10.1158/2767-9764.CRC-23-0528)
Supplement: Figure S15 — Fig. S15. Effect of HDAC6 inhibitors on a. aggresome formation and b. autophagosome formation. RPMI8226 cells were treated with bortezomib and HDAC6 at the indicated concentrations for 16 h. Aggresomes were quantitated by flow cytometry using the cell-based Proteostat® aggresome detection kit (Enzo Life Sciences, Farmingdale, NY). The kit utilizes a molecular rotor dye which while in solution is prevented from fluorescing by free intramolecular rotation along a single central bond. Specifically intercalation of the dye into the cross-β spine of quaternary protein structures typically found in misfolded and aggregated proteins, inhibits the dye’s rotation and leads to a strong fluorescence. b. Autophagosome formation. RPMI8226 cells were treated with bortezomib and HDAC6 at the indicated concentrations for 16 h. Autophagosomes were quantitated by flow cytometry using the Cyto-ID autophagosome detection kit which measures autophagic vacuoles and monitors autophagic flux in lysosomally inhibited live cells using a novel dye that selectively labels accumulated autophagic vacuoles (Enzo Life Sciences). The 488nm-excitable green dye allows for minimal staining of lysosomes while exhibiting bright fluorescence upon incorporation into pre-autophagosomes, autophagosomes, and autolysosomes (autophagolysosomes). All assays were performed in triplicate. Error bars represent the SD of the mean. [file crc-23-0528-s21.pptx]

## Slide 1
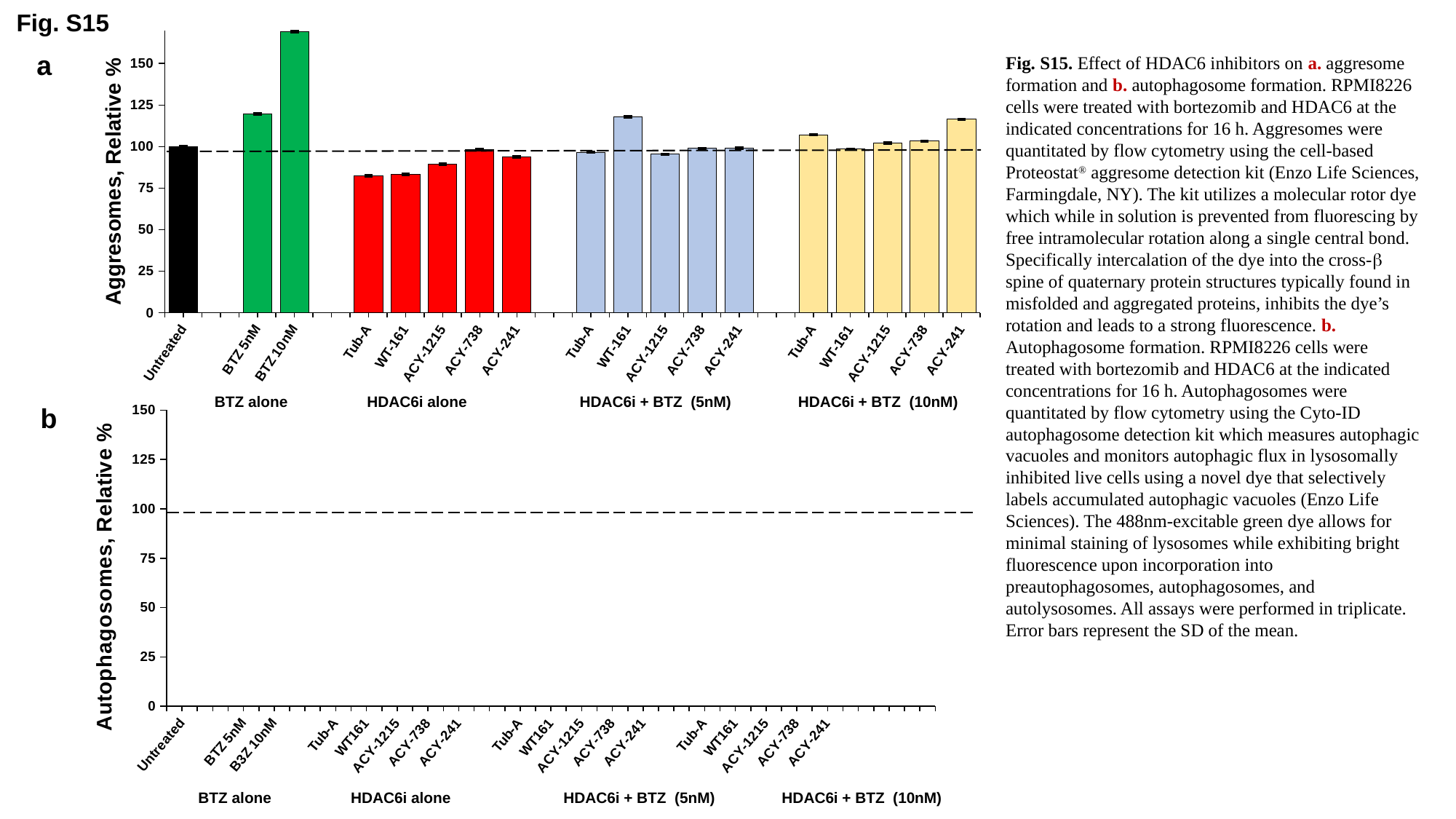

Fig. S15
### Chart
| Category | |
|---|---|
| Untreated | 100.0 |
| | None |
| BTZ 5nM | 119.45392491467577 |
| BTZ 10nM | 169.02730375426623 |
| | None |
| Tub-A | 82.2952218430034 |
| WT-161 | 83.40443686006826 |
| ACY-1215 | 89.50511945392492 |
| ACY-738 | 98.29351535836177 |
| ACY-241 | 93.77133105802048 |
| | None |
| Tub-A | 96.6296928327645 |
| WT-161 | 117.7901023890785 |
| ACY-1215 | 95.39249146757679 |
| ACY-738 | 98.89078498293516 |
| ACY-241 | 98.93344709897612 |
| | None |
| Tub-A | 107.16723549488054 |
| WT-161 | 98.46416382252559 |
| ACY-1215 | 102.13310580204778 |
| ACY-738 | 103.28498293515358 |
| ACY-241 | 116.42491467576792 |a
Fig. S15. Effect of HDAC6 inhibitors on a. aggresome formation and b. autophagosome formation. RPMI8226 cells were treated with bortezomib and HDAC6 at the indicated concentrations for 16 h. Aggresomes were quantitated by flow cytometry using the cell-based Proteostat® aggresome detection kit (Enzo Life Sciences, Farmingdale, NY). The kit utilizes a molecular rotor dye which while in solution is prevented from fluorescing by free intramolecular rotation along a single central bond. Specifically intercalation of the dye into the cross-b spine of quaternary protein structures typically found in misfolded and aggregated proteins, inhibits the dye’s rotation and leads to a strong fluorescence. b. Autophagosome formation. RPMI8226 cells were treated with bortezomib and HDAC6 at the indicated concentrations for 16 h. Autophagosomes were quantitated by flow cytometry using the Cyto-ID autophagosome detection kit which measures autophagic vacuoles and monitors autophagic flux in lysosomally inhibited live cells using a novel dye that selectively labels accumulated autophagic vacuoles (Enzo Life Sciences). The 488nm-excitable green dye allows for minimal staining of lysosomes while exhibiting bright fluorescence upon incorporation into preautophagosomes, autophagosomes, and autolysosomes. All assays were performed in triplicate. Error bars represent the SD of the mean.
Aggresomes, Relative %
 BTZ alone HDAC6i alone HDAC6i + BTZ (5nM) HDAC6i + BTZ (10nM)
### Chart
| Category | |
|---|---|
| Untreated | 100.0 |
| | None |
| BTZ 5nM | 114.21499292786422 |
| B3Z 10nM | 141.65487977369165 |
| | None |
| Tub-A | 75.24752475247524 |
| WT161 | 84.58274398868458 |
| ACY-1215 | 93.14002828854314 |
| ACY-738 | 74.8939179632249 |
| ACY-241 | 15.912305516265912 |
| | None |
| Tub-A | 29.985855728429982 |
| WT161 | 21.57001414427157 |
| ACY-1215 | 100.07072135785006 |
| ACY-738 | 24.115983026874115 |
| ACY-241 | 59.264497878359265 |
| | None |
| Tub-A | 12.446958981612447 |
| WT161 | 34.016973125884014 |
| ACY-1215 | 44.2008486562942 |
| ACY-738 | 63.2956152758133 |
| ACY-241 | 22.065063649222065 |b
 BTZ alone HDAC6i alone HDAC6i + BTZ (5nM) HDAC6i + BTZ (10nM)
