# Supplementary material for: HDAC6 Inhibition Releases HR23B to Activate Proteasomes, Expand the Tumor Immunopeptidome and Amplify T-cell Antimyeloma Activity
Source: Cancer Res Commun. 2024 Jun 18;4(6):1517–32. doi: 10.1158/2767-9764.CRC-23-0528 (PMC11188874; doi:10.1158/2767-9764.CRC-23-0528)
Supplement: Figure S16 — Fig. S16. Effect of anti-HLA antibody on the generation of apoptotic MM cells after treatment with HDCA6 inhibitors and co-culture with autologous T-cells. MM patient CD138+ cells (20,000/ sample) were treated with each HDAC6 inhibitor (1 uM) for 24 h followed by incubated with 0.5 ug/mL mouse anti-HLA ABC antibody W6/32 (catalog number 14-9983-82, Fisher Scientific, Pittsburgh, PA) followed by co-culture with T-cells (E:T 2:1). The percent of apoptotic cells was quantitated by flow cytometry. Values represent the average of triplicate measurements. Error bars represent the SD of the mean. [file crc-23-0528-s22.pptx]

## Slide 1
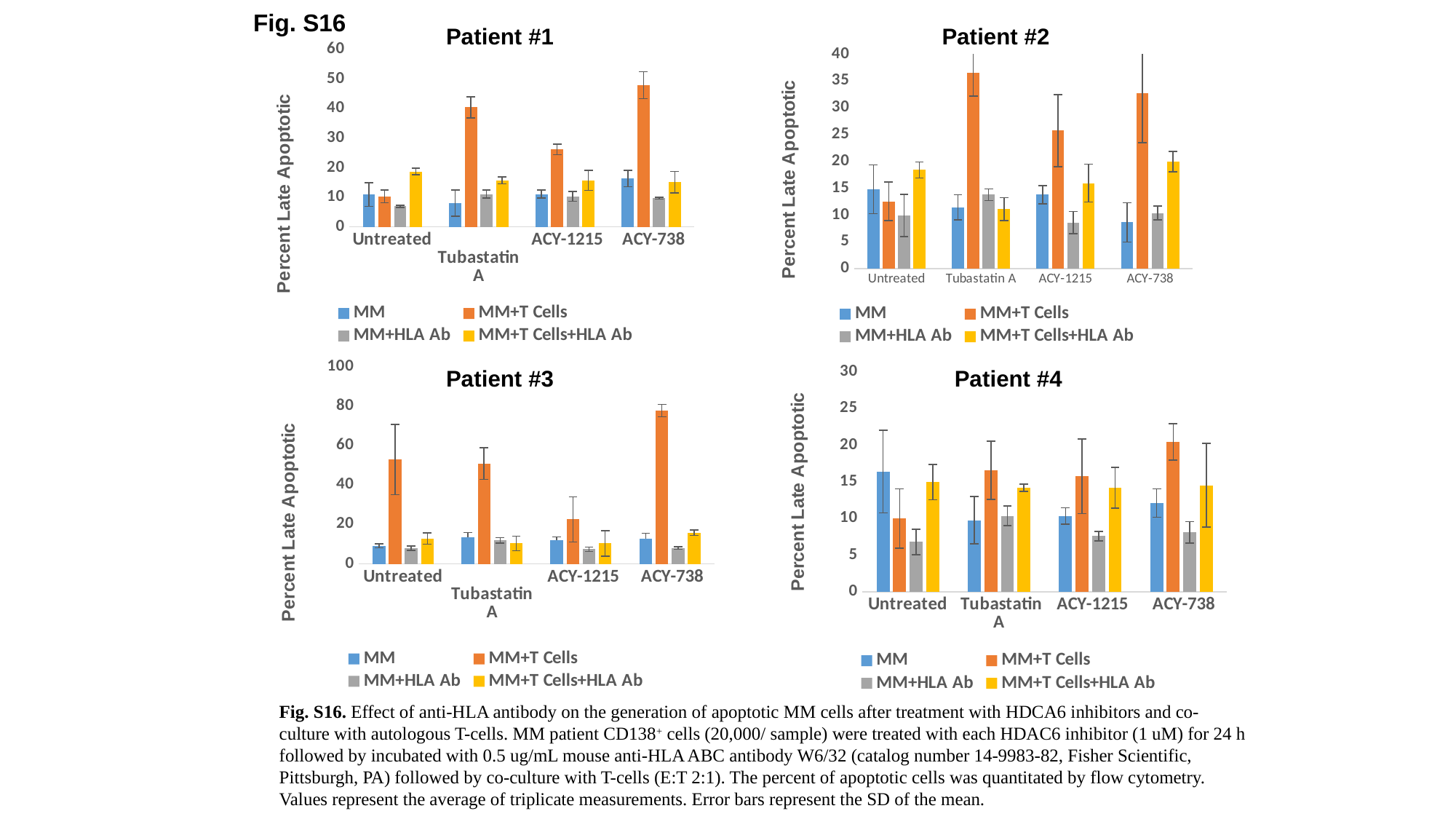

Fig. S16
Patient #1
Patient #2
### Chart
| Category | MM | MM+T Cells | MM+HLA Ab | MM+T Cells+HLA Ab |
|---|---|---|---|---|
| Untreated | 10.93 | 10.306666666666667 | 6.946666666666666 | 18.766666666666666 |
| Tubastatin A | 8.0 | 40.46666666666667 | 11.1 | 15.766666666666666 |
| ACY-1215 | 11.1 | 26.266666666666666 | 10.333333333333334 | 15.766666666666666 |
| ACY-738 | 16.366666666666667 | 47.96666666666667 | 9.673333333333332 | 15.149999999999999 |
### Chart
| Category | MM | MM+T Cells | MM+HLA Ab | MM+T Cells+HLA Ab |
|---|---|---|---|---|
| Untreated | 14.800000000000002 | 12.536666666666667 | 9.93 | 18.433333333333334 |
| Tubastatin A | 11.455 | 36.56666666666666 | 13.799999999999999 | 11.100000000000001 |
| ACY-1215 | 13.799999999999999 | 25.75 | 8.586666666666666 | 15.966666666666667 |
| ACY-738 | 8.633333333333333 | 32.73333333333333 | 10.373333333333335 | 19.96666666666667 |
### Chart
| Category | MM | MM+T Cells | MM+HLA Ab | MM+T Cells+HLA Ab |
|---|---|---|---|---|
| Untreated | 16.400000000000002 | 10.006666666666666 | 6.8 | 14.966666666666667 |
| Tubastatin A | 9.780000000000001 | 16.566666666666666 | 10.363333333333333 | 14.199999999999998 |
| ACY-1215 | 10.363333333333333 | 15.770000000000001 | 7.6000000000000005 | 14.199999999999998 |
| ACY-738 | 12.096666666666666 | 20.433333333333334 | 8.126666666666667 | 14.525000000000002 |
### Chart
| Category | MM | MM+T Cells | MM+HLA Ab | MM+T Cells+HLA Ab |
|---|---|---|---|---|
| Untreated | 9.133333333333333 | 52.96666666666667 | 8.020000000000001 | 12.89 |
| Tubastatin A | 13.533333333333333 | 50.96666666666667 | 11.933333333333332 | 10.393333333333333 |
| ACY-1215 | 11.933333333333332 | 22.599999999999998 | 7.4366666666666665 | 10.393333333333333 |
| ACY-738 | 12.800000000000002 | 77.86666666666666 | 8.013333333333334 | 15.800000000000002 |Patient #3
Patient #4
Fig. S16. Effect of anti-HLA antibody on the generation of apoptotic MM cells after treatment with HDCA6 inhibitors and co-culture with autologous T-cells. MM patient CD138+ cells (20,000/ sample) were treated with each HDAC6 inhibitor (1 uM) for 24 h followed by incubated with 0.5 ug/mL mouse anti-HLA ABC antibody W6/32 (catalog number 14-9983-82, Fisher Scientific, Pittsburgh, PA) followed by co-culture with T-cells (E:T 2:1). The percent of apoptotic cells was quantitated by flow cytometry. Values represent the average of triplicate measurements. Error bars represent the SD of the mean.
