# Supplementary material for: HDAC6 Inhibition Releases HR23B to Activate Proteasomes, Expand the Tumor Immunopeptidome and Amplify T-cell Antimyeloma Activity
Source: Cancer Res Commun. 2024 Jun 18;4(6):1517–32. doi: 10.1158/2767-9764.CRC-23-0528 (PMC11188874; doi:10.1158/2767-9764.CRC-23-0528)
Supplement: Figure S17 — Fig. S17. Effect of HDAC6 inhibitors on the expansion of MM spheroids cultured alone or with T-cells. MM patient CD138+ cells (50,000/ sample) were cultured in 50 μL of Matrigel (Corning) in 96-well plates according to the manufacturer’s protocol. Spheroids were allowed to form and maintained at 37 °C. Cells were then treated with HDAC6 inhibitors (1 uM) for 24 h followed by co-culture with T-cells (E:T 2;1) for another 24 h. Values represent the average of triplicate measurements. [file crc-23-0528-s23.pptx]

## Slide 1
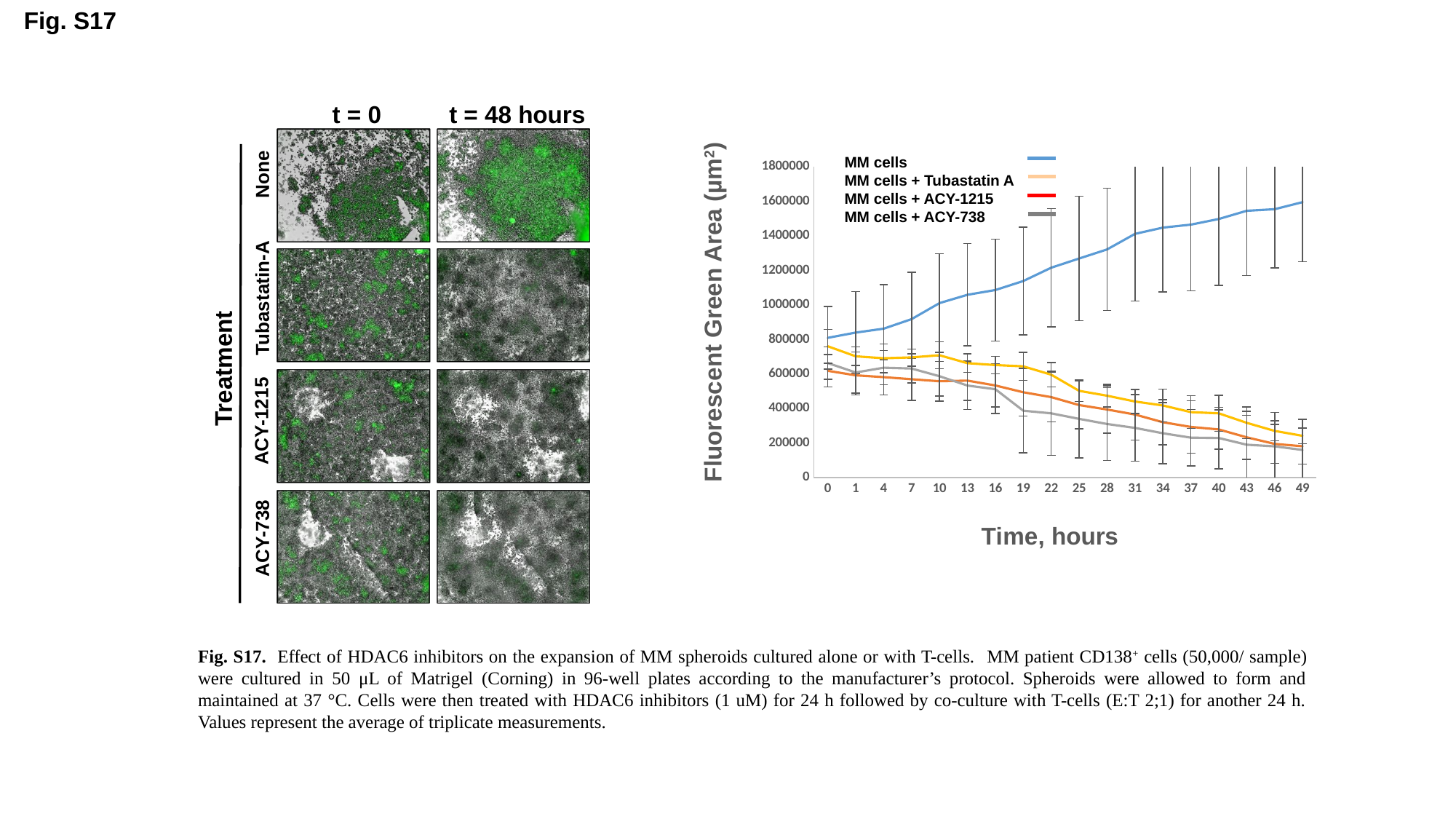

Fig. S17
t = 0
t = 48 hours
MM cells
MM cells + Tubastatin A
MM cells + ACY-1215
MM cells + ACY-738
### Chart
| Category | | | | |
|---|---|---|---|---|
| 0 | 809868.0 | 618689.4 | 663886.7 | 761154.6 |
| 1 | 840269.9 | 592597.1 | 608985.9 | 702894.2 |
| 4 | 862982.3 | 582007.9 | 636875.0 | 691677.2 |
| 7 | 918099.1 | 569902.6 | 632016.3 | 696469.8 |
| 10 | 1011023.0 | 558024.8 | 587318.9 | 708979.1 |
| 13 | 1059095.0 | 561863.7 | 533274.6 | 662893.1 |
| 16 | 1086698.0 | 533721.3 | 512099.3 | 652229.6 |
| 19 | 1138833.0 | 494460.5 | 387480.3 | 644708.6 |
| 22 | 1215921.0 | 466220.7 | 372285.6 | 596310.1 |
| 25 | 1269237.0 | 420294.6 | 340145.5 | 502614.9 |
| 28 | 1321832.0 | 394573.6 | 310684.1 | 474569.6 |
| 31 | 1412008.0 | 365518.3 | 287963.3 | 440739.0 |
| 34 | 1448232.0 | 320919.0 | 256867.2 | 418005.8 |
| 37 | 1465385.0 | 293741.8 | 231430.5 | 379329.5 |
| 40 | 1497977.0 | 279114.5 | 229554.4 | 372271.7 |
| 43 | 1545648.0 | 233448.0 | 190567.4 | 317366.9 |
| 46 | 1554812.0 | 195351.9 | 181084.7 | 270421.0 |
| 49 | 1595798.0 | 181604.2 | 160248.3 | 242136.8 |None
### Chart
| Category |
|---|Tubastatin-A
Fluorescent Green Area (µm2)
Treatment
ACY-1215
ACY-738
Fig. S17. Effect of HDAC6 inhibitors on the expansion of MM spheroids cultured alone or with T-cells. MM patient CD138+ cells (50,000/ sample) were cultured in 50 μL of Matrigel (Corning) in 96-well plates according to the manufacturer’s protocol. Spheroids were allowed to form and maintained at 37 °C. Cells were then treated with HDAC6 inhibitors (1 uM) for 24 h followed by co-culture with T-cells (E:T 2;1) for another 24 h. Values represent the average of triplicate measurements.
