# Supplementary material for: HDAC6 Inhibition Releases HR23B to Activate Proteasomes, Expand the Tumor Immunopeptidome and Amplify T-cell Antimyeloma Activity
Source: Cancer Res Commun. 2024 Jun 18;4(6):1517–32. doi: 10.1158/2767-9764.CRC-23-0528 (PMC11188874; doi:10.1158/2767-9764.CRC-23-0528)
Supplement: Figure S18 — Fig. S18. Correlation of the effect of HDAC6 inhibitors on proteasome activity with the effect on MHC-I antigen presentation. a.) Effect of tubastatin-A on proteasome activity correlated with the effect on pan HLA-ABC antigen presentation. b.) Effect of ACY-738 on proteasome activity correlation with the effect on pan HLA-ABC antigen presentation c.) Effect of ACY-1215 on proteasome activity correlated with the effect on pan HLA-ABC antigen presentation. [file crc-23-0528-s24.pptx]

## Slide 1
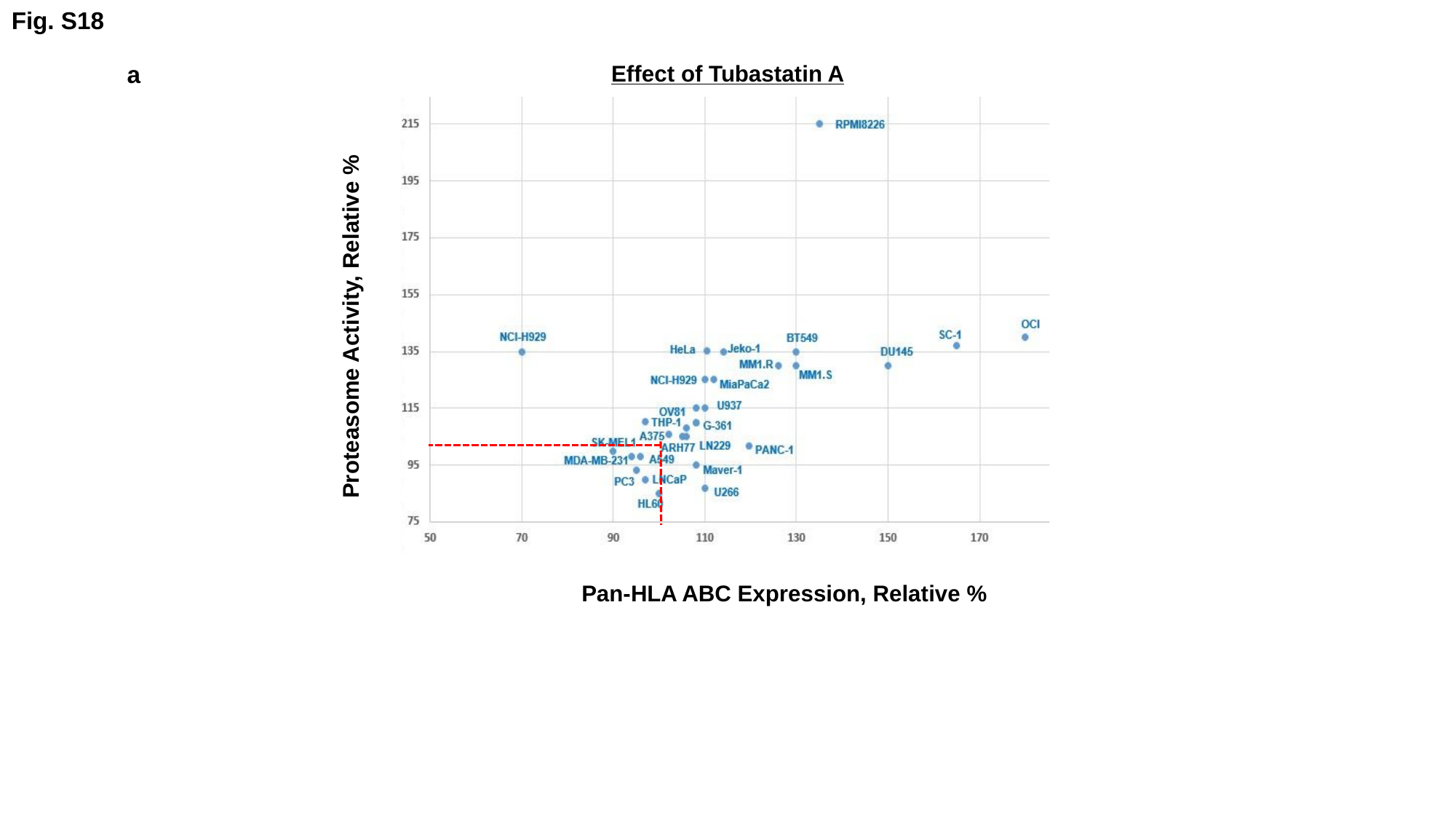

Fig. S18
Effect of Tubastatin A
a
Proteasome Activity, Relative %
Pan-HLA ABC Expression, Relative %

## Slide 2
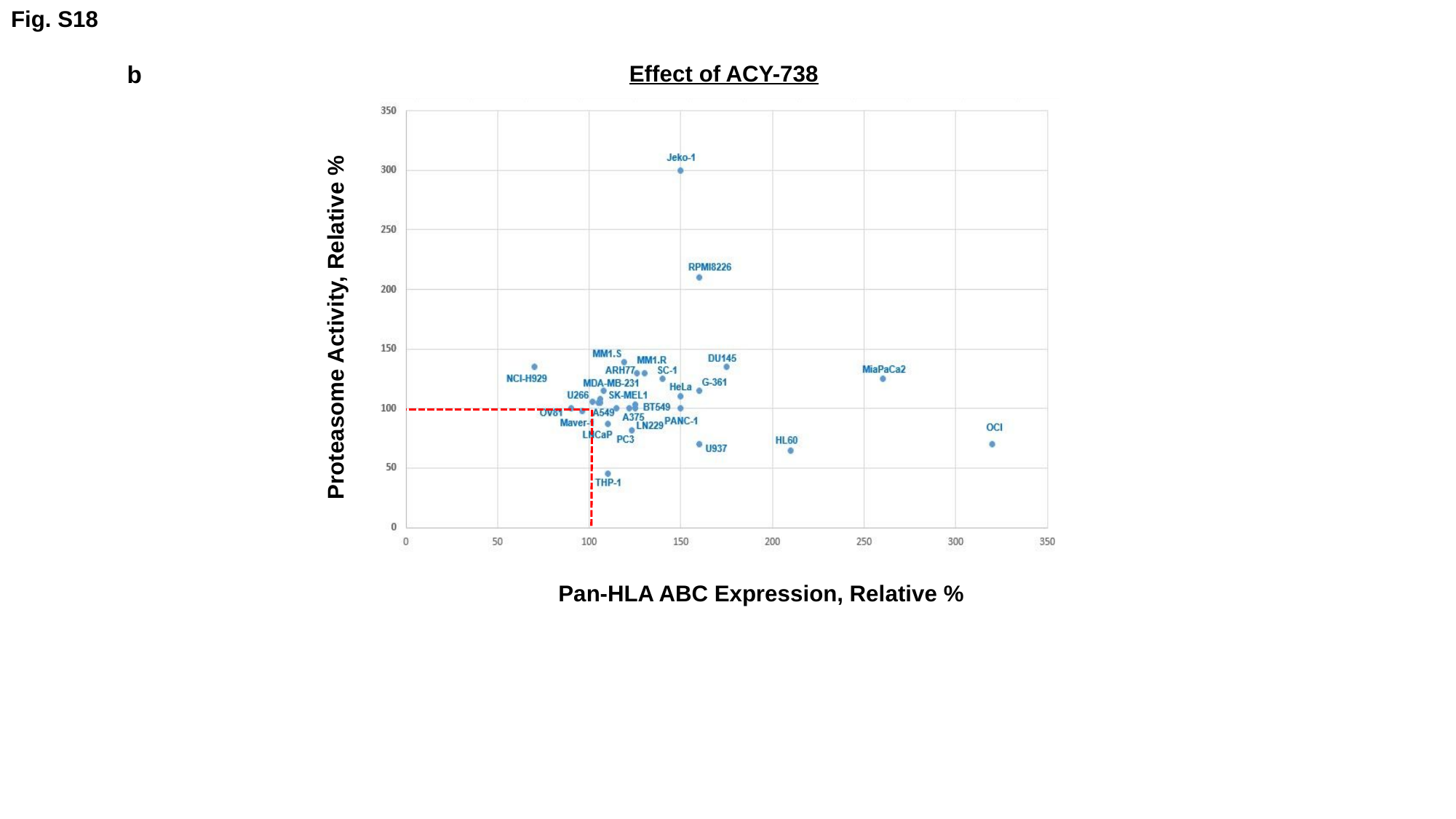

Fig. S18
Effect of ACY-738
b
Proteasome Activity, Relative %
Pan-HLA ABC Expression, Relative %

## Slide 3
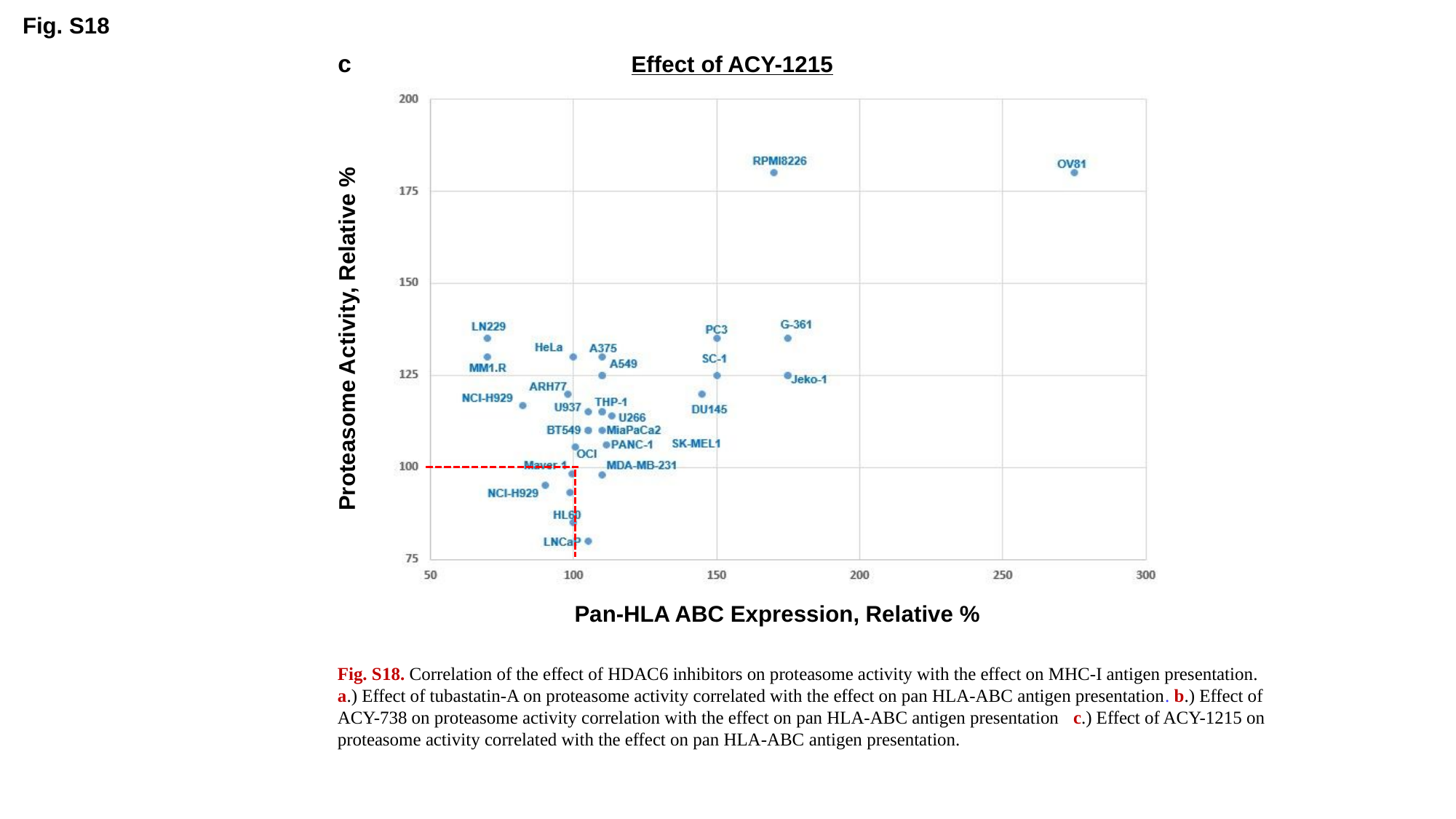

Fig. S18
c
Effect of ACY-1215
Proteasome Activity, Relative %
Pan-HLA ABC Expression, Relative %
Fig. S18. Correlation of the effect of HDAC6 inhibitors on proteasome activity with the effect on MHC-I antigen presentation. a.) Effect of tubastatin-A on proteasome activity correlated with the effect on pan HLA-ABC antigen presentation. b.) Effect of ACY-738 on proteasome activity correlation with the effect on pan HLA-ABC antigen presentation c.) Effect of ACY-1215 on proteasome activity correlated with the effect on pan HLA-ABC antigen presentation.
