# Supplementary material for: HDAC6 Inhibition Releases HR23B to Activate Proteasomes, Expand the Tumor Immunopeptidome and Amplify T-cell Antimyeloma Activity
Source: Cancer Res Commun. 2024 Jun 18;4(6):1517–32. doi: 10.1158/2767-9764.CRC-23-0528 (PMC11188874; doi:10.1158/2767-9764.CRC-23-0528)
Supplement: Figure S19 — Fig. S19. Graphical representation to depict the effect of HDAC6 inhibitors on proteasome activity. HDAC6 inhibitors release HR23B which is bound to the HDAC6 BUZ domain. Free HR23B binds and shuttles ubiquitinated cargo proteins to the proteasome. Rad23 binds the proteasome through a UbL (ubiquitin-like) domain and contains UBA (ubiquitin-associated) motifs that bind multi-ubiquitin chains. These domains allow Rad23 to function as a substrate shuttle-factor. Shown is the association of HR23B with the 26S proteasome through interaction with the non-ATPase regulatory subunit 14, also known as Rpn11. The ability of HDAC6 to downregulate HR23B occurs independently of its deacetylase activity. [file crc-23-0528-s25.pptx]

## Slide 1
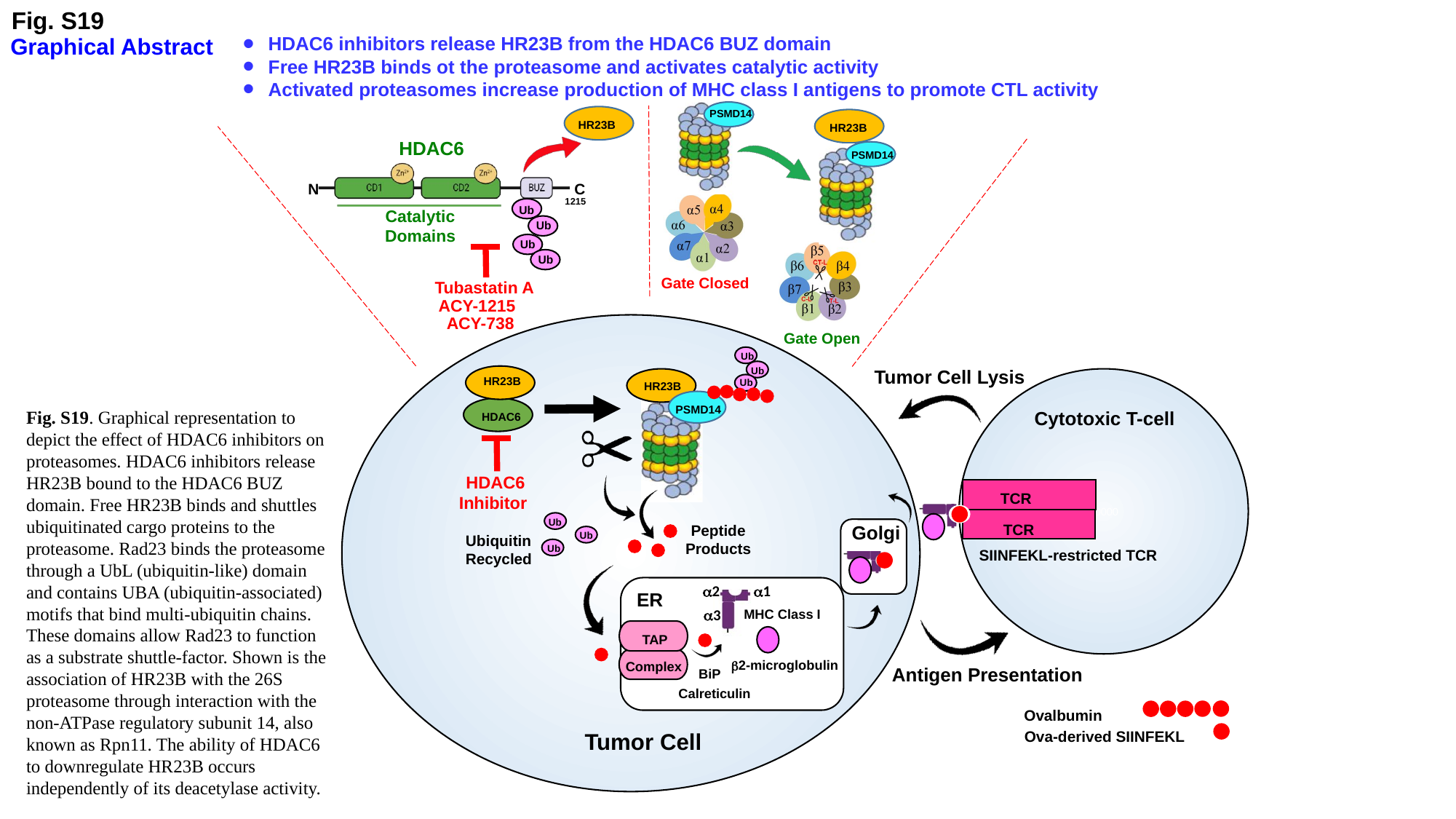

Fig. S19
HDAC6 inhibitors release HR23B from the HDAC6 BUZ domain
Free HR23B binds ot the proteasome and activates catalytic activity
Activated proteasomes increase production of MHC class I antigens to promote CTL activity
Graphical Abstract
PSMD14
HR23B
HR23B
HDAC6
PSMD14
N
C
1215
Catalytic Domains
Ub
Ub
Ub
Ub
Gate Closed
Tubastatin A
ACY-1215
ACY-738
Gate Open
Ub
Ub
Tumor Cell Lysis
00000
HR23B
Ub
HR23B
PSMD14
Fig. S19. Graphical representation to depict the effect of HDAC6 inhibitors on proteasomes. HDAC6 inhibitors release HR23B bound to the HDAC6 BUZ domain. Free HR23B binds and shuttles ubiquitinated cargo proteins to the proteasome. Rad23 binds the proteasome through a UbL (ubiquitin-like) domain and contains UBA (ubiquitin-associated) motifs that bind multi-ubiquitin chains. These domains allow Rad23 to function as a substrate shuttle-factor. Shown is the association of HR23B with the 26S proteasome through interaction with the non-ATPase regulatory subunit 14, also known as Rpn11. The ability of HDAC6 to downregulate HR23B occurs independently of its deacetylase activity.
HDAC6
Cytotoxic T-cell
HDAC6
Inhibitor
TCR
Ub
TCR
Golgi
Peptide
Products
Ub
Ubiquitin
Recycled
Ub
SIINFEKL-restricted TCR
a1
a2
ER
a3
MHC Class I
TAP
Complex
b2-microglobulin
Antigen Presentation
BiP
Calreticulin
Ovalbumin
Ova-derived SIINFEKL
Tumor Cell
