# Supplementary material for: HDAC6 Inhibition Releases HR23B to Activate Proteasomes, Expand the Tumor Immunopeptidome and Amplify T-cell Antimyeloma Activity
Source: Cancer Res Commun. 2024 Jun 18;4(6):1517–32. doi: 10.1158/2767-9764.CRC-23-0528 (PMC11188874; doi:10.1158/2767-9764.CRC-23-0528)
Supplement: Figure S20 — Fig. S20. SDS-PAGE of HDAC6 KO cells. MM cells were either untreated or treated with sgRNA scrambled control or sgRNA specific to HDAC6. Following selection, cell lysates were prepared and electrophoresed on SDS gels, transferred to PVDF. Membranes were probed for HDAC6 using a knockout verified antibody (catalog number ab133493, Abcam, Waltham, MA) a dilution of 1:10,000. [file crc-23-0528-s26.pptx]

## Slide 1
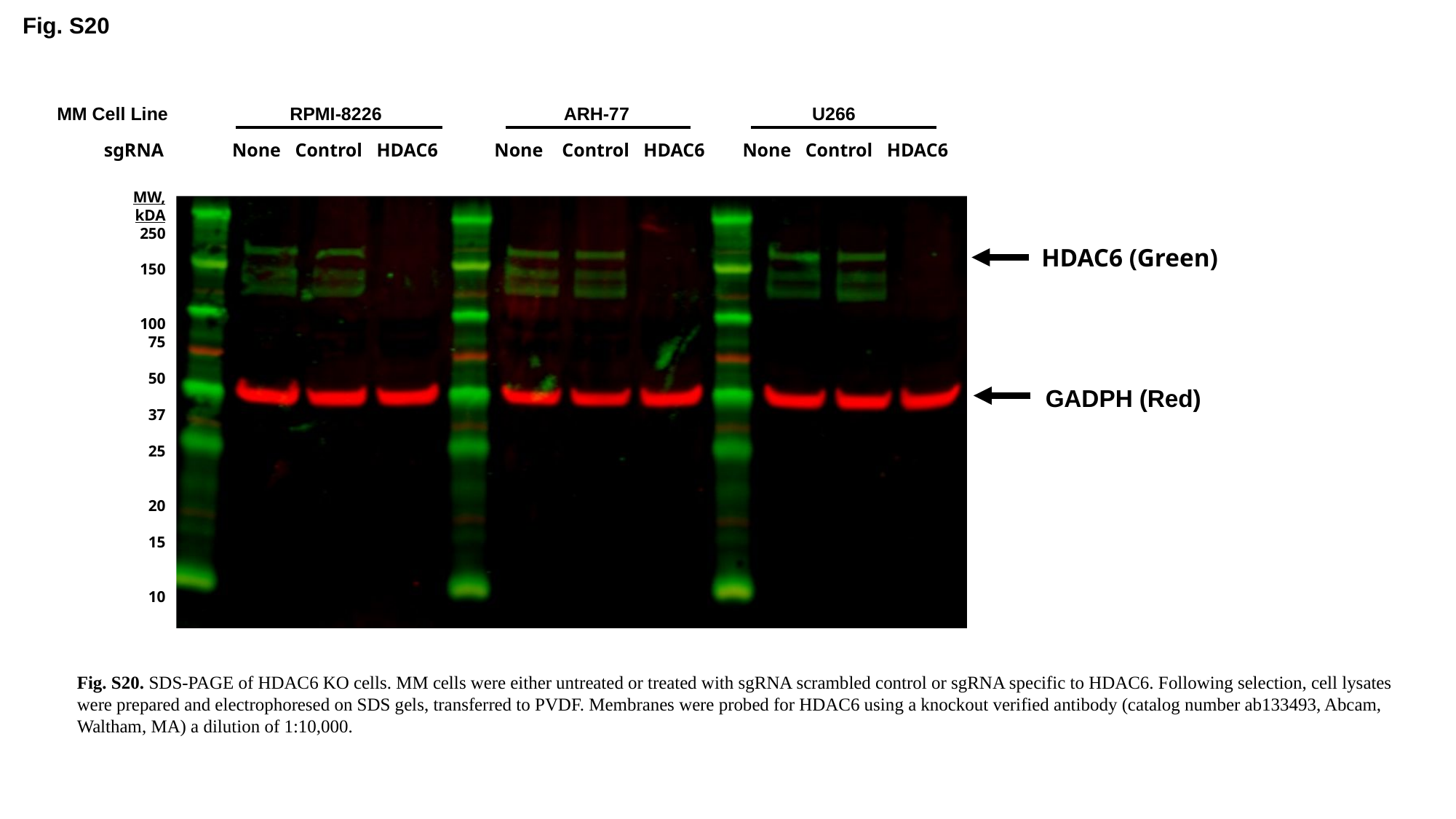

Fig. S20
MM Cell Line RPMI-8226 ARH-77 U266
 sgRNA None Control HDAC6 None Control HDAC6 None Control HDAC6
MW, kDA
250
150
100
75
50
37
25
20
15
10
HDAC6 (Green)
GADPH (Red)
Fig. S20. SDS-PAGE of HDAC6 KO cells. MM cells were either untreated or treated with sgRNA scrambled control or sgRNA specific to HDAC6. Following selection, cell lysates were prepared and electrophoresed on SDS gels, transferred to PVDF. Membranes were probed for HDAC6 using a knockout verified antibody (catalog number ab133493, Abcam, Waltham, MA) a dilution of 1:10,000.
